# A Comprehensive Ab Initio Study of Halogenated A···U and G···C Base Pair Geometries and Energies

**DOI:** 10.3390/ijms24065530

**Published:** 2023-03-14

**Authors:** Rosa M. Gomila, Antonio Frontera, Antonio Bauzá

**Affiliations:** Departament de Química, Universitat de les Illes Balears, Crta. de Valldemossa km 7.5, 07122 Palma de Mallorca, Baleares, Spain

**Keywords:** nucleic acid base pair stability, halogen bonding, hydrogen bonding, supramolecular chemistry, chemical biology

## Abstract

Unraveling the binding preferences involved in the formation of a supramolecular complex is key to properly understand molecular recognition and aggregation phenomena, which are of pivotal importance to biology. The halogenation of nucleic acids has been routinely carried out for decades to assist in their X-ray diffraction analysis. The incorporation of a halogen atom on a DNA/RNA base not only affected its electronic distribution, but also expanded the noncovalent interactions toolbox beyond the classical hydrogen bond (HB) by incorporating the halogen bond (HalB). In this regard, an inspection of the Protein Data Bank (PDB) revealed 187 structures involving halogenated nucleic acids (either unbound or bound to a protein) where at least 1 base pair (BP) exhibited halogenation. Herein, we were interested in disclosing the strength and binding preferences of halogenated A···U and G···C BPs, which are predominant in halogenated nucleic acids. To achieve that, computations at the RI-MP2/def2-TZVP level of theory together with state of the art theoretical modeling tools (including the computation of molecular electrostatic potential (MEP) surfaces, the quantum theory of “Atoms in Molecules” (QTAIM) and the non-covalent interactions plot (NCIplot) analyses) allowed for the characterization of the HB and HalB complexes studied herein.

## 1. Introduction

Non-covalent interactions (NCIs) are of pivotal significance to biology and set the fundamentals of modern chemistry [1,2,3,4]. This is reflected by the number of nonbinding interactions known to date (hydrogen bond, π-π stacking, cation/anion-π) with an important role in several biological processes, such as enzyme inhibition [5], ion transport [6], or protein stability and folding [7]. Similarly to hydrogen bonds (HBs) [8], halogen bonds (HalBs) are currently a well-stablished and well-studied noncovalent force among the supramolecular chemistry community [9,10,11,12,13,14,15]. Both have been described as members of the ‘σ-hole family’ of interactions [16,17,18]. A σ-hole is defined as a region of positive electrostatic potential on a molecule located on the extension of a covalent bond. Common σ-hole examples can be identified along the O–H/C–Br bonds in methanol or bromobenzene molecules. In case of halogens, the σ-hole donor ability increases from F to I [9], being halogen bonds involving I the most enthalpically favored.

In biology, halogens are commonly used in rational drug design and to probe molecular interactions [19,20], thus being of great interest in the field of medicinal chemistry. The halogenation of nucleobases is a useful resource for solving nucleic acid (NA) X-ray crystal structures [21], similarly to the replacement of methionines for selenomethionines in proteins. On the other hand, the halogenation of nucleobases is considered a non-canonical modification, related to the ability of a certain DNA sequence to adopt a different structure (e.g., DNA quadruplex or *i*-motif) from the classical B-DNA form [22]. In this context, several theoretical studies have evaluated the influence of NA halogenation, such as those from Parker [23] and Xu [24] and coworkers, who highlighted the role of HalBs as a relevant interaction in both NA structural and base pairing stabilization. The stability of the interaction is based on different energetic contributions, such as charge transfer [25], dispersion [26], polarization [27] and electrostatics [28,29]. The σ-hole present in the halogen atom serves as electrophilic region for the favorable interaction with an electron rich specie, such as O, N and S as well as a π-system. The size of the σ-hole and polarizability of the halogen atom (which diminishes ongoing from I to F) as well as the electron withdrawing ability of the group where the halogen atom is attached to are key factors that determine the strength and directionality of the interaction. Finally, there is an orbital contribution based on the donation from a lone pair or a π-system belonging to the Lewis base to a σ* orbital from the X–Hal bond [30].

Thorton and collaborators [31] investigated the stability of mono-halogenated and di-halogenated Guanine base pairs. Lastly, Yang and coworkers [32] analyzed the stability of 1-Methyl-5-Halocytosines base pairs (BPs) in the transition from a B-DNA to a DNA *i*-motif structure by using both theoretical and experimental techniques. In addition, several reports exist in the literature where HalBs are functional and relevant binding forces, such as the studies from Carter [33] and Voth [34] and collaborators who engineered DNA Holliday junctions stabilized by HalBs. Moreover, Enninfar and collaborators [35] combined a series of several experimental techniques (including steady-state fluorescence, X-ray crystallography, UV thermal melting and gel electrophoresis) to analyze the effect of NA halogenation on RNA folding. They concluded that the RNA hairpin/duplex ratio was dramatically influenced by not only the presence but also the position of the halogen atom. Furthermore, the studies from Kolář [36] and Auffinger [37] and collaborators are a reliable statistical analysis of the promising potential of HalBs involving nucleic acids. Lastly, we recently demonstrated the role of the HalBs in protein-DNA recognition [38] as well as in protein-RNA stability [39], by combining an inspection of the Protein Data Bank (PDB) [40] with theoretical calculations.

## 2. Results and Discussion

### 2.1. PDB Survey on Halogenated Nucleic Acids

**Figure 1:**
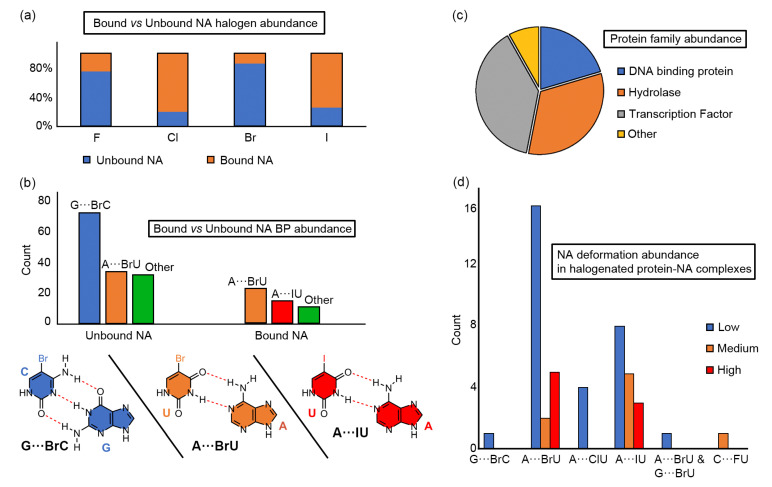
Statistical results from the PDB survey. (**a**) Bound vs. unbound NA halogen abundance, (**b**) bound vs. unbound (to a protein) NA BP abundance, (**c**) protein family abundance, and (**d**) NA deformation abundance in halogenated protein-NA complexes.

**Figure 2:**
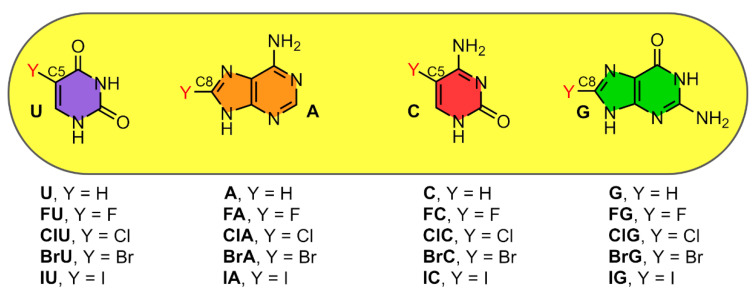
Schematic representation of the nucleobases Uracil (U), Adenine (A), Cytosine (C), and Guanine (G) and their corresponding halogenated derivatives at the C5 and C8 positions used in this study.

Building upon these previous studies, we were motivated to analyze the binding preferences of unbound halogenated base pairs towards interacting either through HB or a combination of hydrogen bonding and halogen bonding interactions. To achieve that, we started by interrogating the PDB to find X-ray NA structures exhibiting halogenated nucleotides. The results are shown in Figure 1 and the complete list of structures found is gathered in the Supplementary Materials section. More in detail, pyrimidine bases (U and C) were found to be halogenated at the C5 position while in purine bases (A and G) the halogenation was carried out at the C8 position (see also Figure 2).

As noticed in Figure 1a, F and Br atoms are more abundant in unbound NA structures than Cl and I, which are more present in protein-NA complexes. In addition, with respect to unbound NAs, the G···BrC base pair is the most abundant one (72 structures), followed by the A···BrU BP (34 structures). The sum of other BP types (e.g., G···IU or BrU···BrU) resulted in 32 structures. On the other side, in the case of protein-NA complexes, the A···BrU BP exhibits the highest abundance (23 structures), followed by the A···IU BP with 15 structures. Finally, other BP combinations (e.g., G···BrC or A···ClU) were less abundant, resulting in a total number of 11 structures (see Figure 1b). Moreover, a schematic representation of the Watson-Crick disposition exhibited by the three most abundant BPs (G···BrC, A···BrU and A···IU) was also included, with indication of the HBs established between both counterparts.

In Figure 1c, a circular plot regarding the abundance of the different protein families bound to halogenated NA structures is shown. As noted, most of the structures belong to Transcription Factors (19 structures), while another important part comes from hydrolases (16 structures). Finally, the rest of protein families involved DNA binding proteins (10 structures) and “other” proteins, which accounted for Gene regulation DNA complexes and RNA binding proteins (4 structures). Lastly, in Figure 1d, a bar plot representing the abundance of the most common halogenated BPs bound to a protein with respect to the deformation degree of the NA structure is shown. As noted, the A···BrU and A···IU BPs are present in NA structures with a different degree of deformation (either low, medium or high), while other BPs (e.g., G···BrC or A···ClU) are present only in canonical B-DNA structures with low deformation. Also, the A···BrU BP showed a preference for low and high deformed NA structures, while in the case of the A···IU BP the tendency observed was a progressive decrease in abundance ongoing from low to high deformed NAs.

Due to the general abundance of G···BrC and A···BrU halogenated BPs, we were interested in studying their strength and binding preferences across several binding modes in their unbound form (without being attained to the sugar moiety and phosphate backbone). Concretely, we have used A, U, G and C and their corresponding halogenated analogous at the C5 (in the case of U and C) and C8 (in the case of A and G) positions (see Figure 2).

For each base pair we have considered the possibility of mono-halogenation (only one base bears a halogen atom) or di-halogenation (both bases bear a halogen atom). In addition, four different binding modes have been studied for each combination (named a, b, c and d) which involve both hydrogen and halogen bonding interactions. These results have been analyzed using several state of the art computational modeling tools such as the calculation of the molecular electrostatic potentials (MEP), the QTAIM (quantum theory of “Atoms in Molecules”) and NCIplot (non-covalent interactions plot) analyses. Finally, calculations on several X-ray structures gathered from the Protein Data Bank (PDB) survey were also performed and the results compared with those retrieved from the computational study.

### 2.2. Preliminary MEP Study

**Figure 3:**
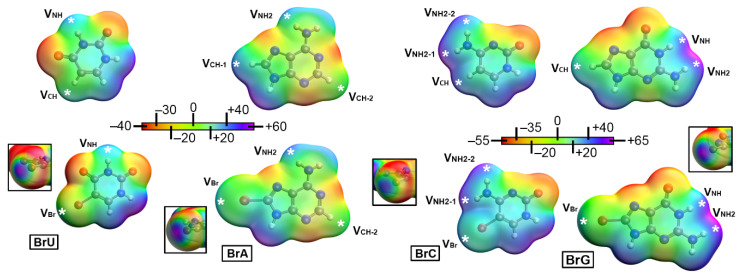
Molecular Electrostatic potential (MEP) surfaces of bromouracil (BrU), bromocytosine (BrC), bromoadenine (BrA), and bromoguanine (BrG). The σ-hole present in the halogen atom (Br as example) is magnified inside the square parts of the figure. The white asterisk indicates where the electrostatic potential value was evaluated (see also Table 1 and Table 2). Surfaces created using an isovalue of 0.001 a.u.

We started our analysis computing the MEP surfaces of U, C, A, and G as well as their corresponding halogenated analogous. In the case of U and C the halogenation was carried out at the C5 position while in the case of A and G the halogenation was performed at the C8 position, in line with the structures obtained from the PDB search. The results are shown in Table 1 and Table 2 and Figure 3.

As noticed, in the case of halogenated U and C the C5 position of the ring (occupied by an halogen atom) varies from negative to positive ongoing from F to I as halogen substituent, thus anticipating a stronger binding upon using heavier halogen atoms (in the case they act as halogen bond donors), as it is commonly known [9]. Interestingly, in the case of C the MEP value over the fluorine atom is almost negligible (V_Hal_ = −0.2 kcal/mol), while negative in U (V_Hal_ = −8.8 kcal/mol), thus indicating that FC is a better halogen bond donor than FU, but at the same time a worse hydrogen bond acceptor. The same behavior can be observed while comparing the rest of MEP values for the halogenated C and U series, being those involving C more positive than those involving U.

In the case of non-halogenated U and C, a very positive MEP value was observed at this position (V_CH_ = +22.0 and +30.7 kcal/mol, respectively) corresponding to a sp^2^ CH group located in the vicinity of a carbonyl group. Lastly, in the case of the NH and NH_2_ groups, very positive electrostatic potential values were observed (ranging between +46 and +37 kcal/mol for both halogenated nucleobases), which decrease upon descending on the halogen group, in line with a decrease in the electron-withdrawing ability of the halogen atom. Compared to their nonhalogenated analogous, these V_NH_ values from halogenated U were more positive, thus indicating an enhancement of this group as HB donor, while in the case of halogenated C they became less positive (V_NH2_ C = +51.5 kcal/mol), therefore suggesting the opposite effect.

On the other hand, in the case of purine bases A and G the electrostatic potential value is given at three different positions, which are the halogen atom (C8 position) and the NH and NH_2_ groups located in the six-membered ring. Firstly, the MEP values at the halogenated position followed the same trend as those involving U and C, thus becoming more positive while descending on the group. Similarly, the MEP value obtained for FA was slightly negative (−1.3 kcal/mol), while in the case of FG it resulted in −6.3 kcal/mol. The rest of the halogen series exhibited a positive MEP value being those involving halogenated A (HalA) more positive than those involving halogenated G.

On the other hand, this position exhibited a positive electrostatic potential value of +28.9 (V_CH-1_) and +23.2 kcal/mol (V_CH_) in the non-halogenated A and G bases, respectively. Also, in the case of non-halogenated A, there is an additional CH group (CH-2 in Table 2), which exhibited a positive MEP value of +7.5 kcal/mol. This value increased on the HalA derivatives to +10 kcal/mol, thus enhancing the HB donor ability of this CH group. Finally, the electrostatic potential over the NH_2_ group of the A ring slightly varies from the non- halogenated base (V_NH2_ = +36.4 kcal/mol) to the HalA series, which exhibit very similar energy values ranging between +37 and +39 kcal/mol.

G also presents three main electrophilic sites, being the C8 position (discussed above) and the NH_2_ and NH groups. As noticed in Table 2, the electrostatic potential values of the NH_2_ group are larger (near +60 kcal/mol) compared to those involving the NH group (around +50 kcal/mol), indicating a higher HB donor ability of the former. Also, while compared to non-halogenated G, they are of similar magnitude, thus, no enhancing or diminishing effect was observed for these two groups.

### 2.3. Energetic Study

**Figure 4:**
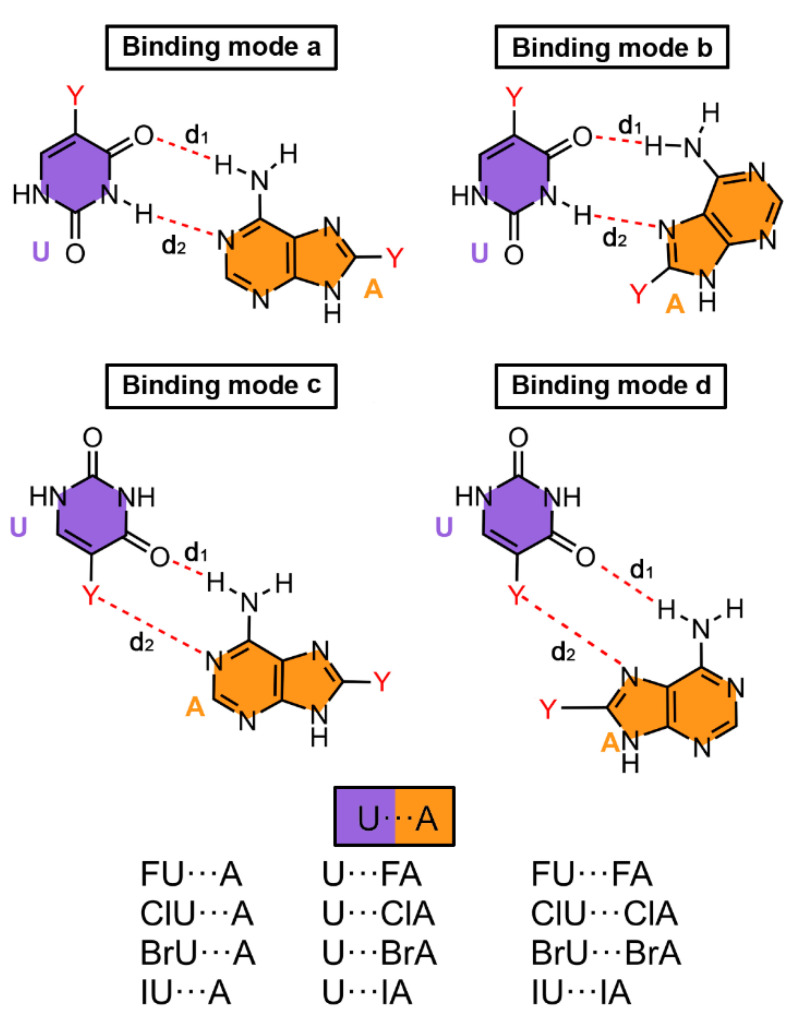
Schematic representation of the binding modes studied for the U···A halogenated BP. Intermolecular distances (d_1_ and d_2_) are gathered in Table 3.

**Figure 5:**
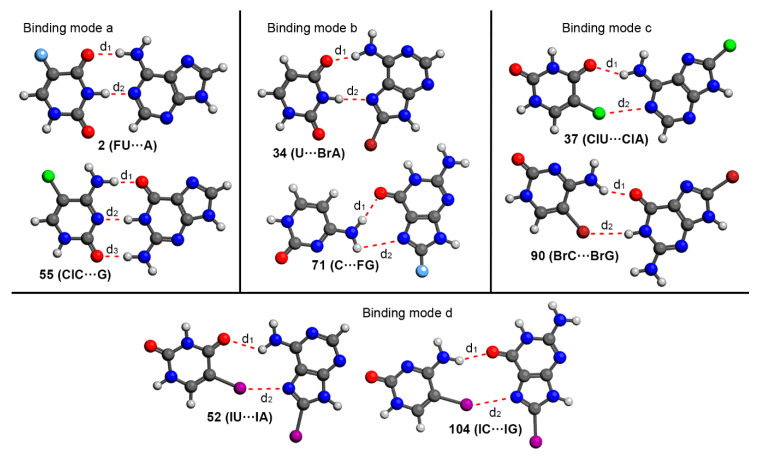
Optimized geometries (RI-MP2/def2-TZVP level of theory) of some representative complexes studied herein. Intermolecular distances (d_1_, d_2_ and d_3_) are gathered in Table 3 and Table 4.

We have In Table 3 the BSSE corrected interaction energies of HB and HalB complexes involving the different binding modes for the U···A BP are shown. In addition, Figure 4 gathers a scheme regarding the four binding modes considered and Figure 5 shows some representative geometries of optimized complexes (RI-MP2/def2-TZVP level of theory).

As noticed in Figure 4, we considered four main different binding modes. Binding modes a and b involve two main HBs and represent the classical Watson-Crick and Hoogsteen interaction patterns, respectively. On the other hand, binding modes c and d were proposed to evaluate the stability of halogenated BPs where both hydrogen bonding and halogen bonding interactions coexist (one HB and one HalB per BP).

Firstly, in all of the cases negative and moderately large binding energy values were obtained, ranging from −14.8 to −3.7 kcal/mol. Secondly, complexes involving binding modes c and d (**27** to **52**, implicating one HB and one HalB) obtained lower binding energy values than those complexes involving binding modes a and b (**1** to **26**, involving two HBs), thus being the classical Watson-Crick and Hoogsteen BP patterns preferred over the other non-canonical binding modes.

The stability of complexes involving binding mode a (**1** to **13**) ranged between −14.1 and −13.6 kcal/mol. Halogenation on U vs. A yielded a slightly more stable BP in the case of the former (e.g., complex **3 (ClU···A)** = −14.1 kcal/mol and complex **7 (U···ClA)** = −13.6 kcal/mol). Interestingly, the HalU···A series obtained more favorable interaction energy values than the non-halogenated U···A BP, therefore reinforcing the BP stability upon halogenation, in agreement with the MEP analysis discussed above. When both counterparts were halogenated, the energy obtained was similar to that for the HalU···A BPs (e.g., complex **11 (ClU···ClA)** = −14.0 kcal/mol). Besides, no clear tendency in the stability of the BPs was observed ongoing from lighter to heavier halogens, resulting in very similar energies among this set of complexes.

For complexes involving binding mode b (**14** to **26**), the binding energies obtained lied between −14.8 and −11.2 kcal/mol. Interestingly, in this case the halogenation on U resulted in an increase of the BP stability of around 3 kcal/mol compared to those BPs halogenated in A (e.g., complex **17 (BrU···A)** = −14.8 kcal/mol and complex **21 (U···BrA)** = −11.4 kcal/mol) as well as those that were di-halogenated (e.g., complex **25 (BrU···BrA)** = −11.5 kcal/mol), being the more stable combination. In addition, halogenation on U also resulted in more stable BP when compared to the non-halogenated U···A BP (complex **14** = −14.4 kcal/mol), similarly to that observed for complexes involving binding mode a. These results are also in line with the MEP analyses, which showed an increase in the electrostatic potential of the NH group in HalU, thus strengthening the BP stability. Also, a little influence of the halogen atom used on the stability of these BPs was observed, without noticing clear tendencies.

Complexes involving binding mode c (**27** to **39**) obtained binding energy values comprised between −8.4 and −4.2 kcal/mol. In this family of complexes, the halogenation on U resulted in a decrease of BP stability (e.g., complex **27 (U···A)** = −8.3 kcal/mol and complex **30 (BrU···A)** = −5.8 kcal/mol), mainly due to a decrease of the electrostatic potential at this position. This becomes more accentuated while increasing the electronegativity of the halogen atom (poor HalB donor ability, as anticipated by the MEP analysis). Complex **31** deserves special mention, since the MEP value over the I atom in IU was more positive than that for U (+23.2 and +22.0 kcal/mol, respectively), however, the latter obtained a more stable BP energy. This is likely due to a counterbalance effect between the increase in the electron acceptor ability (provided by the iodine’s σ-hole) and the poor electron acceptor capacity of the sp^2^ O atom (due to the electron-withdrawing ability of the halogen atom). Di-halogenated complexes also exhibited a decrease of the BP stability compared to the non-halogenated U···A base pair (e.g., complex **38 (BrU···BrA)** = −6.1 kcal/mol). On the other hand, halogenation on the A resulted in a slightly increase of the BP stability compared to the non-halogenated dimer (e.g., complex **35 (U···IA)** = −8.4 kcal/mol), being the preferred situation.

For complexes involving binding mode d (**40** to **52**) the BP stability ranged between −8.6 and −3.7 kcal/mol. Using as a reference value the non-halogenated BP (complex **40 (U···A)** = −7.4 kcal/mol), the halogenation on U resulted in a decrease of around 0.5 to 3.7 kcal/mol in the BP stability. This decrease became more noticeable when ascending in the halogen group (similarly to that obtained for binding mode c). On the other hand, halogenation on A resulted in an increase in the BP stability compared to the non-halogenated U···A dimer (e.g., complex **34 (U···BrA)** = −8.4 kcal/mol). Finally, di-halogenation reported mixed results, with complexes involving F, Cl and Br showing a decrease in the binding energy compared to the non halogenated BP, while in the case of I a similar stability was obtained (complex **52 (IU···IA)** = −7.4 kcal/mol).

Among the four binding modes studied, complex **14** (binding mode b) involving the Hoogsteen face of A, was the most stable among the non-halogenated BPs (−14.4 kcal/mol), followed by complex **1** (binding mode a) with −13.6 kcal/mol. On the other hand, among the HalU complexes (**2** to **5**, **15** to **18**, **28** to **31** and **41** to **44**), complexes **2** to **5** and **15** to **18** achieved the most favorable binding energy values, being binding mode b the preferred one. In the case of HalA complexes (**6** to **9**, **19** to **22**, **32** to **35** and **45** to **48**), the preferred situation was binding mode a (complexes **6** to **9**). Finally, in the case of using di-halogenated complexes (**10** to **13**, **23** to **26**, **36** to **39** and **49** to **52**), binding mode a was also the preferred interacting pattern (**10** to **13**) achieving larger interaction energy values than the rest of the set. In Figure 5, some optimized complexes corresponding to the halogenated U···A BP (**2**, **34**, **37** and **52**) are shown as representative examples. Binding modes c and d were not generally preferred, with lower interaction energy values than modes a and b. Despite of this, the coexistence of both HBs and HalBs as a source of BP stabilization yield quite stable complexes with energies up to −8.4 kcal/mol.

**Figure 6:**
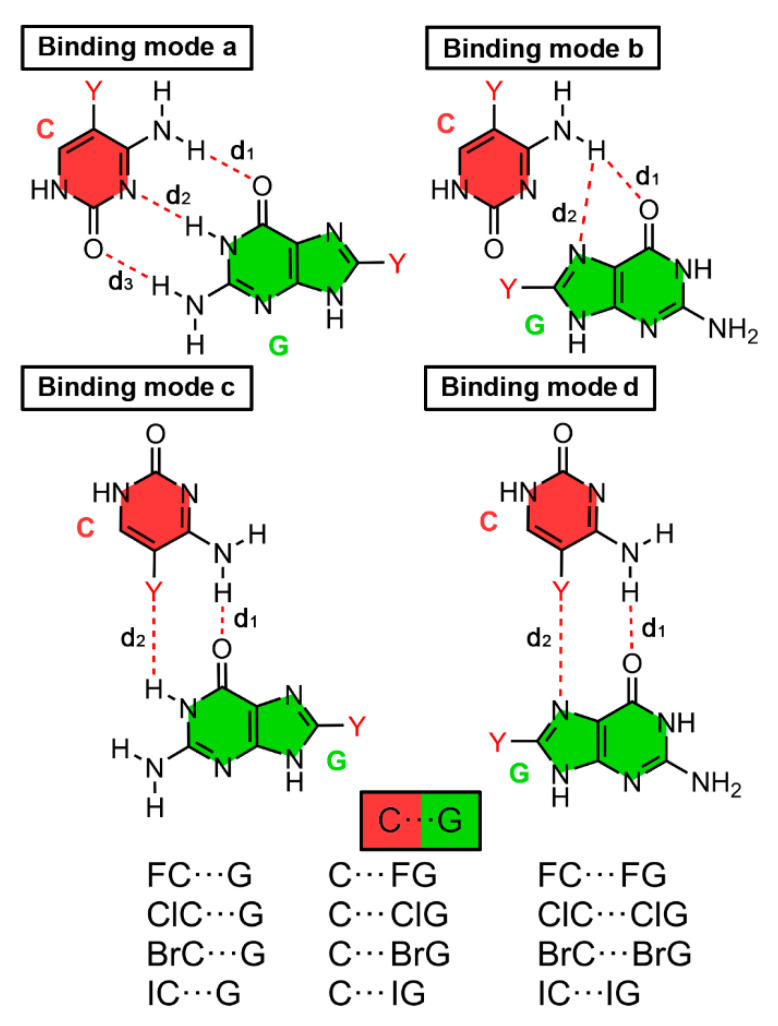
Schematic representation of the binding modes studied for the C···G halogenated BP. Intermolecular distances (d_1_, d_2,_ and d_3_) are gathered in Table 4.

In Table 4 the BSSE corrected interaction energies of HB and HalB complexes involving the different binding modes for the C···G BP are shown. In addition, Figure 5 and Figure 6 gather some representative geometries of the optimized complexes as well as a scheme regarding the four binding modes considered.

Among the four studied binding modes (Figure 6), binding modes a and b (involving the Watson-Crick and Hoogsteen faces of G) rely on three and two HBs, respectively. On the other hand, binding modes c and d are based on a combination of one HB and one HalB, respectively, similarly to that for the U···A BP shown above. All of the complexes studied exhibited a negative and moderately strong binding energy values, comprised between −29.3 to −7.1 kcal/mol. Similarly to that for the U···A BP, complexes involving binding modes c and d (**79** to **104**, implicating two HBs or one HB and one HalB) obtained lower binding energy values than those complexes involving binding modes a and b (**53** to **78**, involving three and one HBs), thus being the latter preferred over the other non-canonical interaction modes.

Firstly, in the case of complexes involving binding mode a (**53** to **65**), the binding energy ranged between −29.3 and −27.1 kcal/mol. In this family of complexes, the halogenation of the U or A did not increase the stability of the BP (e.g., complex **53 (G···C)** = −29.3 kcal/mol, complex **57 (IC···G)** = −28.0 kcal/mol and complex **61 (C···IG)** = −28.5 kcal/mol). This was anticipated by the MEP analysis, which showed less positive potential values for HalC, while in the case of HalG the electrostatic potential values remained similar to that of non-halogenated G. More in particular, halogenation on the G base reported slightly better interaction energies in the cases of F, Cl and I compared to the halogenation on C (e.g., complex **59 (C···ClG)** = −28.6 kcal/mol and complex **55 (ClC···G)** = −28.0 kcal/mol). On the other hand, in the case of di-halogenated complexes, those involving F (**62**) and Br (**64**) achieved a slightly more favorable binding energy value than their Cl and I analogous. Despite of this, none of them exhibited more stability than the non-halogenated C···G BP, in line with the mono-halogenated complexes.

In the case of complexes involving binding mode b (**66** to **78**) the BP stability varied between −15.4 and −9.9 kcal/mol. Concretely, the halogenation on C did not yield a BP with comparable strength with respect to the non-halogenated dimer (complex **66 (C···G)** = −15.4 kcal/mol), similarly to binding mode a. Only in the case of I (complex **70 (IC···G)**) a similar binding energy value was obtained (−15.3 kcal/mol). This is due to a shift in the binding mode from one NH···O HB (shown in complexes **67** to **69**) to a NH···N HB (similar to that obtained for the non-halogenated C···G BP). In the case of G halogenation as well as in di-halogenated complexes a similar behavior was observed, that is, the BP stability increased ongoing from lighter to heavier halogens, being complexes **74 (C···IG)** and **78 (IC···IG)** the most stable ones of their series (−11.5 kcal/mol and −11.4 kcal/mol, respectively).

Complexes encompassing binding mode c (**79** to **91**) achieved a stability ranging between −12.9 and −7.1 kcal/mol. In this set of complexes two different behaviors were observed; on the one hand, halogenation on C resulted in a decrease in BP stability upon descending of the halogen group (e.g., complex **80 (5FC···G)** = −10.6 kcal/mol and complex **83 (IC···G)** = −8.6 kcal/mol). On the other hand, halogenation on G resulted in an increase of the BP stability while descending on the halogen group (e.g., complex **84 (C···FG)** = −10.9 kcal/mol and complex **87 (C···IG)** = −11.2 kcal/mol). Finally, di-halogenation did not show a general trend, being those complexes involving F (**88 (FC···FG)** = −9.2 kcal/mol) and Br (**91 (IC···IG)** = −7.1 kcal/mol) more stable than their Cl and I analogous.

In the case of complexes involving binding mode d (**92** to **104**) the interaction energies ranged between −12.7 and −7.2 kcal/mol. For both mono-halogenated complexes an increase in BP stability was observed while descending in the halogen group (more abrupted in complexes **93** to **96** due to the increase of the HalB donor ability of the nucleobase, as indicated in the MEP analysis), although not enough to surpass the non-halogenated dimer (complex **96 (IC···G)** = −10.8 kcal/mol). In addition, halogenation on G resulted in more favorable BP energies (e.g., complex **94 (ClC···G)** = −9.9 kcal/mol and complex **98 (C···ClG)** = −11.1 kcal/mol). Lastly, di-halogenated complexes exhibited the same trend, however the binding energy values obtained were of lower magnitude.

As a general remark regarding the C···G set of complexes, complex **53** involving binding mode a (Watson-Crick face of G) achieved the most favorable interaction energy value of the non halogenated set. On the other hand, complexes **54** to **65** obtained the largest binding energy values of HalC and HalG BPs, respectively, mainly due to the possibility of stablishing three HBs that this binding mode offers. If this binding mode is not considered, complexes **67** to **70** were the most favorable ones involving HalC (binding mode b), while in the case of halogenated G very similar interaction energies were obtained among binding modes b, c and d, thus showing no preference in binding. Finally, in the case of di-halogenated C···G complexes, binding mode b was preferred over the other two (c and d), which obtained similar results.

### 2.4. QTAIM and NCIplot Analyses

**Figure 7:**
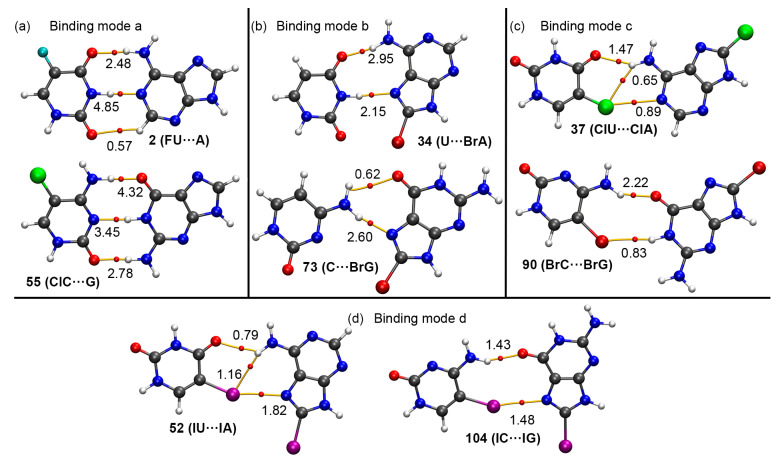
Distribution of critical points and bond paths in several halogenated BPs. Only the intermolecular bond critical points (BCPs) involving hydrogen bonding and/or halogen bonding interactions are shown, denoted by red dots. The bond paths connecting bond critical points are also represented. The value of the density at the BCPs characterizing the HBs and HalBs (ρ·10^2^, a.u.) is also indicated in a.u.

**Figure 8:**
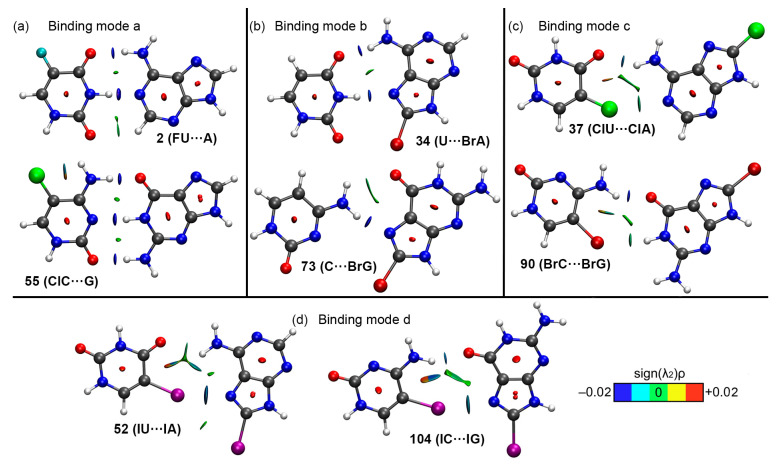
Intra– and Intermolecular NCIplot isosurfaces in several halogenated BPs. NCIplot colour range −0.02 a.u. ≤ (signλ_2_)ρ ≤ −0.02 a.u.

In Figure 7 the QTAIM analysis for some representative complexes is shown. As noticed, in the case of complexes **2** and **56** (Figure 7a—binding mode a), which represent the classical Watson-Crick BP disposition, three bond critical points (BCPs) and bond paths emerge, connecting both bases through HBs. Two of them connect (i) an O atom from a carbonyl group of FU and G to the amino group from A and ClC and (ii) the NH groups from FU and G to the N atoms from A and ClC, respectively. Also, in the case of complex **2**, a third BCP connects an O atom belonging to another carbonyl group from FU to a CH group belonging to A, thus characterizing an ancillary HB. The relative strength of these three HBs can be inspected by means of the NCIplot analysis, which resulted in a greenish isosurface between the O and CH groups, denoting a weaker HB compared to the other two, for which a bluish isosurface was obtained, hence denoting a strong interaction. On the other hand, in the case of complex **56** the third BCP connects the sp^2^ O atom from ClC to the amino group of G, thus characterizing another HB. In this case, all three HBs were colored by a bluish isosurface in the NCIplot analysis, thus anticipating a strong nonbonding interaction, as it is commonly known (see Figure 8a).

In the case of complexes **34** and **73** (Figure 7b—binding mode b, representing the Hoogsteen base pairing), two BCPs and bond paths characterized them. First, in complex **34**, two BCPs involved (i) a sp^2^ O atom from U and the amino group from BrA and (ii) a NH group from U and a sp^2^ N atom from BrA. On the other hand, in complex **73**, a double HB was characterized by the presence of two BCPs and bond paths which involved the amino group of C as HB donor moiety and a sp^2^ O and N atoms acting as HB acceptor entities. Interestingly, the NCIplot analysis revealed that in complex **34** both HBs equally participate in the stability of the complex, as indicated by the blue isosurface located between both molecules (see Figure 8b). However, the opposite was observed in the case of complex **73**, where the HB established between the NH_2_ group of C and the N atom of BrG exhibited a bluish isosurface (denoting a stronger NCI), while the other HB showed a greenish isosurface between both moieties, therefore accounting for a weaker interaction.

For complexes **37** and **90** (Figure 7c—binding mode c), three BCPs and bond paths were observed in complex **37**, connecting (i) an O atom from a carbonyl group of ClU to the amino group of ClA and (ii) the Cl atom from ClU to the amino group of ClA, thus describing a bifurcated HB. The third BCP connected the Cl atom from ClU to the sp^2^ N atom from ClA, therefore characterizing a halogen bonding interaction. On the other hand, in the case of complex **90** two HBs are denoted by the presence of two BCPs and bond paths connecting the NH_2_ and NH groups from BrC and BrG to a sp^2^ O atom from BrG and to the Br atom from BrC, respectively. After analyzing the NCIplot surfaces (Figure 8c), both HBs and the HalB contributed in a similar way to the stability of the BP in the case of complex **37**, however, in complex **90** the NH···O HB resulted in a stronger binding force compared to the Br···HN HB.

Finally, in Figure 7d the distribution of BCPs and bond paths for complexes **52** and **104** (binding mode d) is shown. As noted, in complex **52** three BCPs and bond paths dictate the stability of the BP, being two of them part of a bifurcated HB (connecting the O and I atoms from IU to the amino group of IA). The third BCP connects the I atom from the IU molecule to the sp^2^ N atom from IA, thus characterizing a HalB. Lastly, in the case of complex **104**, two BCPs are present, describing a HB (connecting the amino group from IC to the carbonyl group of IG) and a HalB (connecting the I and N atoms from IC and IG molecules, respectively). The NCIplot analysis (see Figure 8d) revealed that in the case of complex **52**, the contribution of the I···N HalB to the stability of the complex was higher than that of the bifurcated HB, as noticed by the bluish color of the HalB isosurface. In the case of complex **104**, both hydrogen bonding and halogen bonding interactions equally contributed to the stability of the complex, as denoted by the similar isosurface coloring of both NCIs as well as their respective BCP rho values.

### 2.5. Selected PDB Examples

**Figure 9:**
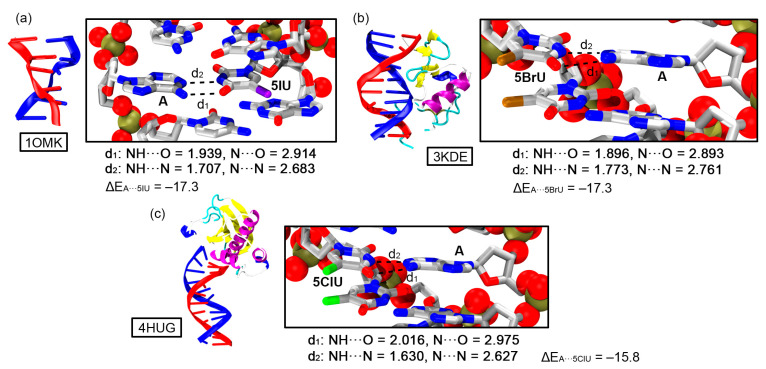
Partial views of X-ray structures exhibiting halogenated U···A BPs. The BP interaction is magnified inside the rectangular parts of the figure; 1OMK (**a**), 3KDE (**b**), and 4HUG (**c**). Distances are given in Å and energies in kcal/mol.

As the final stage of our study, we selected several representative examples from the PDB search and evaluated the stability of halogenated BPs (three of them are shown in Figure 9). The selected examples involved a distorted DNA structure (Figure 9a, PDBID: 1OMK) [41] corresponding to the first antiviral nucleoside 5-iodo-2’-deoxyuridine (IDU) against herpes simplex virus type 1 and type 2 and two protein-DNA complexes, which involve a THAP-family C(2)CH zinc-coordinating DNA-binding protein (Figure 9b, PDBID: 3KDE) [42] and the *Bacillus halodurans* RNase H (*Bh* RNase H) protein (Figure 9c, PDBID: 4HUG) [43].

All three structures exhibit the classical U···A binding mode involving the Watson-Crick face of A with two well defined NH···O and NH···N HBs. For each example a theoretical model involving the halogenated bases was built (see Supplementary Materials section for the cartesian coordinates) and the H atoms relaxed at a DFT level (BP86-D3/def2-SVP) prior to the evaluation of the interaction strength by means of single point calculations (RI-MP2/def2-TZVP level of theory). As noticed, the results are similar to those obtained from the computational study (see above) in terms of intermolecular distances (lying within the same range) and energetics, which are of similar magnitude. In addition, the energetic difference between the three selected halogenated BPs is almost negligible, also in line with the results obtained from the energetic study, thus giving reliability to the energies derived from fully optimized structures. Finally, in Table 5 the interaction energies and intermolecular distances of the rest of the selected PDB examples are shown.

## 3. Materials and Methods

The following computational techniques are in line with the state of the art theoretical methods available in the computational chemist toolbox. All energy calculations were carried out using the TURBOMOLE 7.2 software [44].

### 3.1. Calculations on Base Pair Complexes (Binding Modes a, b, c and d)

The binding energies were calculated following the supermolecule approximation, that is, as the energetic difference between the optimized geometries of the base pair and the isolated nucleobases (ΔE_complex_ = E_base pair_ − E_nucleobase 1_ − E_nucleobase 2_), at the RI-MP2 [45]/def2-TZVP [46] level of theory. In this regard, the RI-MP2 method combined with TZVP basis set was chosen since it achieved success to accurately represent noncovalent interaction energies involving both neutral and charged electron donors [47]. In regard to the RI basis set used [48], the uncontracted auxiliary basis set is optimized at the TZVPP level, the size being determined by the accuracy requirement for the element with the greatest MP2 correlation energy within a group of elements. For the TZVP basis set the SVP auxiliary basis is decontracted until the accuracy requirements are fulfilled. As the polarization functions are identical for SVP and TZVP, it is not necessary to add high *l*-quantum number auxiliary functions. The binding energies were calculated with correction for the basis set superposition error (BSSE) by using the Boys–Bernardi counterpoise technique [49]. The Cs symmetry point group was imposed during the optimization procedure.

### 3.2. Calculations on Selected X-ray Structures

Initially, the H atoms of the complex were optimized at the BP86 [50]-D3 [51]/def2-SVP [46] level of theory to obtain a more reliable position prior to the evaluation of the interaction energy. The interaction energies were computed using the supermolecule approximation at the RI-MP2/def2-TZVP level of theory by means of single point calculations of the complex and E_nucleobase 1_ and E_nucleobase 2_. In this case, to obtain the values of E_nucleobase 1_ and E_nucleobase 2_, their corresponding geometries were subtracted from the base pair complex, and their energies evaluated separately.

### 3.3. Molecular Electrostatic Potential Calculations (MEP)

The equations to compute the electrostatic potential values gathered in this manuscript can be found in the studies from Politzer and Murray [52,53]. Initially, the structure of the nucleobase was optimized at RI-MP2/def2-TZVP level of theory using the TURBOMOLE 7.2 software [44]. Once the wavefunction file was generated, it was treated with the Multiwfn program [54] using the medium quality setting to obtain the total density and electrostatic potential cube files. Finally, these cube files were treated with the Gaussview 6.0 [55] software where the electrostatic potential value can be obtained at any point of the surface. The electrostatic potential values (V, in kcal/mol) gathered in Table 1 and Table 2 were calculated using 0.001 and 0.002 a.u. isovalues. We have used two isovalues since atoms often interact at distances that are shorter than the sum of their vdW radii. Despite of this, it is also worth mentioning that the isovalue of 0.001 a.u. was used in the studies from Politzer and Murray [52,53] regarding the computation of the electrostatic potential values. On the other hand, in Bader’s study on atomic volumes [56] where both isovalue contours were used, the results were independent of the choice of contour value while they were enough to contain more than the 95% of the electronic charge and lie within the common limits of vdW contact distances.

### 3.4. QTAIM Analysis

The wavefunction analyses were performed at the RI-MP2/def2-TZVP level of theory using Multiwfn software. The topological properties of electron density were analysed using the QTAIM methodology [57]. The visualization of the QTAIM graphs has been carried out by means of the VMD software [58]. A brief description of some relevant concepts within Bader’s topology analysis is appropriate to facilitate the analysis of the results. The existence of a bond path connecting two nuclei implies that the two atoms are bonded to one another. Such a path is characterized by the bond critical point (BCP), which is the point exhibiting a minimum charge density along the bond, but a maximum along the directions perpendicular to the bond path. A critical point can be characterized by the number of zero eigenvalues of the associated Hessian matrix and the algebraic sum of their signs, which determine its rank and its signature, respectively. A bond critical point is denoted as (3, −1) and has one positive (λ_3_) and two negative (λ_1_, λ_2_) curvatures, one (λ_3_) associated with the charge density along the bond path and the other two (λ_1_, λ_2_) perpendicular to the bond path. There can be other types of nondegenerate critical points: (3, −3), (3, +1), and (3, +3). The first corresponds to position of local maxima of the charge density (the nuclei). The two other types occur as a consequence of particular geometrical arrangements of bond paths and define elements of molecular structure. If the bond paths are linked so as to form a ring of bonded atoms, a (3, +1) ring critical bond is formed in the interior of the ring. If the bond paths are arranged as to enclose the interior of a molecule with ring surfaces, then a (3, +3) cage critical point is found in the interior of the cage, the charge density being a local minimum at such a point. The characteristics of the bond critical point (BCP) were discussed in terms of the electron density (ρ) and it’s Laplacian (∇^2^ρ).

### 3.5. NCIplot Analysis

Finally, the NCIplot [59] index allows convenient visualization of both inter and intra molecular interactions in real space. It plots isosurfaces of the reduced-density gradient (related to |∇|/ρ^4/3^), which are coloured in agreement to values of the electron density. The NCI contacts are characterized by the regions of small reduced density gradient (RDG) at low densities, being mapped in real-space by plotting an isosurface of *s* for a low value of RDG. Additionally, the sign of the second eigenvalue of the density Hessian times the density, is color-mapped onto the isosurfaces, which allows the characterization of both the strength and (un)favorable nature of the interactions. More precisely, the colour scheme is composed by a red–yellow–green–blue scale using red for repulsive (ρ^+^_cut_) and blue for attractive (ρ^–^_cut_) NCI interaction density. Weak repulsive and weak attractive interactions are identified by yellow and green surfaces, respectively. The Density and RDG cutoffs used for the NCIplot calculation were 0.5 and 0.05, respectively. Regarding the cutplot values used, these were 0.5 for the Density and 0.05 for the RDG. The visualization of the NCIplot isosurfaces was carried out by means of the VMD software [58].

## 4. Concluding Remarks

In this study a series of halogenated A···U and G···C base pairs were computed, and their binding preferences disclosed at the RI-MP2/def2-TZVP level of theory. We observed a marked preference for the canonical (Watson-Crick base pairing) in C···G sets of complexes, while in the case of U···A complexes, both binding modes a and b (Hoogsteen base pairing) resulted in a similar strength of the BP. In addition, binding modes c and d (were both HB and HalB coexist as a source of BP stabilization) did not achieved the same stability in the case of U···A complexes, being around 6 kcal/mol weaker than their analogous of binding modes a and b. On the contrary, in the case of C···G complexes, these two binding modes achieved comparable energies to the results obtained from binding mode b. These findings were rationalized in terms of the electrostatic potential of the H and Hal atoms involved in the stability of the different binding modes as well as in the analysis of the wavefunction, which shed light into the physical nature and extension in real space of the interaction. Finally, several X-ray selected examples derived from the search were also calculated, obtaining results that agreed with those retrieved from the theoretical study. We expect that the results from this study will be of interest for the chemical biology and supramolecular chemistry communities as well as for advancements in the understanding of the halogenation effects on nucleobase stability.

## Figures and Tables

**Table 1:** Molecular Electrostatic Potential values of the halogen bond and hydrogen bond donor groups (V_Hal_, V_CH_, V_NH_, V_NH2-1_, and V_NH2-2_, in kcal/mol). These were calculated over the Halogen and H atoms belonging to the C–Hal, C–H and N–H bonds (see the white asterisks in Figure 3 to locate the specific position where the MEP was computed) involving non-halogenated and halogenated Uracil and Cytosine nucleobases at 0.001 a.u. Values in parenthesis correspond to an isovalue contour of 0.002 a.u. (also in kcal/mol). The MEP maxima (V_max_) and minima (V_min_) values for each nucleobase are also indicated in kcal/mol.

**U**	**V_max_** = +55.2 (+68.4)	**V_min_** = −34.5 (−38.3)
	**V_CH_** = +22.0 (+30.1)	**V_NH_** = +37.7 (+51.2)
**C**	**V_max_** = +52.1 (+64.6)	**V_min_** = −54.6 (−60.2)
	**V_CH_** = +30.7 (+37.0)	**V_NH2-1_** = +51.5 (+64.0)/V_NH2-2_= +40.7 (+53.3)
**HalU**		
**FU**	**V_max_** = +57.7 (+72.2)	**V_min_** = −33.8 (−35.8)
	**V_Hal_** = −8.8 (−7.5)	**V_NH_** = +42.0 (+57.1)
**ClU**	**V_max_** = +57.7 (+72.1)	**V_min_** = −33.8 (−37.7)
	**V_Hal_** = +11.3 (+16.9)	**V_NH_** = +41.4 (+56.5)
**BrU**	**V_max_** = +56.5 (+71.5)	**V_min_** = −31.4 (−37.7)
	**V_Hal_** = +16.9 (+25.1)	**V_NH_** = +41.4 (+55.8)
**IU**	**V_max_** = +56.5 (+71.5)	**V_min_** = −33.3 (−37.0)
	**V_Hal_** = +23.2 (+32.0)	**V_NH_** = +40.8 (+55.2)
**HalC**		
**FC**	**V_max_** = +46.4 (+61.5)	**V_min_** = −50.2 (−55.8)
	**V_Hal_** = −0.2 (+0.52)	**V_NH2-1_** = +46.4 (+59.6)/V_NH2-2_ = +39.5 (+53.3)
**ClC**	**V_max_** = +49.6 (+62.1)	**V_min_** = −49.7 (−55.2)
	**V_Hal_** = +16.9 (+23.2)	**V_NH2-1_** = +42.7 (+57.1)/V_NH2-2_ = +39.5 (+52.7)
**BrC**	**V_max_** = +46.4 (+60.2)	**V_min_** = −50.8 (−56.5)
	**V_Hal_** = +23.2 (+30.1)	**V_NH2-1_** = +42.0 (+57.7)/V_NH2-2_ = +42.0 (+57.1)
**IC**	**V_max_** = +46.4 (+61.5)	**V_min_** = −49.6 (−55.2)
	**V_Hal_** = +28.2 (+37.0)	**V_NH2-1_** = +37.6 (+54.6)/V_NH2-2_ = +37.7 (+51.5)

**Table 2:** Molecular electrostatic potential values of the halogen bond and hydrogen bond donor groups (V_Hal_, V_CH-1_, V_CH-2_, V_NH_ and V_NH2_, in kcal/mol). These were calculated over the Halogen and H atoms belonging to the C–Hal, C–H and N–H bonds (see the white asterisks in Figure 3 to locate the specific position where the MEP was computed) involving non-halogenated and halogenated Adenine and Guanine nucleobases at 0.001 a.u. Values in parenthesis correspond to an isovalue contour of 0.002 a.u. (also in kcal/mol). The MEP maxima (V_max_) and minima (V_min_) values for each nucleobase are also indicated in kcal/mol.

**A**	**V_max_** = +53.0 (+67.1)	**V_min_** = −31.7 (−37.6)	
	**V_CH-1_** = +28.9 (+36.6)	**V_CH-2_** = +7.5 (+14.2)	**V_NH2_** = +36.4 (+47.9)
**G**	**V_max_** = +63.0 (+74.0)	**V_min_** = −54.2 (−58.3)	
	**V_CH_** = +23.2 (+31.3)	**V_NH2_** = +59.6 (+73.7)	**V_NH_** = +49.6 (+58.3)
**HalA**			
**FA**	**V_max_** = +56.3 (+71.8)	**V_min_** = −28.9 (−34.7)	
	**V_Hal_** = −1.3 (+1.5)	**V_CH-2_** = +10.7 (+16.8)	**V_NH2_** = +37.7 (+45.2)
**ClA**	**V_max_** = +54.2 (+69.5)	**V_min_** = −29.2 (−35.1)	
	**V_Hal_** = +16.9 (+23.2)	**V_CH-2_** = +10.6 (+16.8)	**V_NH2_** = +38.9 (+48.9)
**BrA**	**V_max_** = +53.4 (+68.8)	**V_min_** = −29.3 (−35.8)	
	**V_Hal_** = +22.0 (+30.1)	**V_CH-2_** = +10.0 (+16.8)	**V_NH2_** = +38.3 (+46.4)
**IA**	**V_max_** = +52.4 (+67.1)	**V_min_** = −29.4 (−35.5)	
	**V_Hal_** = +27.6 (+37.0)	**V_CH-2_** = +10.0 (+16.6)	**V_NH2_** = +37.6 (+48.9)
**HalG**			
**FG**	**V_max_** = +62.5 (+73.2)	**V_min_** = −49.0 (−53.4)	
	**V_Hal_** = −6.3 (−5.6)	**V_NH2_** = +58.9 (+72.9)	**V_NH_** = +48.3 (+61.5)
**ClG**	**V_max_** = +62.8 (+73.6)	**V_min_** = −49.1 (−53.3)	
	**V_Hal_** = +11.9 (+18.2)	**V_NH2_** = +59.6 (+73.4)	**V_NH_** = +49.6 (+62.1)
**BrG**	**V_max_** = + 63.3 (+73.8)	**V_min_** = −48.9 (−53.1)	
	**V_Hal_** = +17.6 (+25.1)	**V_NH2_** = +59.6 (+73.5)	**V_NH_** = +50.2 (+61.9)
**IG**	**V_max_** =+62.6 (+73.1)	**V_min_** = −49.3 (−53.3)	
	**V_Hal_** = +23.2 (+32.3)	**V_NH2_** = +59.0 (+ 73.6)	**V_NH_** = +50.2 (+62.7)

**Table 3:** BSSE corrected interaction energies (ΔE_BSSE_, in kcal/mol), equilibrium distances (d_1_, d_2_ and d_3_ in Å) and value of the density at the bond critical point corresponding to the strongest interaction (ρ·10^2^ in a.u.) for the U···A complexes **1** to **52**.

Complex	ΔE_BSSE_	d_1_	d_2_	ρ·10^2^
**Binding mode a**				
**1 (U···A)**	−13.6	1.932	1.760	4.73
**2 (FU···A)**	−14.0	1.933	1.751	4.85
**3 (ClU···A)**	−14.1	1.936	1.755	4.81
**4 (BrU···A)**	−14.1	1.927	1.748	4.88
**5 (IU···A)**	−14.1	1.922	1.742	4.95
**6 (U···FA)**	−13.6	1.913	1.786	4.44
**7 (U···ClA)**	−13.6	1.926	1.783	4.48
**8 (U···BrA)**	−13.6	1.911	1.757	4.76
**9 (U···IA)**	−13.7	1.909	1.784	4.46
**10 (FU···FA)**	−13.9	1.935	1.759	4.75
**11 (ClU···ClA)**	−14.0	1.927	1.762	4.71
**12 (Br···BrA)**	−14.0	1.923	1.761	4.73
**13 (IU···IA)**	−14.1	1.924	1.760	4.74
**Binding mode b**				
**14 (U···A)**	−14.4	1.970	1.740	4.81
**15 (FU···A)**	−14.8	1.977	1.733	4.91
**16 (ClU···A)**	−14.8	1.972	1.735	4.88
**17 (BrU···A)**	−14.8	1.966	1.729	4.96
**18 (IU···A)**	−14.8	1.973	1.735	4.88
**19 (U···FA)**	−11.4	1.879	1.939	2.94
**20 (U···ClA)**	−11.6	1.854	2.030	2.40
**21 (U···BrA)**	−11.4	1.847	2.079	2.15
**22 (U···IA)**	−11.2	1.854	2.118	1.98
**23 (FU···FA)**	−11.5	1.895	1.921	3.07
**24 (ClU···ClA)**	−11.7	1.860	2.019	2.47
**25 (BrU···BrA)**	−11.5	1.853	2.071	2.20
**26 (IU···IA)**	−11.3	1.851	2.115	2.00
**Binding mode c**				
**27 (U···A)**	−8.3	1.938	2.313	2.44
**28 (FU···A)**	−4.2	2.118	3.537	1.58
**29 (ClU···A)**	−5.5	2.163	3.231	1.43
**30 (BrU···A)**	−5.8	2.246	3.182	1.18
**31 (IU···A)**	−5.9	2.363	3.203	1.41
**32 (U···FA)**	−8.3	1.929	2.327	2.49
**33 (U···ClA)**	−8.4	1.925	2.328	2.51
**34 (U···BrA)**	−8.4	1.924	2.329	2.52
**35 (U···IA)**	−8.4	1.925	2.326	2.51
**36 (FU···FA)**	−4.4	2.073	3.525	1.74
**37 (ClU···ClA)**	−5.7	2.150	3.215	1.47
**38 (BrU···BrA)**	−6.0	2.231	3.197	1.22
**39 (IU···IA)**	−6.1	2.346	3.228	1.35
**Binding mode d**				
**40 (U···A)**	−7.4	1.972	2.318	2.22
**41 (FU···A)**	−3.7	2.302	3.459	1.16
**42 (ClU···A)**	−5.5	2.328	3.120	1.10
**43 (BrU···A)**	−6.1	2.403	3.037	1.42
**44 (IU···A)**	−6.7	2.527	3.017	1.84
**45 (U···FA)**	−7.9	1.964	2.331	2.24
**46 (U···ClA)**	−8.3	1.961	2.294	2.26
**47 (U···BrA)**	−8.4	1.962	2.282	2.26
**48 (U···IA)**	−8.6	1.965	2.261	2.24
**49 (FU···FA)**	−4.1	2.241	3.410	1.31
**50 (ClU···ClA)**	−6.0	2.307	3.078	1.14
**51 (BrU···BrA)**	−4.5	2.379	3.034	1.42
**52 (IU···IA)**	−7.4	2.477	3.024	1.82

**Table 4:** BSSE corrected interaction energies (ΔE_BSSE_, in kcal/mol), equilibrium distances (d_1_, d_2_ and d_3_ in Å), and value of the density at the bond critical point corresponding to the strongest interaction (ρ·10^2^ in a.u.) for the C···G complexes **53** to **104**.

Complex	ΔE_BSSE_	d_1_	d_2_	d_3_	ρ·10^2^
**Binding mode a**					
**53 (C···G)**	−29.3	1.726	1.881	1.885	3.52
**54 (FC···G)**	−28.2	1.720	1.883	1.891	4.23
**55 (ClC···G)**	−28.0	1.712	1.894	1.883	4.32
**56 (BrC···G)**	−28.8	1.713	1.892	1.887	4.32
**57 (IC···G)**	−28.0	1.712	1.895	1.884	4.34
**58 (C···FG)**	−28.6	1.727	1.878	1.882	4.17
**59 (C···ClG)**	−28.6	1.734	1.878	1.876	4.10
**60 (C···BrG)**	−28.6	1.732	1.878	1.881	4.12
**61 (C···IG)**	−28.5	1.736	1.878	1.878	4.08
**62 (FC···FG)**	−27.4	1.727	1.882	1.888	4.16
**63 (ClC···ClG)**	−27.2	1.719	1.886	1.885	4.26
**64 (BrC···BrG)**	−28.0	1.719	1.889	1.881	4.26
**65 (IC···IG)**	−27.1	1.718	1.891	1.879	4.27
**Binding mode b**					
**Complex**	**ΔE_BSSE_**	**d_1_**	**d_2_**	**ρ·10^2^**	
**66 (C···G)**	−15.4	3.746	1.922	1.46	
**67 (FC···G)**	−12.3	2.119	2.173	1.79	
**68 (ClC···G)**	−12.3	2.092	2.195	1.76	
**69 (BrC···G)**	−13.4	2.625	1.951	2.81	
**70 (IC···G)**	−15.3	3.676	1.896	3.26	
**71 (C···FG)**	−10.9	2.005	2.924	2.12	
**72 (C···ClG)**	−10.2	2.837	2.005	2.59	
**73 (C···BrG)**	−10.4	2.646	1.981	2.60	
**74 (C···IG)**	−11.5	3.269	1.951	2.80	
**75 (FC···FG)**	−9.9	2.225	2.126	1.63	
**76 (ClC···ClG)**	−10.2	2.233	2.115	1.66	
**77 (BrC···BrG)**	−11.1	2.633	1.970	2.66	
**78 (IC···IG)**	−11.4	3.241	1.935	2.92	
**Binding mode c**					
**79 (C···G)**	−12.9	1.986	2.634	2.21	
**80 (FC···G)**	−10.6	1.890	2.043	2.64	
**81 (ClC···G)**	−8.9	1.971	2.941	2.31	
**82 (BrC···G)**	−9.6	1.957	2.772	2.24	
**83 (IC···G)**	−8.6	1.972	2.941	2.18	
**84 (C···FG)**	−10.9	2.769	2.881	2.12	
**85 (C···ClG)**	−11.0	2.769	2.963	2.11	
**86 (C···BrG)**	−11.0	2.771	2.991	2.11	
**87 (C···IG)**	−11.2	2.733	3.105	2.12	
**88 (FC···FG)**	−9.2	1.895	2.043	2.61	
**89 (ClC···ClG)**	−7.4	1.948	2.614	2.28	
**90 (BrC···BrG)**	−8.1	1.962	2.771	2.22	
**91 (IC···IG)**	−7.1	1.975	2.939	2.16	
**Binding mode d**					
**92 (C···G)**	−12.7	1.978	2.472	2.06	
**93 (FC···G)**	−8.8	2.027	3.366	1.91	
**94 (ClC···G)**	−9.9	2.114	3.121	1.92	
**95 (BrC···G)**	−11.2	2.047	3.128	1.70	
**96 (IC···G)**	−10.8	2.114	3.121	1.49	
**97 (C···FG)**	−11.0	1.987	2.488	1.99	
**98 (C···ClG)**	−11.1	2.003	2.438	1.95	
**99 (C···BrG)**	−11.2	2.011	2.421	1.93	
**100 (C···IG)**	−11.4	2.024	2.388	1.88	
**101 (FC···FG)**	−7.2	1.992	3.128	2.01	
**102 (ClC···ClG)**	−8.4	2.025	3.147	1.82	
**103 (BrC···BrG)**	−9.7	2.048	3.127	1.69	
**104 (IC···IG)**	−9.5	2.114	3.121	1.48	

**Table 5:** List of selected PDB structures (PDBID), halogenated base pair combination (BP), BSSE corrected interaction energies (ΔE_BSSE_, in kcal/mol) and intermolecular distances (d_1_, d_2_ and d_3_, in Å).

PDBID	BP	ΔE_BSSE_	d_1_	d_2_	d_3_
**1IJW**	BrC···G	−25.5	1.927	1.769	1.602
**3BSU**	IU···A	−11.7	1.936	1.638	-
**3JXR**	FC···G	−23.1	2.395	2.204	1.797
**3KDE**	BrU···A	−17.3	1.853	1.655	-
**4HUG**	ClU···A	−15.8	1.947	1.505	-
**4XSN**	C···BrG	−29.5	1.831	1.929	1.837
**5AY3**	BrC···G	−27.0	1.816	1.828	1.798
**7EDT**	BrU···A	−13.9	1.880	1.663	-
**1OMK**	IU···A	−17.3	1.866	1.562	-

## Data Availability

All data needed to reproduce this work can be found in the Supplementary Materials file.

## Supplementary Materials

The following supporting information can be downloaded at: https://www.mdpi.com/article/10.3390/ijms24065530/s1. List of PDB structures, additional AIM analyses, cartesian coordinates of complexes **1** to **102** and of the selected PDB examples.
