# Supplementary material for: A Comprehensive Ab Initio Study of Halogenated A···U and G···C Base Pair Geometries and Energies
Source: Int J Mol Sci. 2023 Mar 14;24(6):5530. doi: 10.3390/ijms24065530 (PMC10056977; doi:10.3390/ijms24065530)
Supplement: Supplementary file 1 [file ijms-24-05530-s001.zip › ijms-2249334-supplementary.pdf]

A comprehensive *ab initio* study of halogenated A···U and G···C base pair geometries and energies

Rosa M. Gomila[a], Antonio Frontera[a] and Antonio Bauzá*,[a]

[a]Department of Chemistry, Universitat de les Illes Balears, Crta. de Valldemossa km 7.5, 07122 Palma (Islas Baleares), SPAIN.
Fax: (+) 34 971 173426; E-mail: antonio.bauza@uib.es

Electronic Supplementary Information

Index

List of PDB structures page 2
Additional AIM analyses page 3
Cartesian coordinates of complexes 1 to 102 page 19
Cartesian coordinates of the selected PDB examples page 65

List of PDB structures

PDB structures belonging to bound nucleic acid structures:

3KDE, 3IL2, 1WTP, 4E10, 5A3D, 1JJ8, 1JJ6, 1BF4, 2H1O, 2IRF, 1CKT, 2FCC, 3OH6, 3OGD, 3OH9, 1K3X, 7K30, 7K32, 4NOD, 3TQ6, 3BS1, 1AWC, 3VWB, 1AWC, 4HTU, 4HUE, 4HUG, 4HUF, 6UKF, 6UKE, 3BSU, 1P59, 2NLL, 1SRS, 1LEI, 1GA5, 1K61, 1AIS, 1ZME, 2RAM, 1A6Y, 1NH2, 2BSQ, 1FYL, 1DP7, 7R9F, 1LWW, 1IJW, 2Q2K.

PDB structures belonging to unbound nucleic acid structures:

5V1K, 7EDT, 2R20, 2R1S, 2R21, 1KFO, 4P3T, 3TD0, 1ZCI, 1JZV, 7BPG, 7BPF, 7LO9, 3BNS, 3BNR, 3MEI, 3BNQ, 2OEU, 2FCX, 5VJB, 3P59, 3ZD5, 1Y3S, 1Y90, 3BNT, 2PN4, 3BNO, 1YXP, 5UX3, 1Y3O, 2PN3, 2JLT, 4P3U, 5XWG, 3LOA, 1KH6, 1OMK, 464D, 5AY3, 5D8T, 5AY2, 4L26, 1DN5, 1J9H, 6CY2, 6HC5, 1IH6, 2G91, 6LBW, 5LQT, 6HBX, 1DN4, 5NEP, 242D, 5LR3, 6HBT, 5NEO, 5NEQ, 5AY4, 1UHY, 1LNT, 6YL5, 5NEX, 421D, 1V3N, 5V2H, 5LR4, 5NDH, 6CY4, 5LQO, 5NWQ, 5NEF, 5NOM, 318D, 238D, 1UHX, 5T5A, 5NZD, 6YMK, 5NY8, 6YMJ, 5NZ3, 376D, 430D, 6TF0, 5O62, 6YLB, 6YML, 319D, 6YMM, 1F6J, 4BNA, 458D, 5O69, 6CY0, 5LR5, 6FZ0, 6YMI, 5NDI, 2GOT, 7ECK, 2PIS, 1IH2, 5NZ6, 6TFH, 3BNA, 7ECL, 7ECJ, 6R47, 2FZA, 1MDG, 1N7A, 2AWE, 4XSN, 3R1E, 6JKN, 3R1D, 3IBK, 6M4T, 6WCK, 2XC6, 6IYQ, 6JJE, 6ROR, 2WNA, 2GRB, 4MGM, 3JXQ, 3JXR, 1DNF, 2AO5, 1UE2, 1V3O, 5UZA, 1V3P, 1JZV, 1ANA.

Additional AIM analyses

U···A – binding mode a

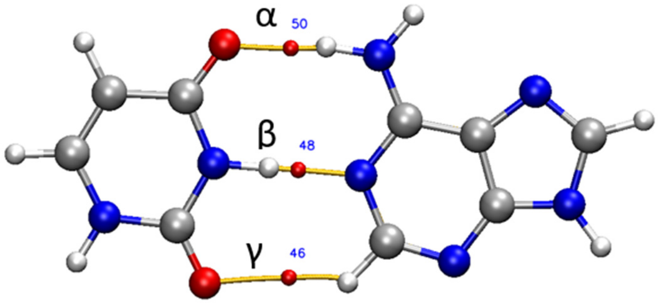

Table S1.

Complex	ρ·10^2 (α)	ρ·10^2 (β)	ρ·10^2 (γ)
**1 (U···A)**	2.50	4.73	0.57

5HalU···A – binding mode a

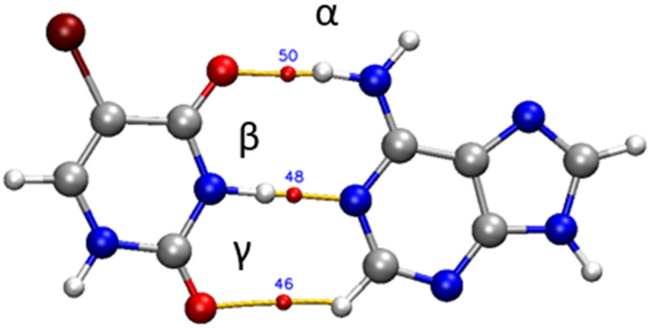

Table S2.

Complex	ρ·10^2 (α)	ρ·10^2 (β)	ρ·10^2 (γ)
**2 (FU···A)**	2.48	4.85	0.57
**3 (ClU···A)**	2.46	4.81	0.57
**4 (BrU···A)**	2.51	4.88	0.58
**5 (IU···A)**	2.55	4.95	0.58

U···8HalA – binding mode a

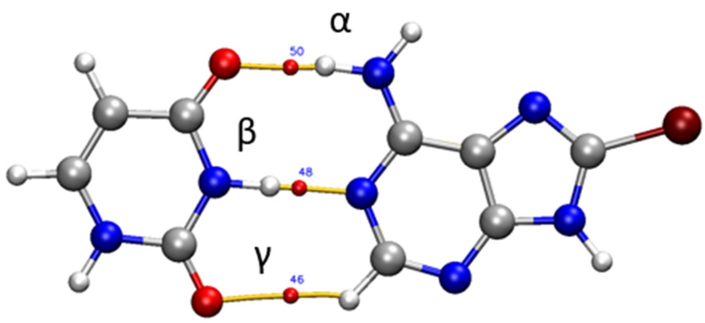

Table S3.

Complex	ρ·10^2 (α)	ρ·10^2 (β)	ρ·10^2 (γ)
**6 (U···FA)**	2.62	4.44	0.50
**7 (U···ClA)**	2.54	4.48	0.53
**8 (U···BrA)**	2.64	4.76	0.58
**9 (U···IA)**	2.65	4.46	0.50

5HalU···8HalA – binding mode a

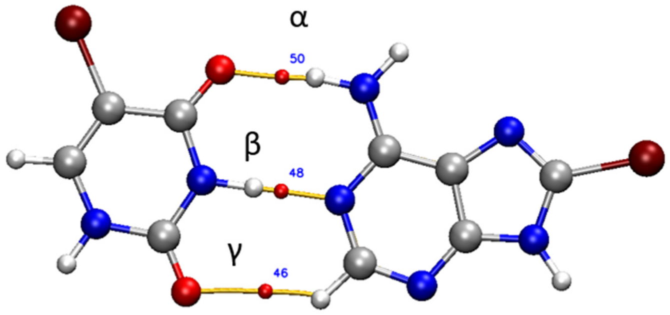

Table S4.

Complex	ρ·10^2 (α)	ρ·10^2 (β)	ρ·10^2 (γ)
**10 (FU···FA)**	2.47	4.75	0.57
**11 (ClU···ClA)**	2.52	4.71	0.56
**12 (BrU···BrA)**	2.54	4.73	0.56
**13 (IU···IA)**	2.54	4.74	0.56

U···A – binding mode b

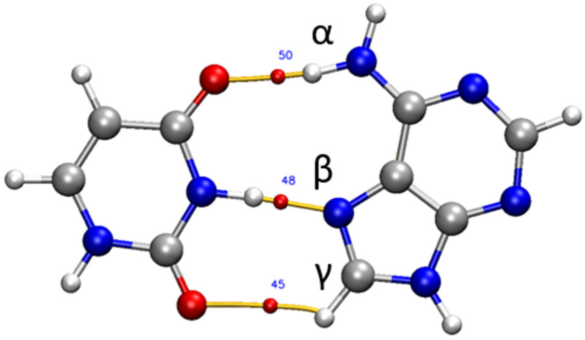

Table S5.

Complex	ρ·10^2 (α)	ρ·10^2 (β)	ρ·10^2 (γ)
**14 (U···A)**	2.22	4.81	0.61

5HalU···A – binding mode b

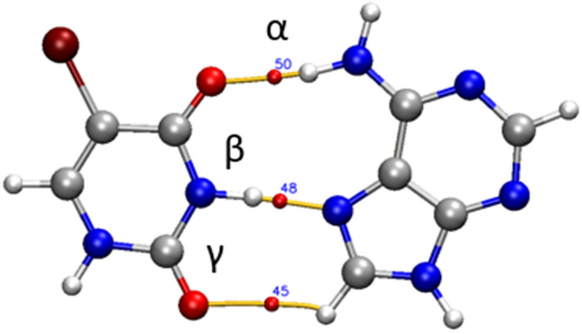

Table S6.

Complex	ρ·10^2 (α)	ρ·10^2 (β)	ρ·10^2 (γ)
**15 (FU···A)**	2.18	4.91	0.62
**16 (ClU···A)**	2.19	4.88	0.61
**17 (BrU···A)**	2.22	4.96	0.62
**18 (IU···A)**	2.19	4.88	0.62

U···5HalA – binding mode b

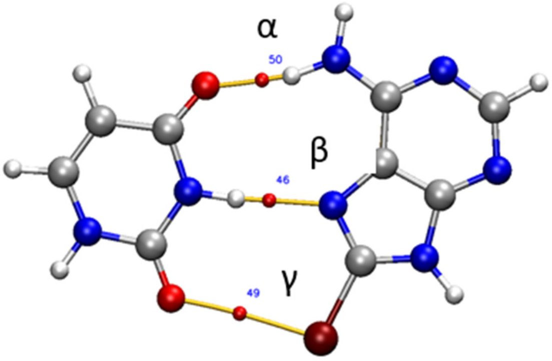

Table S7.

Complex	ρ·10^2 (α)	ρ·10^2 (β)	ρ·10^2 (γ)
**19 (U···FA)**	2.75	2.94	0.44
**20 (U···ClA)**	2.90	2.40	0.76
**21 (U···BrA)**	2.95	2.15	0.81
**22 (U···IA)**	2.89	1.98	0.92

5HalU···8HalA – binding mode b

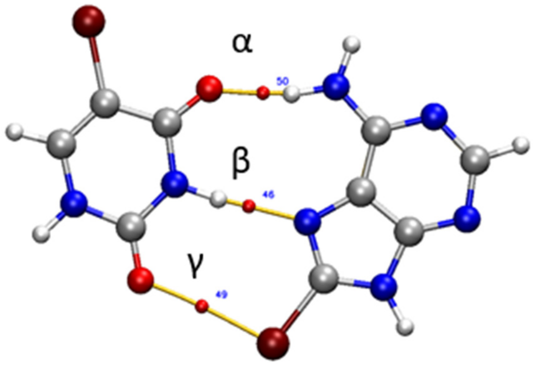

Table S8.

Complex	ρ·10^2 (α)	ρ·10^2 (β)	ρ·10^2 (γ)
**23 (FU···FA)**	2.63	3.07	0.48
**24 (ClU···ClA)**	2.83	2.47	0.62
**25 (BrU···BrA)**	2.87	2.20	0.84
**26 (IU···IA)**	2.90	2.00	0.93

U···A – binding mode c

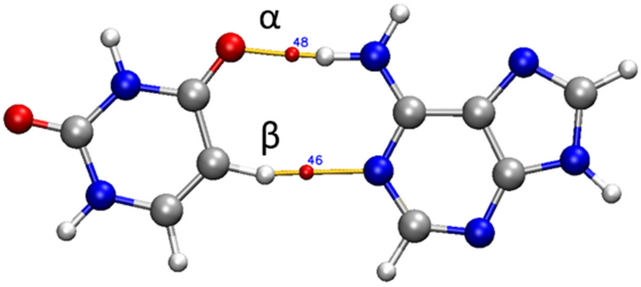

Table S9.

Complex	ρ·10^2 (α)	ρ·10^2 (β)
**27 (U···A)**	2.44	1.47

5HalU···A – binding mode c

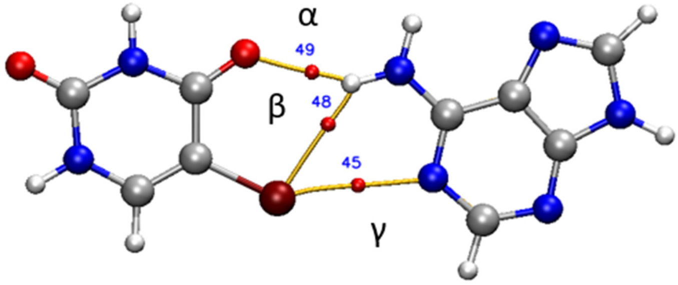

Table S10.

Complex	ρ·10^2 (α)	ρ·10^2 (β)	ρ·10^2 (γ)
**28 (FU···A)**	1.58	-	0.24
**29 (ClU···A)**	1.43	0.66	0.87
**30 (BrU···A)**	1.18	0.83	1.16
**31 (IU···A)**	0.91	0.97	1.41

U···8HalA – binding mode c

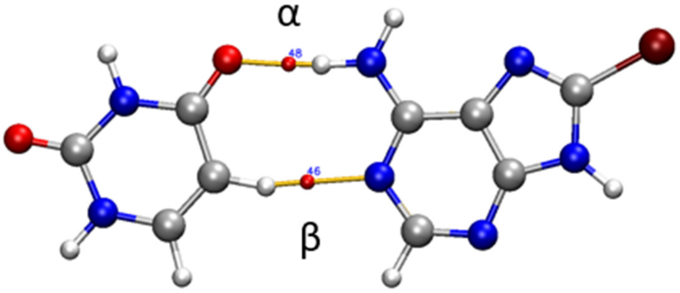

Table S11.

Complex	ρ·10^2 (α)	ρ·10^2 (β)
**32 (U···FA)**	2.49	1.43
**33 (U···ClA)**	2.51	1.43
**34 (U···BrA)**	2.52	1.42
**35 (U···IA)**	2.51	1.43

5HalU···8HalA – binding mode c

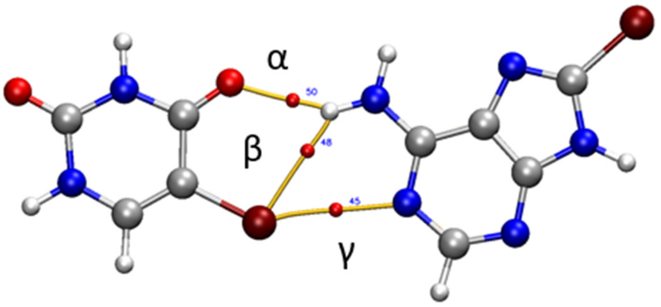

Table S12.

Complex	ρ·10^2 (α)	ρ·10^2 (β)	ρ·10^2 (γ)
**36 (FU···FA)**	1.74	-	0.25
**37 (ClU···ClA)**	1.47	0.65	0.89
**38 (BrU···BrA)**	1.22	0.80	1.13
**39 (IU···IA)**	0.95	0.94	1.35

U···A – binding mode d

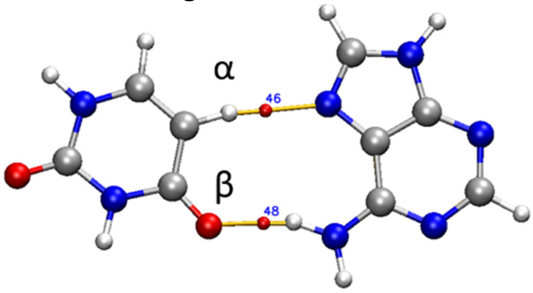

Table S13.

Complex	ρ·10^2 (α)	ρ·10^2 (β)
**40 (U···A)**	1.41	2.22

5HalU···A – binding mode d

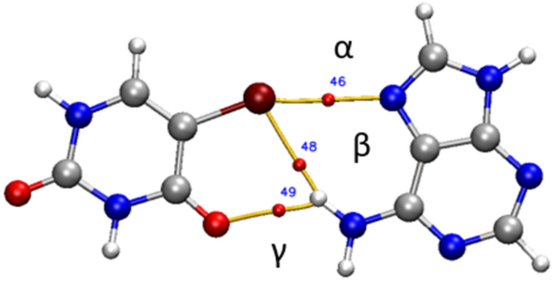

Table S14.

Complex	ρ·10^2 (α)	ρ·10^2 (β)	ρ·10^2 (γ)
**41 (FU···A)**	0.26	-	1.16
**42 (ClU···A)**	0.99	0.78	1.10
**43 (BrU···A)**	1.42	1.02	0.93
**44 (IU···A)**	1.84	1.26	0.72

U···8HalA – binding mode d

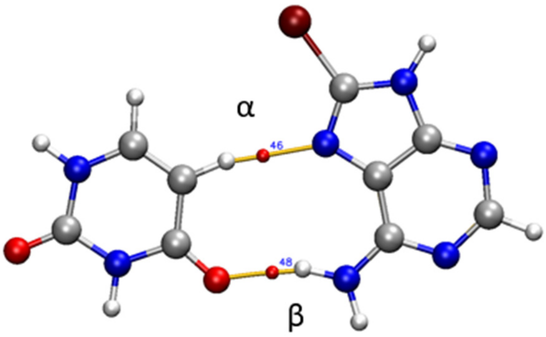

Table S15.

Complex	ρ·10^2 (α)	ρ·10^2 (β)	ρ·10^2 (γ)
**45 (U···FA)**	1.34	2.24	-
**45 (U···ClA)**	1.45	2.26	-
**47 (U···BrA)**	1.49	2.26	-
**48 (U···IA)**	1.57	2.24	0.17

5HalU···8HalA – binding mode d

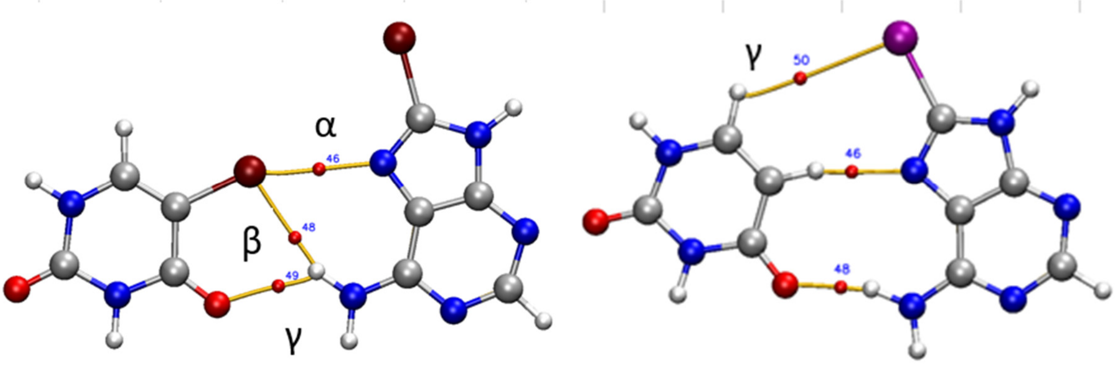

Table S16.

Complex	ρ·10^2 (α)	ρ·10^2 (β)	ρ·10^2 (γ)
**49 (FU···FA)**	0.27	-	1.31
**50 (ClU···ClA)**	1.07	0.75	1.14
**51 (BrU···BrA)**	1.42	0.95	0.97
**52 (IU···IA)**	1.82	1.16	0.78

C···G – binding mode a

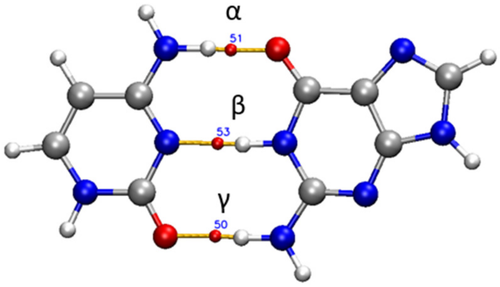

Table S17.

Complex	ρ·10^2 (α)	ρ·10^2 (β)	ρ·10^2 (γ)
**53 (C···G)**	4.18	3.52	2.82

5HalC···G – binding mode a

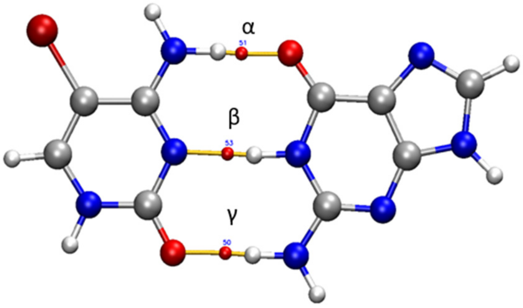

Table S18.

Complex	ρ·10^2 (α)	ρ·10^2 (β)	ρ·10^2 (γ)
**54 (FC···G)**	4.23	3.49	2.77
**55 (ClC···G)**	4.32	3.45	2.78
**56 (BrC···G)**	4.32	3.42	2.80
**57 (IC···G)**	4.34	3.40	2.82

C···8HalG – binding mode a

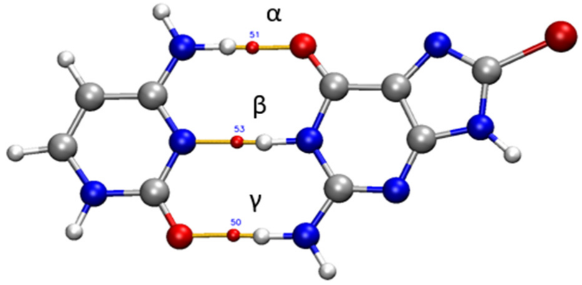

Table S19.

Complex	ρ·10^2 (α)	ρ·10^2 (β)	ρ·10^2 (γ)
**58 (C···FG)**	4.17	3.56	2.84
**59 (C···ClG)**	4.10	3.55	2.87
**60 (C···BrG)**	4.12	3.55	2.85
**61 (C···IG)**	4.08	3.55	2.86

5HalC···8HalG – binding mode a

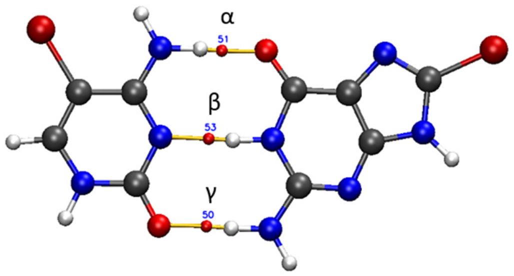

Table S20.

Complex	ρ·10^2 (α)	ρ·10^2 (β)	ρ·10^2 (γ)
**62 (FC···FG)**	4.16	3.50	2.79
**63 (ClC···ClG)**	4.26	3.47	2.81
**64 (BrC···BrG)**	4.26	3.45	2.84
**65 (IC···IG)**	4.27	3.44	2.85

C···G – binding mode b

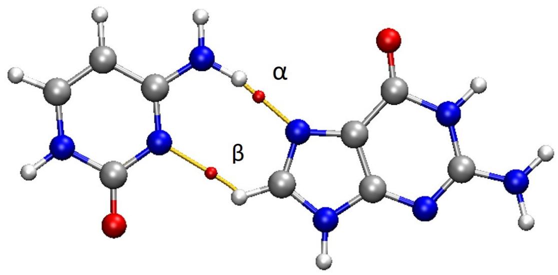

Table S21.

Complex	ρ·10^{2} (α)	ρ·10^{2} (β)
**66 (C···G)**	3.05	1.46

5HalC···G – binding mode b

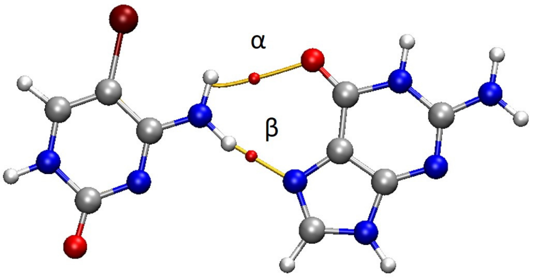

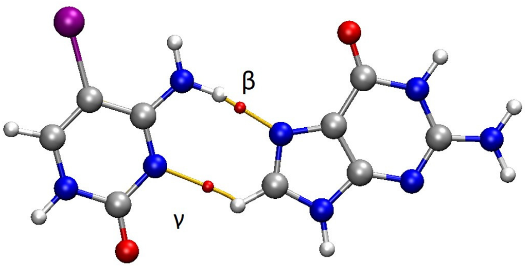

Table S22.

Complex	ρ·10^{2} (α)	ρ·10^{2} (β)	ρ·10^{2} (γ)
**67 (FC···G)**	1.67	1.79	-
**68 (ClC···G)**	1.76	1.72	-
**69 (BrC···G)**	0.65	2.81	-
**70 (IC···G)**	-	3.26	1.39

C···8HalG – binding mode b

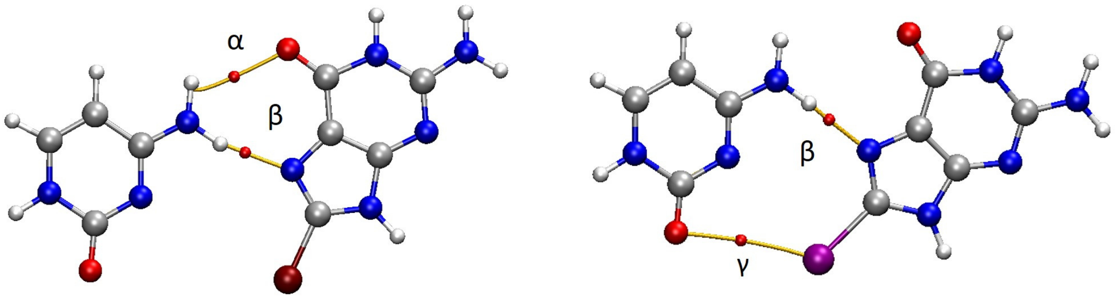

Table S23.

Complex	ρ·10^2 (α)	ρ·10^2 (β)	ρ·10^2 (γ)
**71 (FC···G)**	2.12	-	-
**72 (ClC···G)**	0.63	2.60	0.13
**73 (BrC···G)**	0.62	2.60	-
**74 (IC···G)**	-	2.80	0.52

8HalC···8HalG – binding mode b

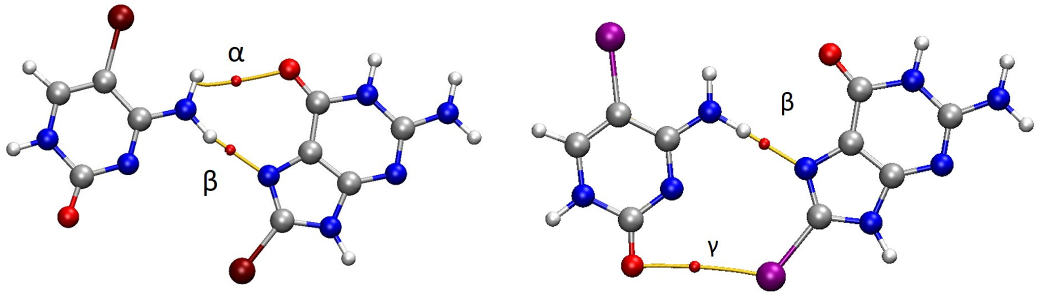

Table S24.

Complex	ρ·10^2 (α)	ρ·10^2 (β)	ρ·10^2 (γ)
**75 (FC···G)**	1.63	1.56	-
**76 (ClC···G)**	1.66	1.57	-
**77 (BrC···G)**	0.64	2.66	-
**78 (IC···G)**	-	2.92	0.53

C···G – binding mode c

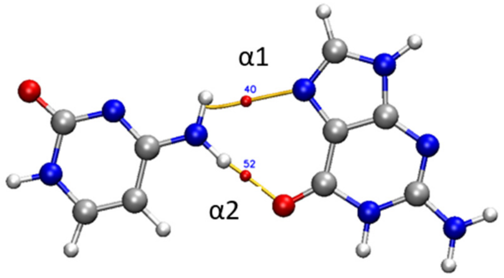

Table S25.

Complex	ρ·10^2 (α1)	ρ·10^2 (α2)
**79 (C···G)**	0.84	2.21

5HalC···G – binding mode c

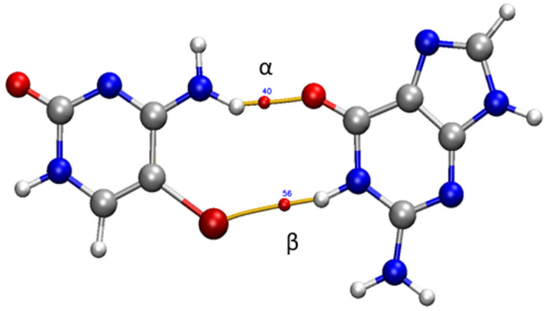

Table S26.

Complex	ρ·10^2 (α)	ρ·10^2 (β)
**80 (FC···G)**	2.64	1.38
**81 (ClC···G)**	2.31	0.86
**82 (BrC···G)**	2.24	0.82
**83 (IC···G)**	2.18	0.83

C···8HalG – binding mode c

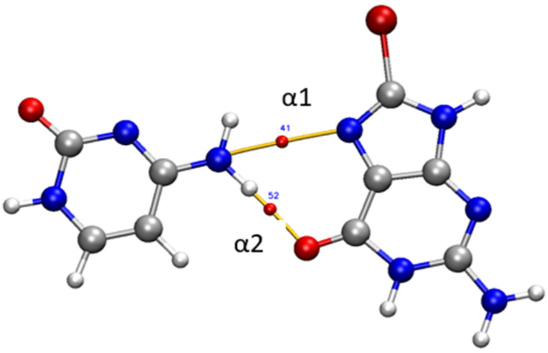

Table S27.

Complex	ρ·10² (α1)	ρ·10² (α2)
**84 (FC···G)**	0.60	2.12
**85 (ClC···G)**	0.64	2.11
**86 (BrC···G)**	0.66	2.11
**87 (IC···G)**	0.74	2.12

5HalC···8HalG – binding mode c

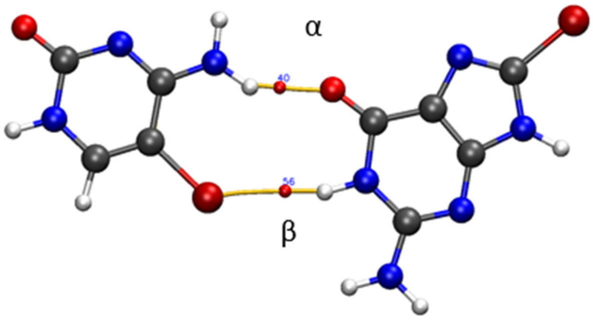

Table S28.

Complex	ρ·10² (α)	ρ·10² (β)
**88 (FC···FG)**	2.61	1.37
**89 (ClC···ClG)**	2.28	0.86
**90 (BrC···BrG)**	2.22	0.83
**91 (IC···IG)**	2.16	0.83

C···G – binding mode d

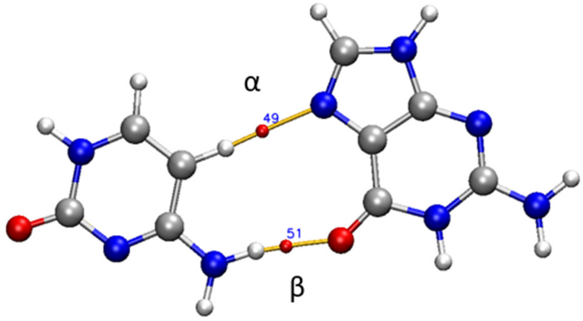

Table S29.

Complex	ρ·10² (α)	ρ·10² (β)
**92 (C···G)**	1.01	2.06

5HalC···G – binding mode d

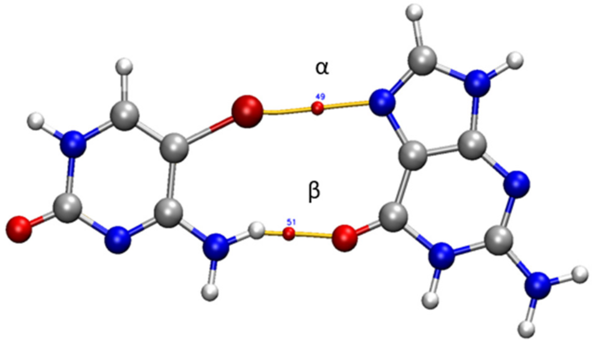

Table S30.

Complex	ρ·10² (α)	ρ·10² (β)
**93 (FC···G)**	0.31	1.91
**94 (ClC···G)**	0.93	1.92
**95 (BrC···G)**	1.16	1.70
**96 (IC···G)**	1.49	1.44

C···8HalG – binding mode d

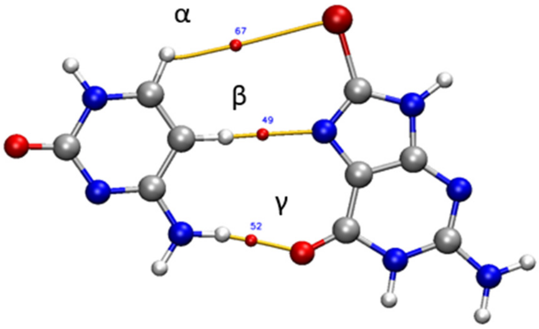

Table S31.

Complex	ρ·10^2 (α)	ρ·10^2 (β)	ρ·10^2 (γ)
**97 (C···FG)**	-	0.95	1.99
**98 (C···ClG)**	-	1.07	1.95
**99 (C···BrG)**	0.057	1.11	1.93
**100 (C···IG)**	0.001	1.20	1.88

5HalC···8HalG – binding mode d

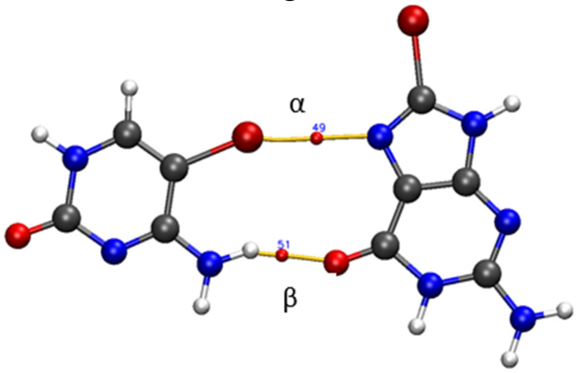

Table S32.

Complex	ρ·10^2 (α)	ρ·10^2 (β)
**101 (FC···G)**	0.47	2.01
**102 (ClC···G)**	0.90	1.82
**103 (BrC···G)**	1.15	1.69
**104 (IC···G)**	1.48	1.43

Cartesian coordinates of complexes **1** to **104**:

Binding mode a

1 (U···A)

C	0.6885691	-2.8865830	0.0000000
C	-0.6181847	-3.3736194	0.0000000
C	-1.4914857	-1.3570324	0.0000000
C	0.8365593	-1.4861380	0.0000000
C	0.8696073	-4.9960881	0.0000000
N	1.6135980	-3.9034446	0.0000000
N	-0.4733607	-4.7384795	0.0000000
H	-1.2257897	-5.4112193	0.0000000
N	2.0332729	-0.8858887	0.0000000
N	-0.2861081	-0.7493506	0.0000000
N	-1.7513249	-2.6600404	0.0000000
H	2.8578550	-1.4625518	0.0000000
H	2.1009033	0.1308524	0.0000000
C	-1.5552268	2.6432498	0.0000000
C	0.9158030	2.7044922	0.0000000
C	0.8460648	4.1527049	0.0000000
C	-0.3622202	4.7536235	0.0000000
H	-0.3007713	1.0105900	0.0000000
H	-0.4807599	5.8299974	0.0000000
H	-2.4243178	4.4815011	0.0000000
N	-0.3108538	2.0619152	0.0000000
O	1.9638658	2.0580437	0.0000000
O	-2.6012651	2.0187062	0.0000000
N	-1.5217210	4.0298932	0.0000000
H	-2.3413289	-0.6814768	0.0000000
H	1.2552030	-6.0047335	0.0000000
H	1.7634171	4.7210764	0.0000000

2 (FU···A)

C	0.6719770	-2.8901323	0.0000000
C	-0.6355895	-3.3744450	0.0000000
C	-1.5047850	-1.3553996	0.0000000
C	0.8231757	-1.4901866	0.0000000
C	0.8488085	-4.9997583	0.0000000
N	1.5950445	-3.9085720	0.0000000
N	-0.4936364	-4.7393817	0.0000000
H	-1.2472768	-5.4109678	0.0000000
N	2.0223973	-0.8947717	0.0000000
N	-0.2974889	-0.7494595	0.0000000
N	-1.7667195	-2.6575581	0.0000000
H	2.8455629	-1.4735308	0.0000000
H	2.0956378	0.1202605	0.0000000
C	-1.5606835	2.6474166	0.0000000
C	0.9169639	2.6783567	0.0000000
C	0.8329516	4.1303626	0.0000000
C	-0.3579428	4.7565150	0.0000000
H	-0.3106174	1.0010682	0.0000000
H	-0.4405515	5.8350428	0.0000000
H	-2.4196596	4.4891365	0.0000000
N	-0.3144396	2.0565064	0.0000000
O	1.9710930	2.0497450	0.0000000
O	-2.6050706	2.0201238	0.0000000
N	-1.5216423	4.0289677	0.0000000
F	1.9742257	4.8189923	0.0000000
H	-2.3539370	-0.6790427	0.0000000
H	1.2322026	-6.0092878	0.0000000

3 (ClU···A)

C	0.6675481	-2.8980175	0.0000000
C	-0.6388344	-3.3854284	0.0000000
C	-1.5119389	-1.3680944	0.0000000
C	0.8158550	-1.4977122	0.0000000

C	0.8492867	-5.0073318	0.0000000
N	1.5929318	-3.9144453	0.0000000
N	-0.4938616	-4.7501576	0.0000000
H	-1.2458144	-5.4235200	0.0000000
N	2.0137589	-0.8999044	0.0000000
N	-0.3061442	-0.7590869	0.0000000
N	-1.7714125	-2.6707606	0.0000000
H	2.8378055	-1.4773147	0.0000000
H	2.0850685	0.1153004	0.0000000
C	-1.5761112	2.6311542	0.0000000
C	0.8998236	2.6825071	0.0000000
C	0.8201231	4.1406756	0.0000000
C	-0.3876559	4.7461966	0.0000000
H	-0.3176350	0.9955282	0.0000000
H	-0.4931134	5.8234427	0.0000000
H	-2.4446803	4.4711097	0.0000000
N	-0.3287600	2.0507157	0.0000000
O	1.9486431	2.0466747	0.0000000
O	-2.6191993	2.0029011	0.0000000
N	-1.5434231	4.0158540	0.0000000
Cl	2.2747387	5.0392824	0.0000000
H	-2.3623741	-0.6936686	0.0000000
H	1.2353754	-6.0159000	0.0000000

4 (BrU···A)

C	0.6678843	-2.8983563	0.0000000
C	-0.6386500	-3.3855280	0.0000000
C	-1.5119658	-1.3684159	0.0000000
C	0.8163813	-1.4981209	0.0000000
C	0.8493243	-5.0075925	0.0000000
N	1.5931293	-3.9147996	0.0000000
N	-0.4937653	-4.7502049	0.0000000
H	-1.2458803	-5.4233840	0.0000000
N	2.0140110	-0.9000969	0.0000000
N	-0.3060383	-0.7598436	0.0000000
N	-1.7713289	-2.6710505	0.0000000
H	2.8385947	-1.4768126	0.0000000
H	2.0833089	0.1154160	0.0000000
C	-1.5869239	2.6243040	0.0000000
C	0.8889730	2.6761360	0.0000000
C	0.8095076	4.1334572	0.0000000
C	-0.3985358	4.7394521	0.0000000
H	-0.3273598	0.9883048	0.0000000
H	-0.5075582	5.8164716	0.0000000
H	-2.4554967	4.4652331	0.0000000
N	-0.3400844	2.0441138	0.0000000
O	1.9361148	2.0370962	0.0000000
O	-2.6304403	1.9968655	0.0000000
N	-1.5541026	4.0098606	0.0000000
Br	2.3982882	5.1174575	0.0000000
H	-2.3624163	-0.6937171	0.0000000
H	1.2350291	-6.0162455	0.0000000

5 (IU···A)

C	0.6679223	-2.8990674	0.0000000
C	-0.6386415	-3.3863130	0.0000000
C	-1.5123820	-1.3696922	0.0000000
C	0.8162673	-1.4989373	0.0000000
C	0.8494447	-5.0082779	0.0000000
N	1.5931888	-3.9154594	0.0000000
N	-0.4936748	-4.7509411	0.0000000
H	-1.2459198	-5.4238785	0.0000000
N	2.0135492	-0.9003145	0.0000000
N	-0.3065261	-0.7612732	0.0000000
N	-1.7715611	-2.6722914	0.0000000
H	2.8384341	-1.4765269	0.0000000
H	2.0809792	0.1154774	0.0000000
C	-1.5992665	2.6161775	0.0000000
C	0.8751821	2.6725503	0.0000000

C	0.7995595	4.1295634	0.0000000
C	-0.4118824	4.7318660	0.0000000
H	-0.3363847	0.9809989	0.0000000
H	-0.5294319	5.8082602	0.0000000
H	-2.4688115	4.4575036	0.0000000
N	-0.3524650	2.0374581	0.0000000
O	1.9217879	2.0308055	0.0000000
O	-2.6430461	1.9890707	0.0000000
N	-1.5669298	4.0023107	0.0000000
I	2.5481798	5.2026008	0.0000000
H	-2.3627281	-0.6947889	0.0000000
H	1.2351564	-6.0168814	0.0000000

6 (U···FA)

C	0.6844385	-2.8832493	0.0000000
C	-0.6181403	-3.3783654	0.0000000
C	-1.5046639	-1.3699125	0.0000000
C	0.8244580	-1.4857371	0.0000000
C	0.8792889	-4.9625813	0.0000000
N	1.6262432	-3.8991231	0.0000000
N	-0.4638024	-4.7518852	0.0000000
H	-1.1917787	-5.4511479	0.0000000
N	2.0147467	-0.8713357	0.0000000
N	-0.3061012	-0.7562689	0.0000000
N	-1.7537883	-2.6780989	0.0000000
H	2.8487728	-1.4338735	0.0000000
H	2.0687064	0.1466756	0.0000000
C	-1.5628571	2.6720146	0.0000000
C	0.9090457	2.7108406	0.0000000
C	0.8520950	4.1590820	0.0000000
C	-0.3504269	4.7713364	0.0000000
H	-0.3226365	1.0299201	0.0000000
H	-0.4584137	5.8488002	0.0000000
H	-2.4147750	4.5192556	0.0000000
N	-0.3232820	2.0785720	0.0000000
O	1.9517569	2.0561471	0.0000000
O	-2.6135681	2.0557901	0.0000000
N	-1.5166611	4.0583961	0.0000000
H	-2.3618389	-0.7039335	0.0000000
H	1.7752573	4.7179431	0.0000000
F	1.3279247	-6.1992613	0.0000000

7 (U···ClA)

C	0.6775648	-2.8715688	0.0000000
C	-0.6279444	-3.3621630	0.0000000
C	-1.5054951	-1.3491579	0.0000000
C	0.8229714	-1.4715607	0.0000000
C	0.8650954	-4.9686858	0.0000000
N	1.6105767	-3.8860817	0.0000000
N	-0.4794408	-4.7303803	0.0000000
H	-1.2152216	-5.4223797	0.0000000
N	2.0158205	-0.8641823	0.0000000
N	-0.3035957	-0.7385930	0.0000000
N	-1.7613011	-2.6547686	0.0000000
H	2.8466557	-1.4318134	0.0000000
H	2.0755825	0.1535081	0.0000000
C	-1.5633293	2.6812856	0.0000000
C	0.9084855	2.7285525	0.0000000
C	0.8464165	4.1767491	0.0000000
C	-0.3585517	4.7845092	0.0000000
H	-0.3173876	1.0441036	0.0000000
H	-0.4710154	5.8614051	0.0000000
H	-2.4218905	4.5250433	0.0000000
N	-0.3218379	2.0927422	0.0000000
O	1.9523512	2.0756720	0.0000000
O	-2.6118377	2.0612520	0.0000000
N	-1.5221896	4.0675966	0.0000000
H	-2.3588377	-0.6782285	0.0000000
H	1.7668086	4.7400332	0.0000000

Cl	1.4515473	-6.5628891	0.0000000

8 (U···BrA)

C	0.6754310	-2.8575550	0.0000000
C	-0.6329981	-3.3424697	0.0000000
C	-1.5047362	-1.3276439	0.0000000
C	0.8253208	-1.4577132	0.0000000
C	0.8527945	-4.9575079	0.0000000
N	1.6033569	-3.8762442	0.0000000
N	-0.4903469	-4.7107145	0.0000000
H	-1.2321887	-5.3961002	0.0000000
N	2.0169977	-0.8489409	0.0000000
N	-0.2999895	-0.7234239	0.0000000
N	-1.7649440	-2.6319351	0.0000000
H	2.8503219	-1.4131401	0.0000000
H	2.0726360	0.1696116	0.0000000
C	-1.5590184	2.6669817	0.0000000
C	0.9116011	2.7256277	0.0000000
C	0.8436595	4.1731663	0.0000000
C	-0.3643420	4.7752925	0.0000000
H	-0.3072426	1.0340416	0.0000000
H	-0.4818882	5.8516361	0.0000000
H	-2.4265317	4.5063658	0.0000000
N	-0.3151881	2.0845510	0.0000000
O	1.9588440	2.0775180	0.0000000
O	-2.6046224	2.0415282	0.0000000
N	-1.5246383	4.0532147	0.0000000
H	-2.3544792	-0.6517858	0.0000000
H	1.7618631	4.7396751	0.0000000
Br	1.4903278	-6.7040358	0.0000000

9 (U···IA)

C	0.6716386	-2.8575366	0.0000000
C	-0.6313500	-3.3557186	0.0000000
C	-1.5198557	-1.3466460	0.0000000
C	0.8091464	-1.4556536	0.0000000
C	0.8723524	-4.9598022	0.0000000
N	1.6098611	-3.8647634	0.0000000
N	-0.4748655	-4.7218663	0.0000000
H	-1.2115981	-5.4133392	0.0000000
N	1.9993956	-0.8445467	0.0000000
N	-0.3208973	-0.7286658	0.0000000
N	-1.7689962	-2.6532098	0.0000000
H	2.8310230	-1.4111820	0.0000000
H	2.0567292	0.1737341	0.0000000
C	-1.5699288	2.6992755	0.0000000
C	0.9017833	2.7353568	0.0000000
C	0.8465288	4.1836284	0.0000000
C	-0.3554385	4.7970935	0.0000000
H	-0.3323886	1.0557651	0.0000000
H	-0.4625567	5.8745773	0.0000000
H	-2.4198235	4.5471142	0.0000000
N	-0.3310449	2.1045923	0.0000000
O	1.9436779	2.0790880	0.0000000
O	-2.6213920	2.0843995	0.0000000
N	-1.5222283	4.0854899	0.0000000
H	-2.3769513	-0.6803451	0.0000000
I	1.6073007	-6.8688603	0.0000000
H	1.7698784	4.7420209	0.0000000

10 (FU···FA)

C	0.6777779	-2.8814177	0.0000000
C	-0.6288977	-3.3660214	0.0000000
C	-1.4994061	-1.3503796	0.0000000
C	0.8296699	-1.4854200	0.0000000
C	0.8555173	-4.9621167	0.0000000
N	1.6110173	-3.9045795	0.0000000
N	-0.4860069	-4.7403871	0.0000000

H	-1.2198874	-5.4336469	0.0000000
N	2.0262449	-0.8827602	0.0000000
N	-0.2948389	-0.7467360	0.0000000
N	-1.7584280	-2.6559564	0.0000000
H	2.8552628	-1.4528820	0.0000000
H	2.0915718	0.1329992	0.0000000
C	-1.5727039	2.6513584	0.0000000
C	0.9057068	2.6904750	0.0000000
C	0.8166408	4.1420276	0.0000000
C	-0.3766123	4.7640225	0.0000000
H	-0.3161986	1.0122029	0.0000000
H	-0.4631598	5.8423121	0.0000000
H	-2.4375533	4.4903278	0.0000000
N	-0.3240938	2.0654076	0.0000000
O	1.9608949	2.0637891	0.0000000
O	-2.6139571	2.0189630	0.0000000
N	-1.5381618	4.0327126	0.0000000
H	-2.3502231	-0.6764284	0.0000000
F	1.2942015	-6.2018453	0.0000000
F	1.9556226	4.8339792	0.0000000

11 (ClU···ClA)

C	0.6604702	-2.8781309	0.0000000
C	-0.6470932	-3.3633198	0.0000000
C	-1.5174353	-1.3467969	0.0000000
C	0.8115774	-1.4790194	0.0000000
C	0.8393983	-4.9757432	0.0000000
N	1.5893648	-3.8959131	0.0000000
N	-0.5043827	-4.7318247	0.0000000
H	-1.2438009	-5.4201020	0.0000000
N	2.0074621	-0.8777140	0.0000000
N	-0.3122271	-0.7411748	0.0000000
N	-1.7775843	-2.6510202	0.0000000
H	2.8362687	-1.4483644	0.0000000
H	2.0719294	0.1384985	0.0000000
C	-1.5877010	2.6575402	0.0000000
C	0.8893635	2.7011221	0.0000000
C	0.8146465	4.1591125	0.0000000
C	-0.3914594	4.7681452	0.0000000
H	-0.3321019	1.0208590	0.0000000
H	-0.4929179	5.8457576	0.0000000
H	-2.4490558	4.5012022	0.0000000
N	-0.3416148	2.0740071	0.0000000
O	1.9353552	2.0607336	0.0000000
O	-2.6318979	2.0311958	0.0000000
N	-1.5498823	4.0418424	0.0000000
H	-2.3681504	-0.6724091	0.0000000
Cl	2.2717748	5.0531249	0.0000000
Cl	1.4196939	-6.5716085	0.0000000

12 (BrU···BrA)

C	0.6533886	-2.8752807	0.0000000
C	-0.6543939	-3.3603768	0.0000000
C	-1.5243995	-1.3437333	0.0000000
C	0.8047483	-1.4756300	0.0000000
C	0.8318841	-4.9745655	0.0000000
N	1.5820643	-3.8929428	0.0000000
N	-0.5119215	-4.7286961	0.0000000
H	-1.2528659	-5.4155509	0.0000000
N	2.0006544	-0.8750963	0.0000000
N	-0.3189521	-0.7379739	0.0000000
N	-1.7849578	-2.6476707	0.0000000
H	2.8289282	-1.4465976	0.0000000
H	2.0655890	0.1411849	0.0000000
C	-1.5942186	2.6594619	0.0000000
C	0.8829379	2.7035591	0.0000000
C	0.8083938	4.1608098	0.0000000
C	-0.3980058	4.7701851	0.0000000
H	-0.3391846	1.0225909	0.0000000

H	-0.5038909	5.8474952	0.0000000
H	-2.4554990	4.5040419	0.0000000
N	-0.3486461	2.0759870	0.0000000
O	1.9272006	2.0598796	0.0000000
O	-2.6388577	2.0341474	0.0000000
N	-1.5561592	4.0445761	0.0000000
H	-2.3748747	-0.6690919	0.0000000
Br	2.3999770	5.1398476	0.0000000
Br	1.4710608	-6.7205598	0.0000000

13 (IU···IA)

C	0.6439382	-2.8725454	0.0000000
C	-0.6641176	-3.3576166	0.0000000
C	-1.5329909	-1.3399775	0.0000000
C	0.7958016	-1.4723240	0.0000000
C	0.8229143	-4.9768639	0.0000000
N	1.5715223	-3.8891937	0.0000000
N	-0.5218347	-4.7248766	0.0000000
H	-1.2663707	-5.4080640	0.0000000
N	1.9921883	-0.8728424	0.0000000
N	-0.3271434	-0.7341607	0.0000000
N	-1.7945471	-2.6435225	0.0000000
H	2.8195266	-1.4458014	0.0000000
H	2.0577759	0.1435915	0.0000000
C	-1.6015753	2.6621178	0.0000000
C	0.8738775	2.7091825	0.0000000
C	0.8034533	4.1662587	0.0000000
C	-0.4058830	4.7729033	0.0000000
H	-0.3463229	1.0255055	0.0000000
H	-0.5184332	5.8498876	0.0000000
H	-2.4639612	4.5064688	0.0000000
N	-0.3564281	2.0791166	0.0000000
O	1.9172663	2.0623359	0.0000000
O	-2.6464410	2.0371642	0.0000000
N	-1.5639006	4.0477512	0.0000000
H	-2.3829628	-0.6647253	0.0000000
I	2.5561227	5.2330271	0.0000000
I	1.5385255	-6.8927968	0.0000000

Binding mode b

14 (U···A)

C	0.2735084	-2.0496666	0.0000000
C	-0.4460925	-3.2445133	0.0000000
C	1.3836141	-4.4569892	0.0000000
C	1.6797362	-2.1605690	0.0000000
C	-1.7916372	-1.4965618	0.0000000
N	-0.5769901	-0.9693462	0.0000000
N	-1.7666659	-2.8571938	0.0000000
H	-2.5620727	-3.4785731	0.0000000
N	2.4982011	-1.0982186	0.0000000
N	2.2059572	-3.3940757	0.0000000
N	0.0497981	-4.4846408	0.0000000
H	2.1584739	-0.1406686	0.0000000
H	3.4886989	-1.2790300	0.0000000
C	-1.6963073	2.3931779	0.0000000
C	0.7756076	2.4522812	0.0000000
C	0.7058160	3.9006243	0.0000000
C	-0.5026537	4.5011751	0.0000000
H	-0.4460030	0.7659592	0.0000000
H	-0.6207865	5.5775103	0.0000000
H	-2.5657462	4.2318240	0.0000000
N	-0.4536903	1.8139592	0.0000000
O	1.8180694	1.7996325	0.0000000
O	-2.7424973	1.7642625	0.0000000
N	-1.6642306	3.7780905	0.0000000
H	1.8778568	-5.4230274	0.0000000
H	-2.7026680	-0.9151779	0.0000000
H	1.6227034	4.4697551	0.0000000

15 (FU···A)

C	0.2580269	-2.0521217	0.0000000
C	-0.4648108	-3.2451802	0.0000000
C	1.3610541	-4.4637312	0.0000000
C	1.6638514	-2.1685858	0.0000000
C	-1.8064953	-1.4940310	0.0000000
N	-0.5902212	-0.9691202	0.0000000
N	-1.7843589	-2.8542327	0.0000000
H	-2.5812220	-3.4739528	0.0000000
N	2.4867844	-1.1095104	0.0000000
N	2.1861539	-3.4031494	0.0000000
N	0.0271427	-4.4866473	0.0000000
H	2.1514335	-0.1519540	0.0000000
H	3.4768833	-1.2927774	0.0000000
C	-1.6977760	2.3946855	0.0000000
C	0.7808213	2.4309405	0.0000000
C	0.6921327	3.8830079	0.0000000
C	-0.5011934	4.5049949	0.0000000
H	-0.4497081	0.7581194	0.0000000
H	-0.5869021	5.5832366	0.0000000
H	-2.5629409	4.2338760	0.0000000
N	-0.4514879	1.8098573	0.0000000
O	1.8311359	1.7987881	0.0000000
O	-2.7403370	1.7598234	0.0000000
N	-1.6644972	3.7745352	0.0000000
F	1.8302927	4.5755217	0.0000000
H	1.8530250	-5.4308989	0.0000000
H	-2.7167869	-0.9114937	0.0000000

16 (ClU···A)

C	0.2538943	-2.0600963	0.0000000
C	-0.4658554	-3.2549770	0.0000000
C	1.3629759	-4.4690469	0.0000000
C	1.6600587	-2.1729991	0.0000000
C	-1.8120373	-1.5075391	0.0000000
N	-0.5972150	-0.9792015	0.0000000
N	-1.7863788	-2.8677066	0.0000000
H	-2.5815871	-3.4896187	0.0000000
N	2.4799452	-1.1117119	0.0000000
N	2.1854748	-3.4064312	0.0000000
N	0.0291930	-4.4953160	0.0000000
H	2.1407925	-0.1552469	0.0000000
H	3.4705463	-1.2918526	0.0000000
C	-1.7178972	2.3812268	0.0000000
C	0.7589448	2.4296506	0.0000000
C	0.6799231	3.8878965	0.0000000
C	-0.5279717	4.4934989	0.0000000
H	-0.4648448	0.7512045	0.0000000
H	-0.6320242	5.5708861	0.0000000
H	-2.5859707	4.2218837	0.0000000
N	-0.4724469	1.8026985	0.0000000
O	1.8021916	1.7874493	0.0000000
O	-2.7610570	1.7484521	0.0000000
N	-1.6860220	3.7641456	0.0000000
Cl	2.1342608	4.7856540	0.0000000
H	1.8570543	-5.4351962	0.0000000
H	-2.7239474	-0.9277064	0.0000000

17 (BrU···A)

C	0.2538798	-2.0605227	0.0000000
C	-0.4658843	-3.2554239	0.0000000
C	1.3630138	-4.4692512	0.0000000
C	1.6601489	-2.1731836	0.0000000
C	-1.8124316	-1.5082854	0.0000000
N	-0.5976277	-0.9800903	0.0000000
N	-1.7865344	-2.8683153	0.0000000
H	-2.5818129	-3.4900854	0.0000000

N	2.4799058	-1.1118669	0.0000000
N	2.1855194	-3.4066622	0.0000000
N	0.0292306	-4.4956932	0.0000000
H	2.1401302	-0.1554905	0.0000000
H	3.4705265	-1.2919705	0.0000000
C	-1.7283316	2.3742310	0.0000000
C	0.7481473	2.4240016	0.0000000
C	0.6691991	3.8814983	0.0000000
C	-0.5390755	4.4871017	0.0000000
H	-0.4742392	0.7445499	0.0000000
H	-0.6462077	5.5642937	0.0000000
H	-2.5971805	4.2149707	0.0000000
N	-0.4833176	1.7965848	0.0000000
O	1.7899566	1.7791011	0.0000000
O	-2.7716615	1.7417867	0.0000000
N	-1.6967769	3.7578953	0.0000000
Br	2.2583190	4.8640625	0.0000000
H	1.8571000	-5.4353726	0.0000000
H	-2.7239957	-0.9278635	0.0000000

18 (IU···A)

C	0.2499153	-2.0665679	0.0000000
C	-0.4676395	-3.2627458	0.0000000
C	1.3636245	-4.4732199	0.0000000
C	1.6562242	-2.1766863	0.0000000
C	-1.8168780	-1.5175683	0.0000000
N	-0.6029772	-0.9871571	0.0000000
N	-1.7888717	-2.8777231	0.0000000
H	-2.5830243	-3.5008810	0.0000000
N	2.4731371	-1.1130730	0.0000000
N	2.1841650	-3.4089955	0.0000000
N	0.0298370	-4.5021286	0.0000000
H	2.1303791	-0.1578003	0.0000000
H	3.4642882	-1.2899653	0.0000000
C	-1.7343602	2.3683668	0.0000000
C	0.7409141	2.4297220	0.0000000
C	0.6610913	3.8868737	0.0000000
C	-0.5522747	4.4853376	0.0000000
H	-0.4751801	0.7435251	0.0000000
H	-0.6724258	5.5615217	0.0000000
H	-2.6094692	4.2065733	0.0000000
N	-0.4876590	1.7951073	0.0000000
O	1.7836133	1.7845345	0.0000000
O	-2.7760877	1.7334888	0.0000000
N	-1.7071680	3.7526191	0.0000000
I	2.4069465	4.9643858	0.0000000
H	1.8596499	-5.4383565	0.0000000
H	-2.7297700	-0.9391872	0.0000000

19 (U···FA)

C	0.2452660	-2.1217692	0.0000000
C	-0.4005119	-3.3579012	0.0000000
C	1.4880228	-4.4705009	0.0000000
C	1.6529647	-2.1585644	0.0000000
C	-1.8181089	-1.6992281	0.0000000
N	-0.6726536	-1.0758520	0.0000000
N	-1.7500282	-3.0513119	0.0000000
H	-2.5248131	-3.6984367	0.0000000
N	2.4233786	-1.0623687	0.0000000
N	2.2449477	-3.3655867	0.0000000
N	0.1552877	-4.5673293	0.0000000
H	2.0465931	-0.1183934	0.0000000
H	3.4202424	-1.2034628	0.0000000
C	-1.7322935	2.5639692	0.0000000
C	0.7421340	2.4506469	0.0000000
C	0.7735978	3.8988229	0.0000000
C	-0.3878997	4.5844535	0.0000000
H	-0.5866907	0.8611880	0.0000000
H	-0.4291804	5.6663302	0.0000000

H	-2.4629458	4.4595445	0.0000000
N	-0.5278930	1.8933064	0.0000000
O	1.7419141	1.7360006	0.0000000
O	-2.8180340	2.0179888	0.0000000
N	-1.5950234	3.9444956	0.0000000
H	2.0330481	-5.4086961	0.0000000
H	1.7300814	4.3979192	0.0000000
F	-2.9914020	-1.1152646	0.0000000

20 (U···ClA)

C	0.2547932	-2.1563484	0.0000000
C	-0.3632929	-3.4088742	0.0000000
C	1.5482019	-4.4828635	0.0000000
C	1.6666411	-2.1680762	0.0000000
C	-1.8341006	-1.7797952	0.0000000
N	-0.6821455	-1.1352048	0.0000000
N	-1.7118708	-3.1341290	0.0000000
H	-2.4672908	-3.8045328	0.0000000
N	2.4150838	-1.0585352	0.0000000
N	2.2826983	-3.3617359	0.0000000
N	0.2193793	-4.6078906	0.0000000
H	2.0185304	-0.1229007	0.0000000
H	3.4144870	-1.1811076	0.0000000
C	-1.7496780	2.6382392	0.0000000
C	0.7191973	2.4448420	0.0000000
C	0.7977572	3.8910812	0.0000000
C	-0.3395295	4.6150693	0.0000000
H	-0.6519060	0.8951836	0.0000000
H	-0.3457033	5.6977934	0.0000000
H	-2.4172620	4.5548922	0.0000000
N	-0.5684156	1.9245917	0.0000000
O	1.6978131	1.7028744	0.0000000
O	-2.8556207	2.1351536	0.0000000
N	-1.5656222	4.0131176	0.0000000
H	2.1134409	-5.4092510	0.0000000
H	1.7707986	4.3570828	0.0000000
Cl	-3.3663842	-1.0586758	0.0000000

21 (U···BrA)

C	0.2536247	-2.1738646	0.0000000
C	-0.3493398	-3.4343694	0.0000000
C	1.5738480	-4.4872296	0.0000000
C	1.6664582	-2.1711022	0.0000000
C	-1.8411618	-1.8233721	0.0000000
N	-0.6956792	-1.1635042	0.0000000
N	-1.7002967	-3.1762590	0.0000000
H	-2.4464335	-3.8571911	0.0000000
N	2.4041843	-1.0548319	0.0000000
N	2.2954704	-3.3577478	0.0000000
N	0.2469129	-4.6271255	0.0000000
H	1.9994151	-0.1230331	0.0000000
H	3.4047516	-1.1678501	0.0000000
C	-1.7500123	2.6716018	0.0000000
C	0.7164823	2.4490490	0.0000000
C	0.8116269	3.8942030	0.0000000
C	-0.3166844	4.6317910	0.0000000
H	-0.6726302	0.9156233	0.0000000
H	-0.3103541	5.7144736	0.0000000
H	-2.3949499	4.5953589	0.0000000
N	-0.5773890	1.9429334	0.0000000
O	1.6869111	1.6970586	0.0000000
O	-2.8622225	2.1828048	0.0000000
N	-1.5494997	4.0438994	0.0000000
H	2.1498233	-5.4069931	0.0000000
H	1.7905133	4.3477317	0.0000000
Br	-3.5333691	-1.0620549	0.0000000

22 (U···IA)

C	0.2464153	-2.1883196	0.0000000
C	-0.3493365	-3.4531130	0.0000000
C	1.5782061	-4.4980385	0.0000000
C	1.6604242	-2.1811405	0.0000000
C	-1.8559456	-1.8501568	0.0000000
N	-0.7088689	-1.1840866	0.0000000
N	-1.6996808	-3.2034271	0.0000000
H	-2.4389767	-3.8919833	0.0000000
N	2.3966256	-1.0634570	0.0000000
N	2.2944664	-3.3648771	0.0000000
N	0.2524699	-4.6436075	0.0000000
H	1.9925843	-0.1319186	0.0000000
H	3.3972787	-1.1768696	0.0000000
C	-1.7330451	2.6978961	0.0000000
C	0.7306994	2.4575700	0.0000000
C	0.8353691	3.9024407	0.0000000
C	-0.2874269	4.6484318	0.0000000
H	-0.6694891	0.9330376	0.0000000
H	-0.2754843	5.7310465	0.0000000
H	-2.3663794	4.6244239	0.0000000
N	-0.5668663	1.9594219	0.0000000
O	1.6956417	1.6991585	0.0000000
O	-2.8491921	2.2176572	0.0000000
N	-1.5238775	4.0681885	0.0000000
H	2.1582829	-5.4153091	0.0000000
I	-3.7317790	-1.0409461	0.0000000
H	1.8178846	4.3479776	0.0000000

23 (FU···FA)

C	0.2442675	-2.1193721	0.0000000
C	-0.4084053	-3.3520375	0.0000000
C	1.4733290	-4.4759111	0.0000000
C	1.6516903	-2.1651661	0.0000000
C	-1.8179693	-1.6867635	0.0000000
N	-0.6690313	-1.0687605	0.0000000
N	-1.7562882	-3.0385642	0.0000000
H	-2.5343233	-3.6819278	0.0000000
N	2.4305606	-1.0744746	0.0000000
N	2.2363264	-3.3753320	0.0000000
N	0.1401297	-4.5644246	0.0000000
H	2.0601634	-0.1296895	0.0000000
H	3.4265851	-1.2213555	0.0000000
C	-1.7486648	2.5560298	0.0000000
C	0.7321963	2.4297138	0.0000000
C	0.7394332	3.8835814	0.0000000
C	-0.4084159	4.5844559	0.0000000
H	-0.5957629	0.8505897	0.0000000
H	-0.4231786	5.6661111	0.0000000
H	-2.4823906	4.4483095	0.0000000
N	-0.5378023	1.8851697	0.0000000
O	1.7437032	1.7387930	0.0000000
O	-2.8287717	1.9991619	0.0000000
N	-1.6155440	3.9317340	0.0000000
H	2.0125777	-5.4173292	0.0000000
F	-2.9877948	-1.0967953	0.0000000
F	1.9233806	4.4942538	0.0000000

24 (ClU···ClA)

C	0.2344697	-2.1623753	0.0000000
C	-0.3915524	-3.4111374	0.0000000
C	1.5120700	-4.4985731	0.0000000
C	1.6464617	-2.1848059	0.0000000
C	-1.8532975	-1.7742438	0.0000000
N	-0.6974081	-1.1356517	0.0000000
N	-1.7383253	-3.1286713	0.0000000
H	-2.4972932	-3.7951441	0.0000000
N	2.4044470	-1.0814706	0.0000000
N	2.2538807	-3.3824382	0.0000000
N	0.1825270	-4.6140127	0.0000000

H	2.0142533	-0.1447323	0.0000000
H	3.4030594	-1.2105188	0.0000000
C	-1.7719732	2.6298349	0.0000000
C	0.7012903	2.4202944	0.0000000
C	0.7757117	3.8775936	0.0000000
C	-0.3595338	4.6079381	0.0000000
H	-0.6722915	0.8836066	0.0000000
H	-0.3508369	5.6902541	0.0000000
H	-2.4326286	4.5495576	0.0000000
N	-0.5888684	1.9149629	0.0000000
O	1.6789226	1.6850087	0.0000000
O	-2.8766768	2.1256246	0.0000000
N	-1.5842937	4.0018661	0.0000000
H	2.0710700	-5.4286042	0.0000000
Cl	2.3186439	4.6116083	0.0000000
Cl	-3.3818280	-1.0457704	0.0000000

25 (BrU···BrA)

C	0.2272332	-2.1837084	0.0000000
C	-0.3844664	-3.4401610	0.0000000
C	1.5303263	-4.5077742	0.0000000
C	1.6402386	-2.1925583	0.0000000
C	-1.8661518	-1.8201800	0.0000000
N	-0.7162619	-1.1669605	0.0000000
N	-1.7334893	-3.1733864	0.0000000
H	-2.4837952	-3.8498144	0.0000000
N	2.3880898	-1.0827355	0.0000000
N	2.2600686	-3.3836460	0.0000000
N	0.2024955	-4.6372162	0.0000000
H	1.9901194	-0.1495803	0.0000000
H	3.3877873	-1.2032044	0.0000000
C	-1.7761771	2.6630415	0.0000000
C	0.6949319	2.4243554	0.0000000
C	0.7866146	3.8800238	0.0000000
C	-0.3399653	4.6242389	0.0000000
H	-0.6973486	0.9039311	0.0000000
H	-0.3230687	5.7066069	0.0000000
H	-2.4133949	4.5908118	0.0000000
N	-0.6023060	1.9331133	0.0000000
O	1.6615227	1.6748797	0.0000000
O	-2.8877219	2.1746593	0.0000000
N	-1.5713106	4.0331965	0.0000000
H	2.0991503	-5.4318678	0.0000000
Br	2.4814073	4.6650233	0.0000000
Br	-3.5545279	-1.0510882	0.0000000

26 (IU···IA)

C	0.2137805	-2.2015291	0.0000000
C	-0.3828474	-3.4658471	0.0000000
C	1.5434985	-4.5131680	0.0000000
C	1.6278795	-2.1967919	0.0000000
C	-1.8888824	-1.8623554	0.0000000
N	-0.7412449	-1.1962775	0.0000000
N	-1.7329805	-3.2154541	0.0000000
H	-2.4722391	-3.9041968	0.0000000
N	2.3659147	-1.0805443	0.0000000
N	2.2606775	-3.3807758	0.0000000
N	0.2176291	-4.6568699	0.0000000
H	1.9619538	-0.1502750	0.0000000
H	3.3665534	-1.1932379	0.0000000
C	-1.7688006	2.6837438	0.0000000
C	0.6993929	2.4336928	0.0000000
C	0.8038442	3.8880660	0.0000000
C	-0.3214983	4.6363335	0.0000000
H	-0.7023029	0.9186051	0.0000000
H	-0.3058680	5.7190247	0.0000000
H	-2.3954291	4.6145006	0.0000000
N	-0.6002686	1.9465870	0.0000000
O	1.6600473	1.6757297	0.0000000

O	-2.8837342	2.2026459	0.0000000
N	-1.5559411	4.0525504	0.0000000
H	2.1225979	-5.4309528	0.0000000
I	2.6735373	4.7312867	0.0000000
I	-3.7652698	-1.0544908	0.0000000

Binding mode c

27 (U···A)

C	0.5619323	-3.0936289	0.0000000
C	-0.6830581	-3.7209660	0.0000000
C	-1.7554839	-1.8050494	0.0000000
C	0.5568381	-1.6840451	0.0000000
C	0.9723262	-5.1720511	0.0000000
N	1.5924328	-4.0049040	0.0000000
N	-0.3909681	-5.0629320	0.0000000
H	-1.0643513	-5.8145946	0.0000000
N	1.6927343	-0.9731903	0.0000000
N	-0.6328449	-1.0609441	0.0000000
N	-1.8841677	-3.1304747	0.0000000
H	2.5670725	-1.4714287	0.0000000
H	1.6837724	0.0436526	0.0000000
C	0.1117298	5.0965719	0.0000000
C	0.8801278	2.7223367	0.0000000
C	-0.5068173	2.3101771	0.0000000
C	-1.4684524	3.2563700	0.0000000
H	2.0326400	4.4267273	0.0000000
H	-2.5234187	3.0122409	0.0000000
H	-1.9065852	5.2917710	0.0000000
N	1.0716311	4.1030211	0.0000000
O	1.8529006	1.9739441	0.0000000
O	0.3677773	6.2843751	0.0000000
N	-1.1755281	4.5958006	0.0000000
H	-2.6830345	-1.2403332	0.0000000
H	1.4657847	-6.1326934	0.0000000
H	-0.7349898	1.2502468	0.0000000

28 (FU···A)

C	0.5298309	-3.3702799	0.0000000
C	-0.5068976	-4.3027991	0.0000000
C	-2.0448639	-2.7339777	0.0000000
C	0.1491757	-2.0134918	0.0000000
C	1.4722684	-5.2685348	0.0000000
N	1.7643760	-3.9794973	0.0000000
N	0.1284251	-5.5215119	0.0000000
H	-0.3237112	-6.4236122	0.0000000
N	1.0583842	-1.0255888	0.0000000
N	-1.1582160	-1.7220115	0.0000000
N	-1.8200193	-4.0487347	0.0000000
H	2.0357487	-1.2625345	0.0000000
H	0.7817313	-0.0539183	0.0000000
C	0.5553915	5.4806339	0.0000000
C	0.6726265	2.9791268	0.0000000
C	-0.7814243	2.9868052	0.0000000
C	-1.4724584	4.1407943	0.0000000
H	2.2267583	4.3156626	0.0000000
H	-2.5538170	4.1609479	0.0000000
H	-1.3343064	6.2153769	0.0000000
N	1.2138032	4.2591796	0.0000000
O	1.3733185	1.9801243	0.0000000
O	1.1304453	6.5498736	0.0000000
N	-0.8162927	5.3491824	0.0000000
F	-1.3931543	1.8072788	0.0000000
H	-3.0875944	-2.4323267	0.0000000
H	2.2004717	-6.0661674	0.0000000

29 (ClU···A)

C	0.5240375	-3.3694020	0.0000000

C	-0.5082748	-4.3063507	0.0000000
C	-2.0521242	-2.7441751	0.0000000
C	0.1403988	-2.0132967	0.0000000
C	1.4749349	-5.2630690	0.0000000
N	1.7610462	-3.9726987	0.0000000
N	0.1321587	-5.5221408	0.0000000
H	-0.3159811	-6.4262091	0.0000000
N	1.0538724	-1.0291606	0.0000000
N	-1.1693955	-1.7275068	0.0000000
N	-1.8225799	-4.0572712	0.0000000
H	2.0286401	-1.2779759	0.0000000
H	0.7914635	-0.0534847	0.0000000
C	0.5740955	5.5119179	0.0000000
C	0.6847802	3.0127208	0.0000000
C	-0.7752100	3.0050423	0.0000000
C	-1.4531819	4.1731831	0.0000000
H	2.2425881	4.3457887	0.0000000
H	-2.5348413	4.2112609	0.0000000
H	-1.3167537	6.2462984	0.0000000
N	1.2294016	4.2929139	0.0000000
O	1.3985513	2.0230156	0.0000000
O	1.1446695	6.5834240	0.0000000
N	-0.7996648	5.3788405	0.0000000
Cl	-1.5436416	1.4820171	0.0000000
H	-3.0959969	-2.4465383	0.0000000
H	2.2070073	-6.0571435	0.0000000

30 (BrU···A)

C	0.5211001	-3.3991939	0.0000000
C	-0.5067026	-4.3411591	0.0000000
C	-2.0587367	-2.7877163	0.0000000
C	0.1319073	-2.0446873	0.0000000
C	1.4807289	-5.2882724	0.0000000
N	1.7607374	-3.9964919	0.0000000
N	0.1392329	-5.5535328	0.0000000
H	-0.3050469	-6.4595436	0.0000000
N	1.0440475	-1.0585570	0.0000000
N	-1.1802308	-1.7667815	0.0000000
N	-1.8223396	-4.0989186	0.0000000
H	2.0189896	-1.3075127	0.0000000
H	0.7817406	-0.0831311	0.0000000
C	0.5841956	5.5638126	0.0000000
C	0.6898713	3.0631968	0.0000000
C	-0.7698868	3.0593108	0.0000000
C	-1.4452957	4.2288574	0.0000000
H	2.2500156	4.3956028	0.0000000
H	-2.5268255	4.2726555	0.0000000
H	-1.3068450	6.3015180	0.0000000
N	1.2368556	4.3439480	0.0000000
O	1.4055442	2.0744786	0.0000000
O	1.1564253	6.6346398	0.0000000
N	-0.7904600	5.4335536	0.0000000
Br	-1.6009768	1.3880388	0.0000000
H	-3.1040571	-2.4948223	0.0000000
H	2.2160115	-6.0792921	0.0000000

31 (IU···A)

C	0.5233725	-3.4560890	0.0000000
C	-0.4962740	-4.4067171	0.0000000
C	-2.0626512	-2.8686522	0.0000000
C	0.1232261	-2.1046553	0.0000000
C	1.4981809	-5.3372115	0.0000000
N	1.7676361	-4.0430602	0.0000000
N	0.1589912	-5.6133071	0.0000000
H	-0.2793447	-6.5221569	0.0000000
N	1.0304885	-1.1134360	0.0000000
N	-1.1923368	-1.8396749	0.0000000
N	-1.8141123	-4.1766900	0.0000000
H	2.0063679	-1.3595195	0.0000000

H	0.7645206	-0.1391370	0.0000000
C	0.6090028	5.6443191	0.0000000
C	0.6689697	3.1424742	0.0000000
C	-0.7910933	3.1594112	0.0000000
C	-1.4427089	4.3436389	0.0000000
H	2.2533316	4.4470679	0.0000000
H	-2.5233947	4.4135904	0.0000000
H	-1.2690847	6.4145224	0.0000000
N	1.2394575	4.4139338	0.0000000
O	1.3733548	2.1445052	0.0000000
O	1.1994946	6.7055689	0.0000000
N	-0.7679404	5.5374918	0.0000000
I	-1.7063882	1.3218756	0.0000000
H	-3.1105566	-2.5854834	0.0000000
H	2.2394910	-6.1226095	0.0000000

32 (U···FA)

C	0.5645281	-3.0864829	0.0000000
C	-0.6783761	-3.7156260	0.0000000
C	-1.7546675	-1.8047769	0.0000000
C	0.5588222	-1.6809485	0.0000000
C	0.9758619	-5.1350444	0.0000000
N	1.6073874	-3.9997719	0.0000000
N	-0.3820160	-5.0664467	0.0000000
H	-1.0318878	-5.8385691	0.0000000
N	1.6907898	-0.9629129	0.0000000
N	-0.6352903	-1.0615289	0.0000000
N	-1.8780748	-3.1334375	0.0000000
H	2.5698714	-1.4524735	0.0000000
H	1.6732277	0.0541555	0.0000000
C	0.1044168	5.1057570	0.0000000
C	0.8642998	2.7290072	0.0000000
C	-0.5240789	2.3219876	0.0000000
C	-1.4824884	3.2715089	0.0000000
H	2.0230416	4.4286689	0.0000000
H	-2.5381880	3.0310073	0.0000000
H	-1.9134437	5.3082907	0.0000000
N	1.0607977	4.1085671	0.0000000
O	1.8343334	1.9768876	0.0000000
O	0.3648159	6.2924180	0.0000000
N	-1.1848814	4.6095873	0.0000000
H	-2.6843669	-1.2442278	0.0000000
H	-0.7558408	1.2631157	0.0000000
F	1.5514069	-6.3187118	0.0000000

33 (U···ClA)

C	0.5579566	-3.0730883	0.0000000
C	-0.6843365	-3.7057114	0.0000000
C	-1.7639681	-1.7957972	0.0000000
C	0.5488256	-1.6646215	0.0000000
C	0.9772828	-5.1370725	0.0000000
N	1.5979872	-3.9787253	0.0000000
N	-0.3855911	-5.0500390	0.0000000
H	-1.0395872	-5.8195475	0.0000000
N	1.6800421	-0.9474674	0.0000000
N	-0.6455652	-1.0483572	0.0000000
N	-1.8862469	-3.1235365	0.0000000
H	2.5584713	-1.4386967	0.0000000
H	1.6637168	0.0699558	0.0000000
C	0.0997344	5.1186927	0.0000000
C	0.8568850	2.7411689	0.0000000
C	-0.5318717	2.3356151	0.0000000
C	-1.4892398	3.2861944	0.0000000
H	2.0176207	4.4395596	0.0000000
H	-2.5451749	3.0466685	0.0000000
H	-1.9179107	5.3234375	0.0000000
N	1.0550473	4.1204186	0.0000000
O	1.8261321	1.9878536	0.0000000
O	0.3615106	6.3050250	0.0000000

N	-1.1901134	4.6239226	0.0000000
H	-2.6944642	-1.2363071	0.0000000
H	-0.7649514	1.2770551	0.0000000
Cl	1.7378085	-6.6565997	0.0000000

34 (U···BrA)

C	0.5564999	-3.0675895	0.0000000
C	-0.6853743	-3.7014875	0.0000000
C	-1.7664768	-1.7922967	0.0000000
C	0.5461751	-1.6585807	0.0000000
C	0.9777242	-5.1327519	0.0000000
N	1.5972077	-3.9719787	0.0000000
N	-0.3854637	-5.0453706	0.0000000
H	-1.0401953	-5.8144794	0.0000000
N	1.6768520	-0.9410690	0.0000000
N	-0.6486160	-1.0435773	0.0000000
N	-1.8879321	-3.1199226	0.0000000
H	2.5553947	-1.4321614	0.0000000
H	1.6601197	0.0764352	0.0000000
C	0.0954256	5.1241616	0.0000000
C	0.8524964	2.7466601	0.0000000
C	-0.5362541	2.3411232	0.0000000
C	-1.4935850	3.2917628	0.0000000
H	2.0132845	4.4449077	0.0000000
H	-2.5495219	3.0523338	0.0000000
H	-1.9221970	5.3290132	0.0000000
N	1.0506938	4.1258511	0.0000000
O	1.8218099	1.9934108	0.0000000
O	0.3572534	6.3104659	0.0000000
N	-1.1944402	4.6294477	0.0000000
H	-2.6973628	-1.2334239	0.0000000
H	-0.7694350	1.2825778	0.0000000
Br	1.8159173	-6.7934618	0.0000000

35 (U···IA)

C	0.5622565	-3.0595520	0.0000000
C	-0.6782248	-3.6967156	0.0000000
C	-1.7642598	-1.7898640	0.0000000
C	0.5478256	-1.6499025	0.0000000
C	0.9900248	-5.1284068	0.0000000
N	1.6043111	-3.9602311	0.0000000
N	-0.3751345	-5.0387294	0.0000000
H	-1.0316295	-5.8065303	0.0000000
N	1.6769323	-0.9301534	0.0000000
N	-0.6481331	-1.0379084	0.0000000
N	-1.8825046	-3.1175166	0.0000000
H	2.5562526	-1.4199307	0.0000000
H	1.6582792	0.0872996	0.0000000
C	0.0755757	5.1299295	0.0000000
C	0.8418577	2.7553759	0.0000000
C	-0.5453038	2.3445401	0.0000000
C	-1.5063274	3.2914320	0.0000000
H	1.9960612	4.4581604	0.0000000
H	-2.5613305	3.0478559	0.0000000
H	-1.9427383	5.3271184	0.0000000
N	1.0347292	4.1353403	0.0000000
O	1.8140028	2.0057640	0.0000000
O	0.3327956	6.3172678	0.0000000
N	-1.2123553	4.6302950	0.0000000
H	-2.6964855	-1.2331591	0.0000000
I	1.9278361	-6.9468423	0.0000000
H	-0.7743133	1.2850634	0.0000000

36 (FU···FA)

C	0.5250749	-3.3549305	0.0000000
C	-0.5169902	-4.2794142	0.0000000
C	-2.0426684	-2.7019479	0.0000000
C	0.1582368	-1.9989805	0.0000000

C	1.4446648	-5.2314097	0.0000000
N	1.7669413	-3.9733449	0.0000000
N	0.1144290	-5.5112856	0.0000000
H	-0.3167592	-6.4235670	0.0000000
N	1.0738732	-1.0167982	0.0000000
N	-1.1498671	-1.6994660	0.0000000
N	-1.8241283	-4.0207319	0.0000000
H	2.0498691	-1.2593710	0.0000000
H	0.8039677	-0.0427605	0.0000000
C	0.5584755	5.4665099	0.0000000
C	0.6487857	2.9643148	0.0000000
C	-0.8049450	2.9867979	0.0000000
C	-1.4835151	4.1483719	0.0000000
H	2.2168716	4.2828818	0.0000000
H	-2.5647490	4.1796993	0.0000000
H	-1.3228768	6.2216511	0.0000000
N	1.2033374	4.2378730	0.0000000
O	1.3408477	1.9590027	0.0000000
O	1.1453866	6.5290822	0.0000000
N	-0.8145986	5.3496706	0.0000000
H	-3.0837941	-2.3960106	0.0000000
F	2.3037991	-6.2299787	0.0000000
F	-1.4296688	1.8141420	0.0000000

37 (ClU···ClA)

C	0.5139570	-3.3491009	0.0000000
C	-0.5240602	-4.2798867	0.0000000
C	-2.0578091	-2.7102661	0.0000000
C	0.1403547	-1.9913734	0.0000000
C	1.4502152	-5.2359738	0.0000000
N	1.7524360	-3.9571861	0.0000000
N	0.1112108	-5.5025883	0.0000000
H	-0.3230592	-6.4141351	0.0000000
N	1.0565435	-1.0104199	0.0000000
N	-1.1693590	-1.7008815	0.0000000
N	-1.8344657	-4.0261364	0.0000000
H	2.0315384	-1.2589569	0.0000000
H	0.7950979	-0.0340063	0.0000000
C	0.5490749	5.5232543	0.0000000
C	0.6646347	3.0244099	0.0000000
C	-0.7950648	3.0143411	0.0000000
C	-1.4755539	4.1809340	0.0000000
H	2.2197930	4.3605368	0.0000000
H	-2.5573616	4.2153773	0.0000000
H	-1.3439440	6.2537177	0.0000000
N	1.2066531	4.3051971	0.0000000
O	1.3799962	2.0355257	0.0000000
O	1.1179914	6.5955311	0.0000000
N	-0.8246080	5.3876032	0.0000000
H	-3.0999617	-2.4070184	0.0000000
Cl	-1.5612141	1.4903922	0.0000000
Cl	2.5769644	-6.5088907	0.0000000

38 (BrU···BrA)

C	0.5079016	-3.3772189	0.0000000
C	-0.5202380	-4.3193403	0.0000000
C	-2.0712825	-2.7676925	0.0000000
C	0.1201761	-2.0227766	0.0000000
C	1.4641608	-5.2559168	0.0000000
N	1.7522320	-3.9720700	0.0000000
N	0.1271874	-5.5348611	0.0000000
H	-0.3007545	-6.4496425	0.0000000
N	1.0287558	-1.0343173	0.0000000
N	-1.1933492	-1.7483272	0.0000000
N	-1.8336820	-4.0801809	0.0000000
H	2.0053832	-1.2770971	0.0000000
H	0.7614621	-0.0597173	0.0000000
C	0.5633292	5.5808318	0.0000000
C	0.6569362	3.0802867	0.0000000

C	-0.8027811	3.0836239	0.0000000
C	-1.4729038	4.2562826	0.0000000
H	2.2237855	4.4054643	0.0000000
H	-2.5542851	4.3045336	0.0000000
H	-1.3233120	6.3283896	0.0000000
N	1.2102713	4.3577815	0.0000000
O	1.3674634	2.0876332	0.0000000
O	1.1405571	6.6487567	0.0000000
N	-0.8120469	5.4573872	0.0000000
H	-3.1168703	-2.4760703	0.0000000
Br	-1.6433030	1.4174497	0.0000000
Br	2.7152067	-6.6331921	0.0000000

39 (IU···IA)

C	0.5046342	-3.4280198	0.0000000
C	-0.5112447	-4.3839161	0.0000000
C	-2.0829550	-2.8527624	0.0000000
C	0.0997627	-2.0778851	0.0000000
C	1.4876586	-5.2980921	0.0000000
N	1.7552059	-4.0054884	0.0000000
N	0.1512883	-5.5896929	0.0000000
H	-0.2673342	-6.5091389	0.0000000
N	0.9987053	-1.0801970	0.0000000
N	-1.2176721	-1.8212757	0.0000000
N	-1.8282589	-4.1609889	0.0000000
H	1.9776292	-1.3143827	0.0000000
H	0.7216595	-0.1085658	0.0000000
C	0.5861659	5.6637400	0.0000000
C	0.6280954	3.1623285	0.0000000
C	-0.8318386	3.1898803	0.0000000
C	-1.4753012	4.3786922	0.0000000
H	2.2224712	4.4559563	0.0000000
H	-2.5553015	4.4566919	0.0000000
H	-1.2853126	6.4486507	0.0000000
N	1.2082794	4.4291059	0.0000000
O	1.3244663	2.1586761	0.0000000
O	1.1838805	6.7206728	0.0000000
N	-0.7916782	5.5672026	0.0000000
H	-3.1322228	-2.5747728	0.0000000
I	-1.7618756	1.3606388	0.0000000
I	2.8910931	-6.7870577	0.0000000

Binding mode d

40 (U···A)

C	-0.0473375	-2.3145463	0.0000000
C	-0.7025800	-3.5478128	0.0000000
C	1.1808690	-4.6733225	0.0000000
C	1.3635142	-2.3647628	0.0000000
C	-2.1263307	-1.8698566	0.0000000
N	-0.9474116	-1.2694730	0.0000000
N	-2.0398250	-3.2309685	0.0000000
H	-2.8007120	-3.8938735	0.0000000
N	2.1401357	-1.2724470	0.0000000
N	1.9488631	-3.5720057	0.0000000
N	-0.1495071	-4.7640366	0.0000000
H	1.7752218	-0.3268962	0.0000000
H	3.1366772	-1.4158866	0.0000000
C	0.2286415	4.8461162	0.0000000
C	0.8864477	2.4383845	0.0000000
C	-0.5185337	2.0913630	0.0000000
C	-1.4353089	3.0809674	0.0000000
H	2.1163929	4.0883983	0.0000000
H	-2.5005201	2.8866733	0.0000000
H	-1.7785676	5.1349618	0.0000000
N	1.1414364	3.8094229	0.0000000
O	1.8212862	1.6449187	0.0000000
O	0.5383735	6.0209012	0.0000000
N	-1.0809267	4.4054381	0.0000000

H	1.7219691	-5.6140930	0.0000000
H	-3.0789846	-1.3608093	0.0000000
H	-0.7932829	1.0432449	0.0000000

41 (FU···A)

C	-0.2738930	-2.7521887	0.0000000
C	-0.5922583	-4.1113536	0.0000000
C	1.5204059	-4.7123539	0.0000000
C	1.0999727	-2.4392136	0.0000000
C	-2.3916248	-2.8430783	0.0000000
N	-1.4040082	-1.9650607	0.0000000
N	-1.9658576	-4.1423859	0.0000000
H	-2.5365943	-4.9744521	0.0000000
N	1.5534463	-1.1757914	0.0000000
N	1.9806694	-3.4492030	0.0000000
N	0.2583311	-5.1429118	0.0000000
H	0.9342835	-0.3816440	0.0000000
H	2.5469617	-1.0183741	0.0000000
C	0.6132464	5.2625599	0.0000000
C	0.8761438	2.7716050	0.0000000
C	-0.5765905	2.6963989	0.0000000
C	-1.3342401	3.8074696	0.0000000
H	2.3496739	4.1975391	0.0000000
H	-2.4150801	3.7648684	0.0000000
H	-1.3152415	5.8873452	0.0000000
N	1.3417673	4.0819485	0.0000000
O	1.6298202	1.8131862	0.0000000
O	1.1244772	6.3637224	0.0000000
N	-0.7488932	5.0520059	0.0000000
F	-1.1185718	1.4819238	0.0000000
H	2.2853359	-5.4822823	0.0000000
H	-3.4416821	-2.5902795	0.0000000

42 (ClU···A)

C	-0.2805476	-2.7471964	0.0000000
C	-0.5936254	-4.1085782	0.0000000
C	1.5188128	-4.7072195	0.0000000
C	1.0948349	-2.4322846	0.0000000
C	-2.4017501	-2.8534531	0.0000000
N	-1.4190022	-1.9689760	0.0000000
N	-1.9670381	-4.1480394	0.0000000
H	-2.5311483	-4.9847900	0.0000000
N	1.5610395	-1.1736638	0.0000000
N	1.9750417	-3.4436845	0.0000000
N	0.2573942	-5.1390613	0.0000000
H	0.9577268	-0.3671200	0.0000000
H	2.5578339	-1.0353666	0.0000000
C	0.6302865	5.2923305	0.0000000
C	0.8846429	2.8025246	0.0000000
C	-0.5731917	2.7118532	0.0000000
C	-1.3173593	3.8388788	0.0000000
H	2.3623092	4.2248845	0.0000000
H	-2.3996302	3.8142746	0.0000000
H	-1.3000069	5.9164002	0.0000000
N	1.3539774	4.1133019	0.0000000
O	1.6518125	1.8550724	0.0000000
O	1.1381527	6.3950312	0.0000000
N	-0.7340269	5.0801629	0.0000000
Cl	-1.2584696	1.1474715	0.0000000
H	2.2852815	-5.4757980	0.0000000
H	-3.4533502	-2.6069547	0.0000000

43 (BrU···A)

C	-0.2841675	-2.7719076	0.0000000
C	-0.5967918	-4.1339908	0.0000000
C	1.5143631	-4.7338242	0.0000000
C	1.0925076	-2.4572535	0.0000000

C	-2.4082087	-2.8839098	0.0000000
N	-1.4264728	-1.9980222	0.0000000
N	-1.9701657	-4.1759160	0.0000000
H	-2.5325346	-5.0139208	0.0000000
N	1.5667853	-1.2013834	0.0000000
N	1.9704271	-3.4707668	0.0000000
N	0.2528075	-5.1650086	0.0000000
H	0.9695453	-0.3900009	0.0000000
H	2.5649112	-1.0718978	0.0000000
C	0.6322946	5.3409442	0.0000000
C	0.8944899	2.8513815	0.0000000
C	-0.5627761	2.7559657	0.0000000
C	-1.3097744	3.8811031	0.0000000
H	2.3680524	4.2800855	0.0000000
H	-2.3920818	3.8580334	0.0000000
H	-1.3008646	5.9589361	0.0000000
N	1.3600130	4.1650622	0.0000000
O	1.6694987	1.9096600	0.0000000
O	1.1351944	6.4460520	0.0000000
N	-0.7317984	5.1246173	0.0000000
Br	-1.2961563	1.0361212	0.0000000
H	2.2806526	-5.5023565	0.0000000
H	-3.4597499	-2.6378033	0.0000000

44 (IU···A)

C	-0.2897723	-2.8292531	0.0000000
C	-0.5945976	-4.1938781	0.0000000
C	1.5178866	-4.7857622	0.0000000
C	1.0874306	-2.5091328	0.0000000
C	-2.4173837	-2.9608785	0.0000000
N	-1.4417604	-2.0673446	0.0000000
N	-1.9674922	-4.2469347	0.0000000
H	-2.5212922	-5.0908154	0.0000000
N	1.5677520	-1.2553974	0.0000000
N	1.9672815	-3.5212088	0.0000000
N	0.2581387	-5.2215445	0.0000000
H	0.9753630	-0.4400771	0.0000000
H	2.5673066	-1.1347167	0.0000000
C	0.6490095	5.4281862	0.0000000
C	0.8869020	2.9369566	0.0000000
C	-0.5710123	2.8482912	0.0000000
C	-1.3044262	3.9835437	0.0000000
H	2.3749208	4.3526524	0.0000000
H	-2.3873059	3.9779552	0.0000000
H	-1.2788955	6.0621931	0.0000000
N	1.3659026	4.2468501	0.0000000
O	1.6599266	1.9921889	0.0000000
O	1.1609397	6.5296102	0.0000000
N	-0.7168829	5.2229658	0.0000000
I	-1.3646283	0.9491622	0.0000000
H	2.2874360	-5.5509629	0.0000000
H	-3.4707466	-2.7226490	0.0000000

45 (U···FA)

C	-0.0260033	-2.3284068	0.0000000
C	-0.6840336	-3.5585594	0.0000000
C	1.1950333	-4.6870600	0.0000000
C	1.3802431	-2.3774485	0.0000000
C	-2.0735966	-1.8809837	0.0000000
N	-0.9269380	-1.2699584	0.0000000
N	-2.0309475	-3.2358038	0.0000000
H	-2.8130451	-3.8737152	0.0000000
N	2.1591830	-1.2861595	0.0000000
N	1.9625729	-3.5888220	0.0000000
N	-0.1386848	-4.7729281	0.0000000
H	1.7947518	-0.3397978	0.0000000
H	3.1553643	-1.4314644	0.0000000
C	0.2400410	4.8451582	0.0000000
C	0.8687898	2.4297847	0.0000000

C	-0.5400226	2.1014735	0.0000000
C	-1.4456691	3.1012362	0.0000000
H	2.1185678	4.0645724	0.0000000
H	-2.5127567	2.9175942	0.0000000
H	-1.7637770	5.1585842	0.0000000
N	1.1403869	3.7971711	0.0000000
O	1.7941412	1.6247272	0.0000000
O	0.5647544	6.0157970	0.0000000
N	-1.0749139	4.4207328	0.0000000
H	1.7317226	-5.6300565	0.0000000
H	-0.8297861	1.0590373	0.0000000
F	-3.2453778	-1.2747047	0.0000000

46 (U···ClA)

C	0.0042401	-2.3224986	0.0000000
C	-0.6602425	-3.5503978	0.0000000
C	1.2155747	-4.6856440	0.0000000
C	1.4133001	-2.3774035	0.0000000
C	-2.0568478	-1.8613505	0.0000000
N	-0.8856198	-1.2653534	0.0000000
N	-1.9997876	-3.2219646	0.0000000
H	-2.7807183	-3.8624536	0.0000000
N	2.1922594	-1.2875351	0.0000000
N	1.9897449	-3.5900598	0.0000000
N	-0.1173621	-4.7680165	0.0000000
H	1.8266634	-0.3410207	0.0000000
H	3.1886832	-1.4325149	0.0000000
C	0.2199631	4.8251772	0.0000000
C	0.8714683	2.4158532	0.0000000
C	-0.5339831	2.0739807	0.0000000
C	-1.4489603	3.0651826	0.0000000
H	2.1057804	4.0624072	0.0000000
H	-2.5142118	2.8709492	0.0000000
H	-1.7867723	5.1195239	0.0000000
N	1.1301842	3.7857244	0.0000000
O	1.8049880	1.6198814	0.0000000
O	0.5338504	5.9988795	0.0000000
N	-1.0908595	4.3883373	0.0000000
H	1.7492870	-5.6304623	0.0000000
H	-0.8131354	1.0283097	0.0000000
Cl	-3.5574867	-1.0575309	0.0000000

47 (U···BrA)

C	0.0167525	-2.3215100	0.0000000
C	-0.6482188	-3.5493053	0.0000000
C	1.2277275	-4.6845911	0.0000000
C	1.4262936	-2.3765039	0.0000000
C	-2.0455052	-1.8594589	0.0000000
N	-0.8722798	-1.2642811	0.0000000
N	-1.9875303	-3.2204678	0.0000000
H	-2.7674242	-3.8624916	0.0000000
N	2.2044516	-1.2864353	0.0000000
N	2.0023790	-3.5890442	0.0000000
N	-0.1050541	-4.7671272	0.0000000
H	1.8379928	-0.3401227	0.0000000
H	3.2009769	-1.4309909	0.0000000
C	0.2088825	4.8186808	0.0000000
C	0.8729560	2.4130613	0.0000000
C	-0.5306119	2.0640572	0.0000000
C	-1.4510424	3.0500488	0.0000000
H	2.0987853	4.0664320	0.0000000
H	-2.5151678	2.8492469	0.0000000
H	-1.7988242	5.1031427	0.0000000
N	1.1247779	3.7841748	0.0000000
O	1.8102038	1.6213471	0.0000000
O	0.5165375	5.9940482	0.0000000
N	-1.0996338	4.3750804	0.0000000
H	1.7615492	-5.6293725	0.0000000
H	-0.8037980	1.0168841	0.0000000

Br	-3.6851756	-0.9745018	0.0000000

48 (U···IA)

C	0.0170835	-2.3191661	0.0000000
C	-0.6609305	-3.5400068	0.0000000
C	1.2039042	-4.6941885	0.0000000
C	1.4265963	-2.3885629	0.0000000
C	-2.0446661	-1.8336559	0.0000000
N	-0.8594791	-1.2539082	0.0000000
N	-1.9958067	-3.1972350	0.0000000
H	-2.7796238	-3.8347236	0.0000000
N	2.2140318	-1.3052917	0.0000000
N	1.9903564	-3.6064653	0.0000000
N	-0.1294436	-4.7635138	0.0000000
H	1.8544264	-0.3562483	0.0000000
H	3.2094187	-1.4575843	0.0000000
C	0.2285180	4.8072041	0.0000000
C	0.8905303	2.4008354	0.0000000
C	-0.5133934	2.0533875	0.0000000
C	-1.4329545	3.0401771	0.0000000
H	2.1178058	4.0530268	0.0000000
H	-2.4972409	2.8399778	0.0000000
H	-1.7790592	5.0935258	0.0000000
N	1.1434961	3.7718300	0.0000000
O	1.8271911	1.6083527	0.0000000
O	0.5372221	5.9823279	0.0000000
N	-1.0804517	4.3649106	0.0000000
H	1.7281767	-5.6443053	0.0000000
I	-3.8288115	-0.8268559	0.0000000
H	-0.7868965	1.0061560	0.0000000

49 (FU···FA)

C	-0.2608327	-2.7359373	0.0000000
C	-0.5852212	-4.0926034	0.0000000
C	1.5215017	-4.7046072	0.0000000
C	1.1100662	-2.4283718	0.0000000
C	-2.3496998	-2.8134369	0.0000000
N	-1.3941342	-1.9345429	0.0000000
N	-1.9686709	-4.1178564	0.0000000
H	-2.5672478	-4.9301735	0.0000000
N	1.5760019	-1.1694531	0.0000000
N	1.9853148	-3.4463693	0.0000000
N	0.2538628	-5.1271587	0.0000000
H	0.9702697	-0.3648587	0.0000000
H	2.5715081	-1.0249403	0.0000000
C	0.6274936	5.2352938	0.0000000
C	0.8735822	2.7434675	0.0000000
C	-0.5791966	2.6769241	0.0000000
C	-1.3296330	3.7929505	0.0000000
H	2.3569255	4.1581997	0.0000000
H	-2.4107631	3.7572940	0.0000000
H	-1.2969692	5.8725464	0.0000000
N	1.3482447	4.0497086	0.0000000
O	1.6217226	1.7802007	0.0000000
O	1.1465331	6.3326444	0.0000000
N	-0.7360175	5.0334798	0.0000000
H	2.2805855	-5.4797757	0.0000000
F	-3.6366932	-2.5283521	0.0000000
F	-1.1285332	1.4657280	0.0000000

50 (ClU···ClA)

C	-0.2456430	-2.7420872	0.0000000
C	-0.5730006	-4.0999845	0.0000000
C	1.5315974	-4.7182557	0.0000000
C	1.1307000	-2.4379855	0.0000000

C	-2.3547595	-2.8226293	0.0000000
N	-1.3755889	-1.9465751	0.0000000
N	-1.9508219	-4.1246891	0.0000000
H	-2.5424320	-4.9430462	0.0000000
N	1.6080230	-1.1841174	0.0000000
N	1.9994837	-3.4605338	0.0000000
N	0.2641966	-5.1378458	0.0000000
H	1.0130580	-0.3707019	0.0000000
H	2.6060923	-1.0544928	0.0000000
C	0.6415350	5.2751655	0.0000000
C	0.9015351	2.7861921	0.0000000
C	-0.5557179	2.6928278	0.0000000
C	-1.3023684	3.8180989	0.0000000
H	2.3763366	4.2113028	0.0000000
H	-2.3844103	3.7903149	0.0000000
H	-1.2905688	5.8951381	0.0000000
N	1.3681870	4.0974494	0.0000000
O	1.6710271	1.8400500	0.0000000
O	1.1469231	6.3788508	0.0000000
N	-0.7222299	5.0604174	0.0000000
H	2.2894225	-5.4950441	0.0000000
Cl	-4.0108798	-2.4361899	0.0000000
Cl	-1.2396964	1.1283704	0.0000000

51 (BrU···BrA)

C	-0.2468874	-2.7775989	0.0000000
C	-0.5629620	-4.1389628	0.0000000
C	1.5457625	-4.7411177	0.0000000
C	1.1286623	-2.4625766	0.0000000
C	-2.3596860	-2.8811671	0.0000000
N	-1.3866414	-1.9956046	0.0000000
N	-1.9404429	-4.1777868	0.0000000
H	-2.5217529	-5.0039548	0.0000000
N	1.6029240	-1.2075874	0.0000000
N	2.0034769	-3.4799353	0.0000000
N	0.2818811	-5.1704650	0.0000000
H	1.0066505	-0.3947533	0.0000000
H	2.6013307	-1.0786754	0.0000000
C	0.6622208	5.3265384	0.0000000
C	0.9071988	2.8354199	0.0000000
C	-0.5504172	2.7503835	0.0000000
C	-1.2898948	3.8806092	0.0000000
H	2.3905549	4.2542429	0.0000000
H	-2.3723117	3.8642699	0.0000000
H	-1.2661106	5.9584655	0.0000000
N	1.3818563	4.1454014	0.0000000
O	1.6757758	1.8880286	0.0000000
O	1.1729992	6.4279236	0.0000000
N	-0.7032984	5.1198299	0.0000000
H	2.3097701	-5.5117014	0.0000000
Br	-4.1736194	-2.4660813	0.0000000
Br	-1.2970390	1.0368557	0.0000000

52 (IU···IA)

C	-0.2447122	-2.8351972	0.0000000
C	-0.5475873	-4.2004586	0.0000000
C	1.5663203	-4.7841051	0.0000000
C	1.1301750	-2.5081429	0.0000000
C	-2.3655061	-2.9652420	0.0000000
N	-1.3949893	-2.0698961	0.0000000
N	-1.9235699	-4.2551989	0.0000000
H	-2.4899767	-5.0919180	0.0000000
N	1.6013887	-1.2519646	0.0000000
N	2.0122716	-3.5189231	0.0000000
N	0.3065498	-5.2244000	0.0000000
H	1.0034539	-0.4399180	0.0000000
H	2.6001272	-1.1235092	0.0000000
C	0.6935760	5.4018040	0.0000000
C	0.8945985	2.9078146	0.0000000

C	-0.5641990	2.8408366	0.0000000
C	-1.2811975	3.9866708	0.0000000
H	2.4035267	4.3007623	0.0000000
H	-2.3640474	3.9967681	0.0000000
H	-1.2241471	6.0650024	0.0000000
N	1.3930183	4.2098134	0.0000000
O	1.6533242	1.9511753	0.0000000
O	1.2219925	6.4952446	0.0000000
N	-0.6752696	5.2170158	0.0000000
H	2.3374575	-5.5475066	0.0000000
I	-1.3833795	0.9536339	0.0000000
I	-4.3591982	-2.5101615	0.0000000

Binding mode a

53 (C···G)

C	1.0373834	-2.9498286	0.0000000
C	-0.2202566	-3.5502259	0.0000000
C	-1.3710624	-1.6660649	0.0000000
C	1.0927390	-1.5236462	0.0000000
C	1.4048496	-5.0385410	0.0000000
N	2.0448364	-3.8869253	0.0000000
N	0.0394019	-4.8926387	0.0000000
H	-0.6578366	-5.6218375	0.0000000
N	-0.1899108	-0.9713849	0.0000000
N	-1.4439621	-2.9814719	0.0000000
C	-1.3678365	2.5682089	0.0000000
C	-0.2274459	4.6986091	0.0000000
C	0.9768218	4.0809772	0.0000000
C	0.9708497	2.6455754	0.0000000
N	-1.3671497	3.9649521	0.0000000
O	-2.4491978	1.9773463	0.0000000
N	-0.1619440	1.9441061	0.0000000
N	-2.4968673	-0.9258067	0.0000000
H	-2.4873101	0.0931844	0.0000000
H	-3.3663632	-1.4291172	0.0000000
O	2.0891728	-0.7912618	0.0000000
H	-0.3422424	5.7751661	0.0000000
N	2.1180820	1.9706591	0.0000000
H	2.9905596	2.4704645	0.0000000
H	2.1148840	0.9348938	0.0000000
H	-0.2223586	0.0637524	0.0000000
H	-2.2776133	4.4031006	0.0000000
H	1.8739732	-6.0109078	0.0000000
H	1.8958040	4.6486622	0.0000000

54 (FC···G)

C	1.0317054	-2.9503028	0.0000000
C	-0.2256114	-3.5526607	0.0000000
C	-1.3803195	-1.6715930	0.0000000
C	1.0844670	-1.5241832	0.0000000
C	1.4008939	-5.0387380	0.0000000
N	2.0398547	-3.8866376	0.0000000
N	0.0352597	-4.8943020	0.0000000
H	-0.6617472	-5.6238053	0.0000000
N	-0.1996786	-0.9758514	0.0000000
N	-1.4506037	-2.9866331	0.0000000
C	-1.3874696	2.5708378	0.0000000
C	-0.2353284	4.7009378	0.0000000
C	0.9460932	4.0529419	0.0000000
C	0.9608624	2.6193484	0.0000000
N	-1.3805277	3.9632049	0.0000000
O	-2.4670001	1.9771262	0.0000000
N	-0.1799698	1.9394379	0.0000000
N	-2.5068679	-0.9322152	0.0000000
H	-2.4990460	0.0862702	0.0000000
H	-3.3762521	-1.4357253	0.0000000
O	2.0782907	-0.7872801	0.0000000
H	-0.3144883	5.7796946	0.0000000

N	2.1185431	1.9703484	0.0000000
H	2.9770738	2.4959367	0.0000000
H	2.1283210	0.9325508	0.0000000
H	-0.2349640	0.0574276	0.0000000
H	-2.2870099	4.4094847	0.0000000
F	2.1146122	4.7149336	0.0000000
H	1.8709069	-6.0105537	0.0000000

55 (ClC···G)

C	1.0198107	-2.9592356	0.0000000
C	-0.2376877	-3.5608066	0.0000000
C	-1.3912098	-1.6789087	0.0000000
C	1.0732877	-1.5333139	0.0000000
C	1.3881380	-5.0476709	0.0000000
N	2.0276681	-3.8958432	0.0000000
N	0.0226360	-4.9026244	0.0000000
H	-0.6746718	-5.6318583	0.0000000
N	-0.2102874	-0.9834313	0.0000000
N	-1.4621965	-2.9938462	0.0000000
C	-1.3909754	2.5598688	0.0000000
C	-0.2494338	4.6908258	0.0000000
C	0.9510620	4.0642312	0.0000000
C	0.9584905	2.6223018	0.0000000
N	-1.3882472	3.9556283	0.0000000
O	-2.4708863	1.9678443	0.0000000
N	-0.1835009	1.9379418	0.0000000
N	-2.5174383	-0.9391166	0.0000000
H	-2.5083460	0.0792766	0.0000000
H	-3.3870767	-1.4422013	0.0000000
O	2.0678708	-0.7976179	0.0000000
H	-0.3460653	5.7689188	0.0000000
N	2.1030820	1.9540921	0.0000000
H	2.9717994	2.4630867	0.0000000
H	2.0969695	0.9157493	0.0000000
H	-0.2442075	0.0500672	0.0000000
H	-2.2963512	4.3999456	0.0000000
Cl	2.4204190	4.9566106	0.0000000
H	1.8573482	-6.0199141	0.0000000

56 (BrC···G)

C	1.0146886	-2.9634915	0.0000000
C	-0.2430197	-3.5646175	0.0000000
C	-1.3960746	-1.6824894	0.0000000
C	1.0685829	-1.5375446	0.0000000
C	1.3822055	-5.0521095	0.0000000
N	2.0221750	-3.9005015	0.0000000
N	0.0167590	-4.9065079	0.0000000
H	-0.6809893	-5.6353128	0.0000000
N	-0.2149155	-0.9872674	0.0000000
N	-1.4673550	-2.9973807	0.0000000
C	-1.3915303	2.5556115	0.0000000
C	-0.2553283	4.6885182	0.0000000
C	0.9482612	4.0658632	0.0000000
C	0.9593303	2.6233743	0.0000000
N	-1.3923501	3.9518947	0.0000000
O	-2.4704596	1.9619174	0.0000000
N	-0.1830358	1.9372492	0.0000000
N	-2.5220752	-0.9425001	0.0000000
H	-2.5122400	0.0758687	0.0000000
H	-3.3919182	-1.4452360	0.0000000
O	2.0632162	-0.8019653	0.0000000
H	-0.3595422	5.7660152	0.0000000
N	2.1005456	1.9496292	0.0000000
H	2.9730934	2.4526009	0.0000000
H	2.0892596	0.9111804	0.0000000
H	-0.2480674	0.0461705	0.0000000
H	-2.3015793	4.3942916	0.0000000
Br	2.5413535	5.0612941	0.0000000
H	1.8510094	-6.0245548	0.0000000

57 (IC···G)

C	1.0092478	-2.9681939	0.0000000
C	-0.2486238	-3.5687644	0.0000000
C	-1.4008842	-1.6859291	0.0000000
C	1.0636167	-1.5422896	0.0000000
C	1.3757485	-5.0570837	0.0000000
N	2.0162295	-3.9058278	0.0000000
N	0.0103088	-4.9108701	0.0000000
H	-0.6877327	-5.6392913	0.0000000
N	-0.2194547	-0.9911524	0.0000000
N	-1.4726048	-3.0008133	0.0000000
C	-1.3937542	2.5505317	0.0000000
C	-0.2610733	4.6841704	0.0000000
C	0.9483267	4.0685789	0.0000000
C	0.9576632	2.6246756	0.0000000
N	-1.3967819	3.9469283	0.0000000
O	-2.4720415	1.9556281	0.0000000
N	-0.1844948	1.9356813	0.0000000
I	2.6822997	5.1846861	0.0000000
N	-2.5265908	-0.9457310	0.0000000
H	-2.5163159	0.0726413	0.0000000
H	-3.3966457	-1.4480718	0.0000000
O	2.0585921	-0.8071910	0.0000000
H	-0.3751869	5.7609416	0.0000000
N	2.0946927	1.9433440	0.0000000
H	2.9722205	2.4379265	0.0000000
H	2.0777507	0.9047579	0.0000000
H	-0.2519544	0.0423690	0.0000000
H	-2.3067961	4.3881121	0.0000000
H	1.8442387	-6.0297634	0.0000000

58 (C···FG)

C	1.0342308	-2.9395517	0.0000000
C	-0.2220896	-3.5371843	0.0000000
C	-1.3715140	-1.6548759	0.0000000
C	1.0928659	-1.5173723	0.0000000
C	1.3962450	-4.9988730	0.0000000
N	2.0518696	-3.8834543	0.0000000
N	0.0363271	-4.8898057	0.0000000
H	-0.6400068	-5.6383950	0.0000000
N	-0.1911747	-0.9636739	0.0000000
N	-1.4429679	-2.9727421	0.0000000
C	-1.3733809	2.5743104	0.0000000
C	-0.2318942	4.7039157	0.0000000
C	0.9717989	4.0855247	0.0000000
C	0.9653882	2.6504132	0.0000000
N	-1.3719368	3.9707361	0.0000000
O	-2.4549187	1.9836497	0.0000000
N	-0.1677965	1.9490394	0.0000000
N	-2.4983541	-0.9180041	0.0000000
H	-2.4901833	0.1015053	0.0000000
H	-3.3671480	-1.4227077	0.0000000
O	2.0905225	-0.7862012	0.0000000
H	-0.3448783	5.7805570	0.0000000
N	2.1127714	1.9761995	0.0000000
H	2.9847665	2.4767586	0.0000000
H	2.1116016	0.9408275	0.0000000
H	-0.2223299	0.0725050	0.0000000
H	-2.2824785	4.4087218	0.0000000
F	1.9336364	-6.2045898	0.0000000
H	1.8910283	4.6527674	0.0000000

59 (C···ClG)

C	1.0275253	-2.9306294	0.0000000
C	-0.2307950	-3.5282460	0.0000000
C	-1.3770778	-1.6430236	0.0000000
C	1.0872898	-1.5052599	0.0000000
C	1.3908035	-5.0062077	0.0000000

N	2.0392322	-3.8690236	0.0000000
N	0.0267732	-4.8751706	0.0000000
H	-0.6550216	-5.6197534	0.0000000
N	-0.1954077	-0.9513146	0.0000000
N	-1.4512747	-2.9602422	0.0000000
C	-1.3770892	2.5852257	0.0000000
C	-0.2396071	4.7164860	0.0000000
C	0.9654524	4.1006139	0.0000000
C	0.9617159	2.6655228	0.0000000
N	-1.3782867	3.9814848	0.0000000
O	-2.4574517	1.9924369	0.0000000
N	-0.1702693	1.9623351	0.0000000
N	-2.5017864	-0.9038727	0.0000000
H	-2.4918387	0.1157794	0.0000000
H	-3.3716365	-1.4069297	0.0000000
O	2.0854724	-0.7765035	0.0000000
H	-0.3549794	5.7928277	0.0000000
N	2.1100750	1.9924067	0.0000000
H	2.9820352	2.4930192	0.0000000
H	2.1094049	0.9577041	0.0000000
H	-0.2263777	0.0847923	0.0000000
H	-2.2892935	4.4185876	0.0000000
Cl	2.0995150	-6.5537355	0.0000000
H	1.8828984	4.6706902	0.0000000

60 (C···BrG)

C	1.0240421	-2.9246732	0.0000000
C	-0.2338700	-3.5238310	0.0000000
C	-1.3814874	-1.6390853	0.0000000
C	1.0824869	-1.4987153	0.0000000
C	1.3899025	-5.0010305	0.0000000
N	2.0367302	-3.8611951	0.0000000
N	0.0254793	-4.8703370	0.0000000
H	-0.6563142	-5.6151745	0.0000000
N	-0.2003968	-0.9458744	0.0000000
N	-1.4547081	-2.9564090	0.0000000
C	-1.3787120	2.5911589	0.0000000
C	-0.2395505	4.7221684	0.0000000
C	0.9650419	4.1053172	0.0000000
C	0.9598314	2.6701400	0.0000000
N	-1.3788418	3.9876641	0.0000000
O	-2.4595187	1.9990332	0.0000000
N	-0.1724537	1.9672213	0.0000000
N	-2.5069091	-0.9011639	0.0000000
H	-2.4983411	0.1184732	0.0000000
H	-3.3759531	-1.4057041	0.0000000
O	2.0805175	-0.7702040	0.0000000
H	-0.3552349	5.7986393	0.0000000
N	2.1077141	1.9965316	0.0000000
H	2.9798604	2.4967480	0.0000000
H	2.1053119	0.9619046	0.0000000
H	-0.2320291	0.0904135	0.0000000
H	-2.2893573	4.4258439	0.0000000
Br	2.1726765	-6.6910207	0.0000000
H	1.8840830	4.6731607	0.0000000

61 (C···IG)

C	1.0209528	-2.9205326	0.0000000
C	-0.2377961	-3.5190594	0.0000000
C	-1.3842132	-1.6332625	0.0000000
C	1.0798101	-1.4937298	0.0000000
C	1.3863632	-5.0019775	0.0000000
N	2.0319196	-3.8567128	0.0000000
N	0.0204735	-4.8646828	0.0000000
H	-0.6653868	-5.6060719	0.0000000
N	-0.2026015	-0.9404603	0.0000000
N	-1.4583768	-2.9503245	0.0000000
C	-1.3815095	2.5963036	0.0000000
C	-0.2435228	4.7279410	0.0000000

C	0.9614346	4.1117208	0.0000000
C	0.9572693	2.6766126	0.0000000
N	-1.3823356	3.9927684	0.0000000
O	-2.4618723	2.0034493	0.0000000
N	-0.1746245	1.9732388	0.0000000
I	2.2583504	-6.8542218	0.0000000
N	-2.5089048	-0.8942206	0.0000000
H	-2.4994042	0.1254086	0.0000000
H	-3.3783843	-1.3980386	0.0000000
O	2.0777826	-0.7654004	0.0000000
H	-0.3592416	5.8044286	0.0000000
N	2.1062604	2.0044179	0.0000000
H	2.9774939	2.5064534	0.0000000
H	2.1065973	0.9698871	0.0000000
H	-0.2335586	0.0956722	0.0000000
H	-2.2931181	4.4303870	0.0000000
H	1.8801428	4.6800065	0.0000000

62 (FC···FG)

C	1.0270470	-2.9422415	0.0000000
C	-0.2292501	-3.5413726	0.0000000
C	-1.3819538	-1.6615850	0.0000000
C	1.0836731	-1.5196660	0.0000000
C	1.3900678	-5.0017929	0.0000000
N	2.0449243	-3.8857964	0.0000000
N	0.0301049	-4.8934842	0.0000000
H	-0.6456742	-5.6427487	0.0000000
N	-0.2019227	-0.9696205	0.0000000
N	-1.4513405	-2.9790797	0.0000000
C	-1.3900340	2.5767819	0.0000000
C	-0.2397838	4.7080362	0.0000000
C	0.9420019	4.0607750	0.0000000
C	0.9579347	2.6272667	0.0000000
N	-1.3841451	3.9689239	0.0000000
O	-2.4689365	1.9818991	0.0000000
N	-0.1820332	1.9458100	0.0000000
N	-2.5093068	-0.9251481	0.0000000
H	-2.5025282	0.0939366	0.0000000
H	-3.3779260	-1.4301142	0.0000000
O	2.0781480	-0.7834908	0.0000000
H	-0.3198629	5.7867390	0.0000000
N	2.1165297	1.9800384	0.0000000
H	2.9741492	2.5072637	0.0000000
H	2.1282625	0.9430491	0.0000000
H	-0.2350132	0.0646071	0.0000000
H	-2.2908885	4.4146442	0.0000000
F	2.1099712	4.7232665	0.0000000
F	1.9277850	-6.2068968	0.0000000

63 (ClC···ClG)

C	1.0093209	-2.9391753	0.0000000
C	-0.2484044	-3.5389572	0.0000000
C	-1.3987630	-1.6564159	0.0000000
C	1.0660567	-1.5140820	0.0000000
C	1.3756258	-5.0142750	0.0000000
N	2.0222800	-3.8759998	0.0000000
N	0.0112466	-4.8851369	0.0000000
H	-0.6700280	-5.6302903	0.0000000
N	-0.2178498	-0.9631651	0.0000000
N	-1.4700593	-2.9733239	0.0000000
C	-1.3999746	2.5779281	0.0000000
C	-0.2594189	4.7093029	0.0000000
C	0.9415068	4.0835532	0.0000000
C	0.9494425	2.6418727	0.0000000
N	-1.3979439	3.9734129	0.0000000
O	-2.4790911	1.9842948	0.0000000
N	-0.1920450	1.9564829	0.0000000
N	-2.5247606	-0.9189266	0.0000000
H	-2.5169198	0.1001077	0.0000000

H	-3.3938731	-1.4232975	0.0000000
O	2.0620069	-0.7815024	0.0000000
H	-0.3568892	5.7873225	0.0000000
N	2.0945191	1.9745323	0.0000000
H	2.9630084	2.4838459	0.0000000
H	2.0896411	0.9372098	0.0000000
H	-0.2504650	0.0713125	0.0000000
H	-2.3059387	4.4181212	0.0000000
Cl	2.0869159	-6.5602061	0.0000000
Cl	2.4108537	4.9754545	0.0000000

64 (BrC···BrG)

C	1.0016279	-2.9387418	0.0000000
C	-0.2566264	-3.5381292	0.0000000
C	-1.4059814	-1.6547704	0.0000000
C	1.0589856	-1.5130443	0.0000000
C	1.3670524	-5.0154525	0.0000000
N	2.0139898	-3.8755503	0.0000000
N	0.0025187	-4.8842277	0.0000000
H	-0.6803074	-5.6282056	0.0000000
N	-0.2246137	-0.9616670	0.0000000
N	-1.4779484	-2.9715867	0.0000000
C	-1.4029532	2.5784857	0.0000000
C	-0.2678356	4.7117213	0.0000000
C	0.9363361	4.0902381	0.0000000
C	0.9479903	2.6480400	0.0000000
N	-1.4046195	3.9744752	0.0000000
O	-2.4808844	1.9827605	0.0000000
N	-0.1938505	1.9608476	0.0000000
N	-2.5314998	-0.9169426	0.0000000
H	-2.5230558	0.1021314	0.0000000
H	-3.4008307	-1.4209911	0.0000000
O	2.0551771	-0.7812837	0.0000000
H	-0.3729890	5.7890968	0.0000000
N	2.0897319	1.9751578	0.0000000
H	2.9620346	2.4784809	0.0000000
H	2.0796939	0.9378576	0.0000000
H	-0.2565437	0.0727835	0.0000000
H	-2.3135572	4.4176864	0.0000000
Br	2.1499071	-6.7051107	0.0000000
Br	2.5290514	5.0859407	0.0000000

65 (IC···IG)

C	0.9931804	-2.9381762	0.0000000
C	-0.2656538	-3.5371206	0.0000000
C	-1.4137727	-1.6525585	0.0000000
C	1.0507992	-1.5117745	0.0000000
C	1.3586435	-5.0197146	0.0000000
N	2.0041479	-3.8743525	0.0000000
N	-0.0073486	-4.8823347	0.0000000
H	-0.6935467	-5.6234572	0.0000000
N	-0.2320484	-0.9593880	0.0000000
N	-1.4865683	-2.9691535	0.0000000
C	-1.4082894	2.5795022	0.0000000
C	-0.2762989	4.7133587	0.0000000
C	0.9334906	4.0984688	0.0000000
C	0.9431090	2.6548454	0.0000000
N	-1.4118358	3.9756123	0.0000000
O	-2.4859482	1.9830468	0.0000000
N	-0.1985897	1.9649043	0.0000000
I	2.6669976	5.2151364	0.0000000
I	2.2304602	-6.8718343	0.0000000
N	-2.5388867	-0.9143217	0.0000000
H	-2.5299173	0.1047630	0.0000000
H	-3.4082328	-1.4183194	0.0000000
O	2.0476193	-0.7811252	0.0000000
H	-0.3908621	5.7901092	0.0000000
N	2.0808395	1.9744431	0.0000000
H	2.9580531	2.4696246	0.0000000

H	2.0654788	0.9370511	0.0000000
H	-0.2634011	0.0752689	0.0000000
H	-2.3216184	4.4174961	0.0000000

Binding mode b

66 (C···G)

C	1.3493321	-1.4300089	0.0000000
C	0.6750873	-2.6464359	0.0000000
C	2.4848327	-3.9111776	0.0000000
C	2.7818100	-1.4329799	0.0000000
C	-0.7291116	-0.9440475	0.0000000
N	0.4672517	-0.3791853	0.0000000
N	-0.6482926	-2.3100313	0.0000000
H	-1.4220298	-2.9584980	0.0000000
N	3.2496844	-2.7758845	0.0000000
N	1.1781170	-3.9066899	0.0000000
C	-3.3263069	2.2169308	0.0000000
C	-2.7643565	4.5811380	0.0000000
C	-1.4417826	4.2902013	0.0000000
C	-1.0910340	2.8964654	0.0000000
H	-4.6688336	3.7747019	0.0000000
N	-3.6773942	3.5818341	0.0000000
O	-4.2192336	1.3787747	0.0000000
N	-1.9936731	1.9277884	0.0000000
N	3.1485478	-5.0976273	0.0000000
H	4.1488977	-5.1589326	0.0000000
H	2.5963541	-5.9366574	0.0000000
O	3.5792068	-0.5105248	0.0000000
H	-3.1486988	5.5937961	0.0000000
N	0.2009830	2.5404700	0.0000000
H	0.9206521	3.2412764	0.0000000
H	0.4456958	1.5430068	0.0000000
H	4.2604437	-2.8473724	0.0000000
H	-1.6598302	-0.3926033	0.0000000
H	-0.6963187	5.0722726	0.0000000

67 (FC···G)

C	-0.0598969	-2.0162866	0.0000000
C	-0.3386200	-3.3794998	0.0000000
C	1.7645364	-4.0482109	0.0000000
C	1.3108828	-1.5993850	0.0000000
C	-2.1800675	-2.1722355	0.0000000
N	-1.2154530	-1.2726031	0.0000000
N	-1.7020039	-3.4559495	0.0000000
H	-2.2415822	-4.3093470	0.0000000
N	2.1550750	-2.7359417	0.0000000
N	0.5144224	-4.4319736	0.0000000
C	-2.2743873	4.5826760	0.0000000
C	0.0924325	5.1816610	0.0000000
C	0.4106537	3.8729092	0.0000000
C	-0.6455238	2.9008365	0.0000000
H	-1.5098952	6.4908933	0.0000000
N	-1.2244867	5.5224514	0.0000000
O	-3.4259813	4.9928729	0.0000000
N	-1.9148665	3.2599039	0.0000000
N	2.7503953	-4.9800665	0.0000000
H	3.7238638	-4.7398007	0.0000000
H	2.4750255	-5.9462836	0.0000000
O	1.8062225	-0.4817133	0.0000000
H	0.8356315	5.9680155	0.0000000
N	-0.3146853	1.6073219	0.0000000
H	0.6442136	1.2904078	0.0000000
H	-1.0335712	0.8935368	0.0000000
H	3.1413426	-2.5017469	0.0000000
F	1.6926047	3.4581202	0.0000000
H	-3.2362815	-1.9505626	0.0000000

68 (ClC···G)

C	-0.0807455	-2.0154841	0.0000000
C	-0.3541217	-3.3799559	0.0000000
C	1.7513981	-4.0411003	0.0000000
C	1.2886797	-1.5940016	0.0000000
C	-2.2001171	-2.1798007	0.0000000
N	-1.2392101	-1.2761575	0.0000000
N	-1.7171154	-3.4617121	0.0000000
H	-2.2532948	-4.3172926	0.0000000
N	2.1368403	-2.7274313	0.0000000
N	0.5027053	-4.4293949	0.0000000
C	-2.2821557	4.5734344	0.0000000
C	0.0763664	5.1883811	0.0000000
C	0.4270207	3.8807458	0.0000000
C	-0.6374521	2.9053662	0.0000000
H	-1.5256950	6.4894792	0.0000000
N	-1.2349043	5.5219755	0.0000000
O	-3.4340913	4.9811876	0.0000000
N	-1.9108572	3.2591401	0.0000000
N	2.7410671	-4.9690246	0.0000000
H	3.7135358	-4.7246555	0.0000000
H	2.4699247	-5.9363852	0.0000000
O	1.7808486	-0.4747403	0.0000000
H	0.8050204	5.9893833	0.0000000
N	-0.3286786	1.6080016	0.0000000
H	0.6226121	1.2675893	0.0000000
H	-1.0648042	0.9122941	0.0000000
H	3.1222245	-2.4895351	0.0000000
Cl	2.0823969	3.4026242	0.0000000
H	-3.2573973	-1.9629307	0.0000000

69 (BrC···G)

C	0.4690710	-1.8634494	0.0000000
C	0.1690347	-3.2200172	0.0000000
C	2.2655093	-3.9112225	0.0000000
C	1.8414683	-1.4551175	0.0000000
C	-1.6549543	-1.9817325	0.0000000
N	-0.6717111	-1.1025620	0.0000000
N	-1.1965766	-3.2730411	0.0000000
H	-1.7515102	-4.1165614	0.0000000
N	2.6742907	-2.6034074	0.0000000
N	1.0111368	-4.2819309	0.0000000
C	-3.0915531	3.5874619	0.0000000
C	-1.2507363	5.1829135	0.0000000
C	-0.3557183	4.1655971	0.0000000
C	-0.8759660	2.8184342	0.0000000
H	-3.2622596	5.6420139	0.0000000
N	-2.5733001	4.9027171	0.0000000
O	-4.3052211	3.4431498	0.0000000
N	-2.1770655	2.5750606	0.0000000
N	3.2394160	-4.8567391	0.0000000
H	4.2160939	-4.6303148	0.0000000
H	2.9504955	-5.8190094	0.0000000
O	2.3340815	-0.3381876	0.0000000
H	-0.9568942	6.2252141	0.0000000
N	-0.0392769	1.7802020	0.0000000
H	0.9604305	1.8993168	0.0000000
H	-0.4193978	0.8324087	0.0000000
H	3.6638231	-2.3840699	0.0000000
Br	1.4933880	4.5196069	0.0000000
H	-2.7060983	-1.7367337	0.0000000

70 (IC···G)

C	1.2924992	-1.4543103	0.0000000
C	0.6289054	-2.6761727	0.0000000
C	2.4503037	-3.9239934	0.0000000
C	2.7249874	-1.4435666	0.0000000
C	-0.7901340	-0.9860723	0.0000000

N	0.4015027	-0.4113233	0.0000000
N	-0.6975283	-2.3510649	0.0000000
H	-1.4653781	-3.0064934	0.0000000
N	3.2051203	-2.7818256	0.0000000
N	1.1433247	-3.9315257	0.0000000
C	-3.3226990	2.1945806	0.0000000
C	-2.7607294	4.5573763	0.0000000
C	-1.4354204	4.2629435	0.0000000
C	-1.0703461	2.8650360	0.0000000
H	-4.6641296	3.7600059	0.0000000
N	-3.6731909	3.5595469	0.0000000
O	-4.2131630	1.3548171	0.0000000
N	-1.9907449	1.9115265	0.0000000
I	-0.0369673	5.7814749	0.0000000
N	3.1246692	-5.1040211	0.0000000
H	4.1256587	-5.1561644	0.0000000
H	2.5802557	-5.9481790	0.0000000
O	3.5122071	-0.5126833	0.0000000
H	-3.1390064	5.5722815	0.0000000
N	0.2100422	2.4917535	0.0000000
H	0.9433086	3.1805461	0.0000000
H	0.4263249	1.4846825	0.0000000
H	4.2165122	-2.8446158	0.0000000
H	-1.7261847	-0.4445595	0.0000000

71 (C···FG)

C	0.1039596	-2.1341637	0.0000000
C	0.3610892	-3.4988755	0.0000000
C	2.5594245	-3.3218074	0.0000000
C	1.2093898	-1.2330024	0.0000000
C	-1.7727737	-3.0567631	0.0000000
N	-1.2546232	-1.8680433	0.0000000
N	-0.8802641	-4.0881414	0.0000000
H	-1.0881378	-5.0763506	0.0000000
N	2.4224247	-1.9602143	0.0000000
N	1.5448600	-4.1495429	0.0000000
C	-2.6958470	4.6642249	0.0000000
C	-0.2585247	4.7191621	0.0000000
C	-0.2060386	3.3666367	0.0000000
C	-1.4711084	2.6833597	0.0000000
H	-1.5259967	6.3538863	0.0000000
N	-1.4582557	5.3464023	0.0000000
O	-3.7224647	5.3277107	0.0000000
N	-2.6399089	3.2984776	0.0000000
N	3.8225082	-3.8143951	0.0000000
H	4.6346437	-3.2261454	0.0000000
H	3.9311671	-4.8134120	0.0000000
O	1.2328870	-0.0078970	0.0000000
H	0.6228072	5.3491351	0.0000000
N	-1.4598725	1.3410793	0.0000000
H	-0.5926536	0.8214561	0.0000000
H	-2.3370291	0.8470078	0.0000000
H	3.2458860	-1.3688252	0.0000000
H	0.7308060	2.8283887	0.0000000
F	-3.0583543	-3.3293481	0.0000000

72 (C···ClG)

C	0.5152885	-1.8726664	0.0000000
C	0.2180713	-3.2297088	0.0000000
C	2.3142814	-3.9166118	0.0000000
C	1.8854212	-1.4606843	0.0000000
C	-1.5978752	-1.9908086	0.0000000
N	-0.6261286	-1.1080867	0.0000000
N	-1.1504550	-3.2856930	0.0000000
H	-1.7223975	-4.1184478	0.0000000
N	2.7193395	-2.6085811	0.0000000
N	1.0593481	-4.2895254	0.0000000
C	-3.0420317	3.5930009	0.0000000
C	-1.1995128	5.1874196	0.0000000

C	-0.2983469	4.1767762	0.0000000
C	-0.8348609	2.8429807	0.0000000
H	-3.2161895	5.6428013	0.0000000
N	-2.5229548	4.9085257	0.0000000
O	-4.2568280	3.4517029	0.0000000
N	-2.1306890	2.5773076	0.0000000
N	3.2890897	-4.8602922	0.0000000
H	4.2651210	-4.6310146	0.0000000
H	3.0031110	-5.8234927	0.0000000
O	2.3772910	-0.3432258	0.0000000
H	-0.9190123	6.2336854	0.0000000
N	0.0088282	1.8012500	0.0000000
H	1.0071109	1.9183163	0.0000000
H	-0.3670775	0.8557152	0.0000000
H	3.7083355	-2.3865166	0.0000000
H	0.7645635	4.3701166	0.0000000
Cl	-3.2508410	-1.6342422	0.0000000

73 (C···BrG)

C	0.5223383	-1.8740686	0.0000000
C	0.2247356	-3.2313673	0.0000000
C	2.3217673	-3.9176369	0.0000000
C	1.8930568	-1.4622238	0.0000000
C	-1.5929700	-1.9927228	0.0000000
N	-0.6188472	-1.1098640	0.0000000
N	-1.1434919	-3.2873174	0.0000000
H	-1.7146295	-4.1210158	0.0000000
N	2.7273612	-2.6095827	0.0000000
N	1.0669652	-4.2904839	0.0000000
C	-3.0382770	3.5911728	0.0000000
C	-1.1961541	5.1859862	0.0000000
C	-0.2949139	4.1756128	0.0000000
C	-0.8311317	2.8417158	0.0000000
H	-3.2130015	5.6409965	0.0000000
N	-2.5196801	4.9067838	0.0000000
O	-4.2530148	3.4493809	0.0000000
N	-2.1270109	2.5756078	0.0000000
N	3.2960633	-4.8615306	0.0000000
H	4.2724310	-4.6333698	0.0000000
H	3.0086274	-5.8243339	0.0000000
O	2.3843035	-0.3444816	0.0000000
H	-0.9164974	6.2326594	0.0000000
N	0.0121525	1.7997575	0.0000000
H	1.0105136	1.9164841	0.0000000
H	-0.3637280	0.8541486	0.0000000
H	3.7164689	-2.3878341	0.0000000
Br	-3.4014444	-1.5916306	0.0000000
H	0.7680078	4.3691575	0.0000000

74 (C···IG)

C	0.9623352	-1.6460956	0.0000000
C	0.6552183	-3.0013330	0.0000000
C	2.7493709	-3.6996150	0.0000000
C	2.3349521	-1.2415788	0.0000000
C	-1.1625772	-1.7484790	0.0000000
N	-0.1713012	-0.8744868	0.0000000
N	-0.7112671	-3.0455986	0.0000000
H	-1.2885120	-3.8751551	0.0000000
N	3.1636731	-2.3939721	0.0000000
N	1.4932248	-4.0655817	0.0000000
C	-3.4202352	2.7107063	0.0000000
C	-2.2546791	4.8459652	0.0000000
C	-1.0552336	4.2162353	0.0000000
C	-1.0887027	2.7796531	0.0000000
H	-4.3039473	4.5694687	0.0000000
N	-3.3983926	4.1223662	0.0000000
O	-4.5067807	2.1434699	0.0000000
N	-2.2124744	2.0819476	0.0000000
I	-3.1493506	-1.2893291	0.0000000

N	3.7194679	-4.6496385	0.0000000
H	4.6968535	-4.4266415	0.0000000
H	3.4265226	-5.6106471	0.0000000
O	2.8293862	-0.1256127	0.0000000
H	-2.3583224	5.9242948	0.0000000
N	0.0597257	2.0882119	0.0000000
H	0.9517159	2.5502845	0.0000000
H	0.0143049	1.0673685	0.0000000
H	4.1540556	-2.1786296	0.0000000
H	-0.1290305	4.7724221	0.0000000

75 (FC···FG)

C	-0.0582564	-2.0389713	0.0000000
C	-0.3254936	-3.4018805	0.0000000
C	1.7797708	-4.0566541	0.0000000
C	1.3052055	-1.6105595	0.0000000
C	-2.1482369	-2.2058498	0.0000000
N	-1.2258050	-1.2935840	0.0000000
N	-1.6972508	-3.4914471	0.0000000
H	-2.2544106	-4.3336821	0.0000000
N	2.1582807	-2.7429026	0.0000000
N	0.5299092	-4.4474138	0.0000000
C	-2.2615745	4.6092444	0.0000000
C	0.1080857	5.1972049	0.0000000
C	0.4208248	3.8868912	0.0000000
C	-0.6401021	2.9206588	0.0000000
H	-1.4880886	6.5135775	0.0000000
N	-1.2070079	5.5438407	0.0000000
O	-3.4110074	5.0241230	0.0000000
N	-1.9070511	3.2848360	0.0000000
N	2.7684121	-4.9845471	0.0000000
H	3.7412043	-4.7408131	0.0000000
H	2.4969469	-5.9520454	0.0000000
O	1.7913543	-0.4886552	0.0000000
H	0.8542799	5.9807854	0.0000000
N	-0.3184289	1.6234191	0.0000000
H	0.6368037	1.2962916	0.0000000
H	-1.0496484	0.9243638	0.0000000
H	3.1423920	-2.4994017	0.0000000
F	1.7008260	3.4661867	0.0000000
F	-3.4419340	-1.9830159	0.0000000

76 (ClC···ClG)

C	-0.0567127	-2.0369188	0.0000000
C	-0.3255046	-3.4015005	0.0000000
C	1.7811596	-4.0552260	0.0000000
C	1.3095954	-1.6090019	0.0000000
C	-2.1642518	-2.2035085	0.0000000
N	-1.2170285	-1.2947528	0.0000000
N	-1.6916204	-3.4881582	0.0000000
H	-2.2439016	-4.3342876	0.0000000
N	2.1618249	-2.7408394	0.0000000
N	0.5319872	-4.4467856	0.0000000
C	-2.2611276	4.5997013	0.0000000
C	0.1020337	5.1967992	0.0000000
C	0.4430211	3.8863670	0.0000000
C	-0.6288061	2.9197711	0.0000000
H	-1.4899570	6.5099414	0.0000000
N	-1.2065122	5.5401427	0.0000000
O	-3.4097591	5.0155857	0.0000000
N	-1.8990012	3.2823617	0.0000000
N	2.7701778	-4.9824418	0.0000000
H	3.7428465	-4.7384885	0.0000000
H	2.4990803	-5.9500972	0.0000000
O	1.7944365	-0.4870100	0.0000000
H	0.8364550	5.9924800	0.0000000
N	-0.3306559	1.6186797	0.0000000
H	0.6175322	1.2703163	0.0000000
H	-1.0762640	0.9346256	0.0000000

H	3.1464637	-2.4992427	0.0000000
Cl	-3.8300872	-1.8937509	0.0000000
Cl	2.0945761	3.3952388	0.0000000

77 (BrC···BrG)

C	0.4944980	-1.8786845	0.0000000
C	0.1970233	-3.2357253	0.0000000
C	2.2942446	-3.9218965	0.0000000
C	1.8653259	-1.4663374	0.0000000
C	-1.6211469	-1.9976050	0.0000000
N	-0.6469260	-1.1142491	0.0000000
N	-1.1714641	-3.2917545	0.0000000
H	-1.7422776	-4.1256964	0.0000000
N	2.6999186	-2.6137932	0.0000000
N	1.0391682	-4.2947346	0.0000000
C	-3.0652955	3.5869475	0.0000000
C	-1.2271721	5.1857892	0.0000000
C	-0.3300981	4.1701596	0.0000000
C	-0.8486476	2.8228719	0.0000000
H	-3.2390338	5.6415905	0.0000000
N	-2.5490856	4.9031892	0.0000000
O	-4.2783331	3.4397480	0.0000000
N	-2.1483446	2.5764949	0.0000000
N	3.2681424	-4.8660028	0.0000000
H	4.2446371	-4.6382726	0.0000000
H	2.9806730	-5.8288579	0.0000000
O	2.3544642	-0.3481423	0.0000000
H	-0.9350276	6.2284883	0.0000000
N	-0.0101283	1.7849737	0.0000000
H	0.9895012	1.9035997	0.0000000
H	-0.3922585	0.8400238	0.0000000
H	3.6891182	-2.3920934	0.0000000
Br	1.5183940	4.5270254	0.0000000
Br	-3.4298689	-1.5970561	0.0000000

78 (IC···IG)

C	0.9329296	-1.6629553	0.0000000
C	0.6259125	-3.0179940	0.0000000
C	2.7201339	-3.7161862	0.0000000
C	2.3057207	-1.2582576	0.0000000
C	-1.1923589	-1.7659721	0.0000000
N	-0.2011460	-0.8914617	0.0000000
N	-0.7408192	-3.0625448	0.0000000
H	-1.3173556	-3.8925797	0.0000000
N	3.1345676	-2.4105995	0.0000000
N	1.4637070	-4.0821308	0.0000000
C	-3.4463096	2.6921822	0.0000000
C	-2.2884389	4.8303277	0.0000000
C	-1.0856628	4.2000611	0.0000000
C	-1.1006137	2.7558982	0.0000000
H	-4.3333512	4.5546762	0.0000000
N	-3.4283858	4.1038600	0.0000000
O	-4.5291841	2.1203355	0.0000000
N	-2.2348025	2.0731389	0.0000000
I	0.6622423	5.2989592	0.0000000
I	-3.1788951	-1.3059105	0.0000000
N	3.6895758	-4.6663802	0.0000000
H	4.6672067	-4.4441841	0.0000000
H	3.3955796	-5.6270978	0.0000000
O	2.7987187	-0.1419723	0.0000000
H	-2.3888674	5.9088139	0.0000000
N	0.0365259	2.0576178	0.0000000
H	0.9350189	2.5101336	0.0000000
H	-0.0266859	1.0355122	0.0000000
H	4.1250374	-2.1952901	0.0000000

Binding mode c

79 (C···G)

C	2.0751401	0.3289242	0.0000000
C	3.4404046	0.5989806	0.0000000
C	3.2430538	2.7975103	0.0000000
C	1.1640946	1.4319279	0.0000000
C	3.0340119	-1.5639685	0.0000000
N	1.8311009	-1.0232208	0.0000000
N	4.0367510	-0.6287199	0.0000000
H	5.0322313	-0.7977436	0.0000000
N	1.8809535	2.6497881	0.0000000
N	4.0799445	1.7930947	0.0000000
C	-4.2618867	-3.1944630	0.0000000
C	-4.8338687	-0.8248509	0.0000000
C	-3.5232766	-0.4869698	0.0000000
C	-2.5850574	-1.5769053	0.0000000
H	-6.1622073	-2.4102519	0.0000000
N	-5.1921170	-2.1301846	0.0000000
O	-4.6930667	-4.3386092	0.0000000
N	-2.9398235	-2.8502700	0.0000000
N	3.7210978	4.0671340	0.0000000
H	3.1237196	4.8723820	0.0000000
H	4.7187412	4.1868362	0.0000000
O	-0.0607434	1.4520632	0.0000000
H	-5.6362648	-0.0970134	0.0000000
N	-1.2771022	-1.2832903	0.0000000
H	-0.9373432	-0.3301998	0.0000000
H	-0.6022631	-2.0309348	0.0000000
H	1.2834270	3.4685873	0.0000000
H	3.2399632	-2.6234923	0.0000000
H	-3.1996145	0.5438595	0.0000000

80 (FC···G)

C	3.2471344	-0.3721541	0.0000000
C	3.8412809	0.8879587	0.0000000
C	1.9577877	2.0353455	0.0000000
C	1.8193166	-0.4450886	0.0000000
C	5.3368385	-0.7257398	0.0000000
N	4.1885123	-1.3720969	0.0000000
N	5.1830598	0.6383357	0.0000000
H	5.9096899	1.3389664	0.0000000
N	1.2673642	0.8541098	0.0000000
N	3.2630700	2.1135738	0.0000000
C	-5.2045008	-1.7693060	0.0000000
C	-4.1043972	0.4066177	0.0000000
C	-2.9086476	-0.2136008	0.0000000
C	-2.8597768	-1.6470261	0.0000000
H	-6.1427607	0.0651812	0.0000000
N	-5.2250020	-0.3569677	0.0000000
O	-6.2659153	-2.3689672	0.0000000
N	-3.9670087	-2.3608315	0.0000000
N	1.2157144	3.1720678	0.0000000
H	0.2127971	3.1601337	0.0000000
H	1.7064668	4.0486327	0.0000000
O	1.0778862	-1.4210543	0.0000000
H	-4.2071662	1.4839032	0.0000000
N	-1.6750020	-2.2685701	0.0000000
H	-0.7760150	-1.7934764	0.0000000
H	-1.6976259	-3.2766234	0.0000000
H	0.2535320	0.8658477	0.0000000
F	-1.7583066	0.5100104	0.0000000
H	6.3116740	-1.1891812	0.0000000

81 (ClC···G)

C	3.2794024	-0.3341800	0.0000000
C	4.0364275	0.8351302	0.0000000
C	2.3241819	2.2266853	0.0000000
C	1.8541506	-0.2164162	0.0000000

C	5.3032627	-0.9635848	0.0000000
N	4.0787725	-1.4507589	0.0000000
N	5.3327913	0.4087118	0.0000000
H	6.1464302	1.0063051	0.0000000
N	1.4822536	1.1475957	0.0000000
N	3.6277298	2.1273650	0.0000000
C	-5.2870452	-2.1834429	0.0000000
C	-4.6482665	0.1661356	0.0000000
C	-3.3385701	-0.1814317	0.0000000
C	-3.0166383	-1.5868076	0.0000000
H	-6.5735805	-0.5697967	0.0000000
N	-5.5894046	-0.8006743	0.0000000
O	-6.2099209	-2.9797809	0.0000000
N	-3.9596019	-2.5096095	0.0000000
N	1.7442855	3.4541140	0.0000000
H	0.7498104	3.5831981	0.0000000
H	2.3515019	4.2545470	0.0000000
O	0.9873376	-1.0808701	0.0000000
H	-4.9782285	1.1974524	0.0000000
N	-1.7404863	-1.9853331	0.0000000
H	-0.9350535	-1.3701888	0.0000000
H	-1.5821859	-2.9813527	0.0000000
H	0.4784587	1.2892982	0.0000000
Cl	-2.1255978	1.0503590	0.0000000
H	6.2077836	-1.5526692	0.0000000

82 (BrC···G)

C	3.2785078	-0.3284448	0.0000000
C	4.0775326	0.8125323	0.0000000
C	2.4169388	2.2654723	0.0000000
C	1.8584327	-0.1588482	0.0000000
C	5.2781739	-1.0309986	0.0000000
N	4.0367696	-1.4733413	0.0000000
N	5.3575495	0.3393015	0.0000000
H	6.1923645	0.9069402	0.0000000
N	1.5362406	1.2178821	0.0000000
N	3.7160085	2.1187727	0.0000000
C	-5.2852842	-2.2858025	0.0000000
C	-4.7836597	0.0960595	0.0000000
C	-3.4544150	-0.1735085	0.0000000
C	-3.0514629	-1.5584212	0.0000000
H	-6.6633971	-0.7484193	0.0000000
N	-5.6674023	-0.9225327	0.0000000
O	-6.1604643	-3.1341255	0.0000000
N	-3.9416679	-2.5333689	0.0000000
N	1.8828210	3.5136314	0.0000000
H	0.8939604	3.6796784	0.0000000
H	2.5192967	4.2910208	0.0000000
O	0.9604393	-0.9906975	0.0000000
H	-5.1777192	1.1047329	0.0000000
N	-1.7570626	-1.8918999	0.0000000
H	-0.9810032	-1.2411547	0.0000000
H	-1.5483754	-2.8787692	0.0000000
H	0.5374827	1.3945296	0.0000000
Br	-2.2313152	1.2623203	0.0000000
H	6.1607104	-1.6525412	0.0000000

83 (IC···G)

C	3.2711186	-0.3209769	0.0000000
C	4.1131545	0.7884451	0.0000000
C	2.5083013	2.3034871	0.0000000
C	1.8584022	-0.0969999	0.0000000
C	5.2422873	-1.0998273	0.0000000
N	3.9847575	-1.4941648	0.0000000
N	5.3743928	0.2665137	0.0000000
H	6.2310698	0.8006935	0.0000000
N	1.5883294	1.2906329	0.0000000
N	3.8009001	2.1073429	0.0000000
C	-5.2719625	-2.3884188	0.0000000

C	-4.9102188	0.0177931	0.0000000
C	-3.5643286	-0.1676161	0.0000000
C	-3.0848638	-1.5293799	0.0000000
H	-6.7375567	-0.9336308	0.0000000
N	-5.7329331	-1.0496409	0.0000000
O	-6.0962438	-3.2863285	0.0000000
N	-3.9169358	-2.5554413	0.0000000
I	-2.3444649	1.5010988	0.0000000
N	2.0225513	3.5715584	0.0000000
H	1.0409800	3.7759355	0.0000000
H	2.6885700	4.3236863	0.0000000
O	0.9294093	-0.8938701	0.0000000
H	-5.3702799	0.9984220	0.0000000
N	-1.7749783	-1.7964082	0.0000000
H	-1.0305378	-1.1108788	0.0000000
H	-1.5150852	-2.7712209	0.0000000
H	0.5961801	1.5042772	0.0000000
H	6.0999852	-1.7550837	0.0000000

84 (C···FG)

C	2.1157392	0.2994080	0.0000000
C	3.4510668	0.6807849	0.0000000
C	3.0728913	2.8535807	0.0000000
C	1.1168941	1.3173903	0.0000000
C	3.2067451	-1.4846714	0.0000000
N	1.9754785	-1.0778541	0.0000000
N	4.1518167	-0.5012221	0.0000000
H	5.1549441	-0.6173719	0.0000000
N	1.7296367	2.5920667	0.0000000
N	3.9902899	1.9193106	0.0000000
C	-4.3970383	-3.1126729	0.0000000
C	-4.6756422	-0.6907382	0.0000000
C	-3.3336662	-0.5141991	0.0000000
C	-2.5370493	-1.7111413	0.0000000
H	-6.1870124	-2.1030296	0.0000000
N	-5.1900247	-1.9430036	0.0000000
O	-4.9633759	-4.1959041	0.0000000
N	-3.0422206	-2.9314840	0.0000000
N	3.4473273	4.1565962	0.0000000
H	2.7868508	4.9111869	0.0000000
H	4.4321130	4.3567386	0.0000000
O	-0.1052049	1.2283284	0.0000000
H	-5.3839400	0.1289611	0.0000000
N	-1.2014606	-1.5767122	0.0000000
H	-0.7635612	-0.6654761	0.0000000
H	-0.6289723	-2.4048322	0.0000000
H	1.0650137	3.3576472	0.0000000
F	3.5961580	-2.7398178	0.0000000
H	-2.8837966	0.4681312	0.0000000

85 (C···ClG)

C	2.0971487	0.3275054	0.0000000
C	3.4418956	0.6816946	0.0000000
C	3.1058384	2.8622649	0.0000000
C	1.1177070	1.3680888	0.0000000
C	3.1605577	-1.4936304	0.0000000
N	1.9297862	-1.0391059	0.0000000
N	4.1144260	-0.5100861	0.0000000
H	5.1164649	-0.6401797	0.0000000
N	1.7562808	2.6291086	0.0000000
N	4.0048085	1.9108270	0.0000000
C	-4.3644940	-3.1268778	0.0000000
C	-4.7126840	-0.7139694	0.0000000
C	-3.3763197	-0.4991863	0.0000000
C	-2.5452876	-1.6725730	0.0000000
H	-6.1829020	-2.1689398	0.0000000
N	-5.1908954	-1.9804344	0.0000000
O	-4.8996587	-4.2259056	0.0000000
N	-3.0155478	-2.9069793	0.0000000

N	3.5053281	4.1575327	0.0000000
H	2.8598908	4.9250400	0.0000000
H	4.4939381	4.3381095	0.0000000
O	-0.1052685	1.3036147	0.0000000
H	-5.4441214	0.0851817	0.0000000
N	-1.2144771	-1.4999801	0.0000000
H	-0.8003580	-0.5776444	0.0000000
H	-0.6163331	-2.3098498	0.0000000
H	1.1084699	3.4089615	0.0000000
Cl	3.6114040	-3.1286041	0.0000000
H	-2.9555971	0.4960166	0.0000000

86 (C···BrG)

C	2.0916098	0.3393047	0.0000000
C	3.4387732	0.6856301	0.0000000
C	3.1149282	2.8682120	0.0000000
C	1.1178368	1.3859774	0.0000000
C	3.1456104	-1.4894470	0.0000000
N	1.9164727	-1.0258568	0.0000000
N	4.1041324	-0.5099252	0.0000000
H	5.1057714	-0.6441800	0.0000000
N	1.7637850	2.6430860	0.0000000
N	4.0085226	1.9118074	0.0000000
C	-4.3524967	-3.1328056	0.0000000
C	-4.7249632	-0.7235317	0.0000000
C	-3.3908075	-0.4954027	0.0000000
C	-2.5479275	-1.6603141	0.0000000
H	-6.1804776	-2.1931681	0.0000000
N	-5.1904165	-1.9947086	0.0000000
O	-4.8766666	-4.2371363	0.0000000
N	-3.0058438	-2.8994016	0.0000000
N	3.5216601	4.1611555	0.0000000
H	2.8806070	4.9323268	0.0000000
H	4.5113103	4.3360731	0.0000000
O	-0.1053540	1.3285083	0.0000000
H	-5.4643309	0.0682475	0.0000000
N	-1.2189691	-1.4745273	0.0000000
H	-0.8130753	-0.5485609	0.0000000
H	-0.6123759	-2.2781365	0.0000000
H	1.1207202	3.4268550	0.0000000
Br	3.6223089	-3.2841648	0.0000000
H	-2.9803445	0.5040834	0.0000000

87 (C···IG)

C	2.0761815	0.3658267	0.0000000
C	3.4312565	0.6821844	0.0000000
C	3.1558236	2.8717632	0.0000000
C	1.1258407	1.4348322	0.0000000
C	3.0922292	-1.4907991	0.0000000
N	1.8718030	-0.9938146	0.0000000
N	4.0694198	-0.5269116	0.0000000
H	5.0686659	-0.6787652	0.0000000
N	1.7998166	2.6770054	0.0000000
N	4.0278824	1.8959526	0.0000000
C	-4.3037396	-3.1631784	0.0000000
C	-4.7696860	-0.7702865	0.0000000
C	-3.4453566	-0.4907233	0.0000000
C	-2.5574262	-1.6217246	0.0000000
H	-6.1670282	-2.2952853	0.0000000
N	-5.1854339	-2.0585020	0.0000000
O	-4.7848145	-4.2870160	0.0000000
N	-2.9673320	-2.8777642	0.0000000
I	3.5558808	-3.4803166	0.0000000
N	3.5909354	4.1555733	0.0000000
H	2.9670147	4.9406490	0.0000000
H	4.5841976	4.3085708	0.0000000
O	-0.0983338	1.4060450	0.0000000
H	-5.5391095	-0.0076832	0.0000000
N	-1.2369527	-1.3852794	0.0000000

H	-0.8628580	-0.4457539	0.0000000
H	-0.5983837	-2.1638949	0.0000000
H	1.1744891	3.4749878	0.0000000
H	-3.0749818	0.5243083	0.0000000

88 (FC···FG)

C	3.2391919	-0.3678931	0.0000000
C	3.8303254	0.8909055	0.0000000
C	1.9487729	2.0372323	0.0000000
C	1.8148296	-0.4440158	0.0000000
C	5.2999056	-0.7166409	0.0000000
N	4.1861558	-1.3776265	0.0000000
N	5.1816415	0.6427265	0.0000000
H	5.9272620	1.3232366	0.0000000
N	1.2617801	0.8563588	0.0000000
N	3.2561226	2.1139146	0.0000000
C	-5.2122854	-1.7656537	0.0000000
C	-4.1119049	0.4101569	0.0000000
C	-2.9161864	-0.2103248	0.0000000
C	-2.8676144	-1.6436580	0.0000000
H	-6.1502798	0.0692174	0.0000000
N	-5.2325403	-0.3531572	0.0000000
O	-6.2735064	-2.3653745	0.0000000
N	-3.9747114	-2.3572347	0.0000000
N	1.2098779	3.1748525	0.0000000
H	0.2068632	3.1638729	0.0000000
H	1.7015691	4.0509550	0.0000000
O	1.0746824	-1.4210963	0.0000000
H	-4.2146395	1.4874234	0.0000000
N	-1.6829021	-2.2657301	0.0000000
H	-0.7842278	-1.7910397	0.0000000
H	-1.7055009	-3.2737651	0.0000000
H	0.2476087	0.8652414	0.0000000
F	6.5049853	-1.2462382	0.0000000
F	-1.7652748	0.5133550	0.0000000

89 (ClC···ClG)

C	3.2614200	-0.3214341	0.0000000
C	4.0168716	0.8480759	0.0000000
C	2.3045201	2.2373080	0.0000000
C	1.8365946	-0.2064990	0.0000000
C	5.2729984	-0.9480457	0.0000000
N	4.0597111	-1.4430895	0.0000000
N	5.3170331	0.4217695	0.0000000
H	6.1440650	1.0018288	0.0000000
N	1.4643849	1.1580170	0.0000000
N	3.6095756	2.1377108	0.0000000
C	-5.3058808	-2.1718729	0.0000000
C	-4.6665552	0.1777193	0.0000000
C	-3.3569353	-0.1702818	0.0000000
C	-3.0354772	-1.5756781	0.0000000
H	-6.5919479	-0.5576563	0.0000000
N	-5.6078057	-0.7888412	0.0000000
O	-6.2286446	-2.9679981	0.0000000
N	-3.9783204	-2.4982034	0.0000000
N	1.7268993	3.4643986	0.0000000
H	0.7324486	3.5947742	0.0000000
H	2.3349355	4.2643893	0.0000000
O	0.9720612	-1.0724665	0.0000000
H	-4.9964927	1.2090577	0.0000000
N	-1.7592713	-1.9745959	0.0000000
H	-0.9548167	-1.3589889	0.0000000
H	-1.6006587	-2.9705262	0.0000000
H	0.4601678	1.2987808	0.0000000
Cl	-2.1434144	1.0615061	0.0000000
Cl	6.7125342	-1.8491584	0.0000000

90 (BrC···BrG)

C	3.2566336	-0.3118200	0.0000000
C	4.0543241	0.8296216	0.0000000
C	2.3931299	2.2800681	0.0000000
C	1.8363521	-0.1449230	0.0000000
C	5.2457517	-1.0114650	0.0000000
N	4.0133443	-1.4614773	0.0000000
N	5.3379198	0.3563062	0.0000000
H	6.1847380	0.9073710	0.0000000
N	1.5139223	1.2321499	0.0000000
N	3.6936679	2.1332409	0.0000000
C	-5.3082978	-2.2704359	0.0000000
C	-4.8061200	0.1114702	0.0000000
C	-3.4769231	-0.1585207	0.0000000
C	-3.0745089	-1.5434875	0.0000000
H	-6.6858935	-0.7324253	0.0000000
N	-5.6899377	-0.9068956	0.0000000
O	-6.1833822	-3.1185340	0.0000000
N	-3.9645533	-2.5181560	0.0000000
N	1.8611789	3.5276546	0.0000000
H	0.8724037	3.6951251	0.0000000
H	2.4984531	4.3045783	0.0000000
O	0.9410123	-0.9785917	0.0000000
H	-5.2002237	1.1201247	0.0000000
N	-1.7799994	-1.8773219	0.0000000
H	-1.0051388	-1.2259075	0.0000000
H	-1.5709982	-2.8641134	0.0000000
H	0.5147005	1.4080139	0.0000000
Br	6.7817772	-2.0589401	0.0000000
Br	-2.2533328	1.2772902	0.0000000

91 (IC···IG)

C	3.2446531	-0.2997347	0.0000000
C	4.0857426	0.8106369	0.0000000
C	2.4804605	2.3232128	0.0000000
C	1.8312526	-0.0780733	0.0000000
C	5.2101564	-1.0771469	0.0000000
N	3.9560415	-1.4762960	0.0000000
N	5.3492851	0.2887989	0.0000000
H	6.2145256	0.8107616	0.0000000
N	1.5614735	1.3099802	0.0000000
N	3.7743716	2.1273137	0.0000000
C	-5.2990935	-2.3683333	0.0000000
C	-4.9370365	0.0380139	0.0000000
C	-3.5911221	-0.1475908	0.0000000
C	-3.1121309	-1.5094202	0.0000000
H	-6.7643603	-0.9130431	0.0000000
N	-5.7597274	-1.0292969	0.0000000
O	-6.1231340	-3.2661226	0.0000000
N	-3.9439222	-2.5353131	0.0000000
I	-2.3709352	1.5211904	0.0000000
I	6.8457313	-2.3044103	0.0000000
N	1.9965461	3.5904768	0.0000000
H	1.0149864	3.7953791	0.0000000
H	2.6630565	4.3424744	0.0000000
O	0.9046629	-0.8767678	0.0000000
H	-5.3972071	1.0185802	0.0000000
N	-1.8021469	-1.7766798	0.0000000
H	-1.0589985	-1.0903827	0.0000000
H	-1.5420623	-2.7514359	0.0000000
H	0.5689315	1.5232286	0.0000000

Binding mode d

92 (C···G)

C	0.0233420	-1.8063236	0.0000000
C	-0.2538011	-3.1701993	0.0000000
C	1.8500163	-3.8370705	0.0000000
C	1.3920567	-1.3911196	0.0000000
C	-2.0926230	-1.9614178	0.0000000

N	-1.1294767	-1.0599495	0.0000000
N	-1.6167326	-3.2471640	0.0000000
H	-2.1560769	-4.1007659	0.0000000
N	2.2388483	-2.5237329	0.0000000
N	0.6001202	-4.2214906	0.0000000
C	-1.0408022	5.3059610	0.0000000
C	-2.2436476	3.1867333	0.0000000
C	-1.0803838	2.4936647	0.0000000
C	0.1174787	3.2853847	0.0000000
H	-3.0813369	5.0792157	0.0000000
N	-2.2267677	4.5413350	0.0000000
O	-1.1341319	6.5251662	0.0000000
N	0.1338476	4.6059525	0.0000000
N	2.8363902	-4.7680239	0.0000000
H	3.8098281	-4.5274750	0.0000000
H	2.5619671	-5.7346467	0.0000000
O	1.8852378	-0.2715777	0.0000000
H	-3.2172096	2.7112733	0.0000000
N	1.3031485	2.6484028	0.0000000
H	1.3746502	1.6396657	0.0000000
H	2.1327380	3.2186670	0.0000000
H	3.2247663	-2.2880582	0.0000000
H	-3.1498774	-1.7442319	0.0000000
H	-1.0615687	1.4118253	0.0000000

93 (FC···G)

C	-0.0742271	-2.1871827	0.0000000
C	-0.1340883	-3.5781846	0.0000000
C	2.0480715	-3.9079292	0.0000000
C	1.2122989	-1.5633027	0.0000000
C	-2.1375740	-2.6686590	0.0000000
N	-1.3281675	-1.6285428	0.0000000
N	-1.4679519	-3.8670403	0.0000000
H	-1.8674435	-4.7941541	0.0000000
N	2.2266400	-2.5500455	0.0000000
N	0.8744975	-4.4835524	0.0000000
C	-0.6377923	5.6738717	0.0000000
C	-2.3916510	3.9762265	0.0000000
C	-1.4488246	3.0127204	0.0000000
C	-0.0693621	3.4028308	0.0000000
H	-2.6611112	6.0277972	0.0000000
N	-1.9876077	5.2752742	0.0000000
O	-0.3757784	6.8671928	0.0000000
N	0.2960561	4.6681787	0.0000000
N	3.1694398	-4.6728567	0.0000000
H	4.0927950	-4.2822183	0.0000000
H	3.0498665	-5.6703904	0.0000000
O	1.5250707	-0.3803119	0.0000000
H	-3.4517767	3.7600131	0.0000000
N	0.8679088	2.4449638	0.0000000
H	0.6617795	1.4541495	0.0000000
H	1.8293941	2.7445232	0.0000000
H	3.1636985	-2.1638255	0.0000000
F	-1.7683473	1.7087537	0.0000000
H	-3.2158133	-2.6182995	0.0000000

94 (ClC···G)

C	-0.1633228	-2.2424061	0.0000000
C	-0.1592948	-3.6354714	0.0000000
C	2.0346520	-3.8702304	0.0000000
C	1.0966129	-1.5622534	0.0000000
C	-2.2041099	-2.8242993	0.0000000
N	-1.4447571	-1.7466743	0.0000000
N	-1.4778502	-3.9876602	0.0000000
H	-1.8313052	-4.9334071	0.0000000
N	2.1523114	-2.5063826	0.0000000
N	0.8869940	-4.4960522	0.0000000
C	-0.5990368	5.7310580	0.0000000
C	-2.3276352	4.0118468	0.0000000

C	-1.3832631	3.0406549	0.0000000
C	-0.0066515	3.4675468	0.0000000
H	-2.6300538	6.0541880	0.0000000
N	-1.9454176	5.3110988	0.0000000
O	-0.3591384	6.9287403	0.0000000
N	0.3435118	4.7392452	0.0000000
N	3.1872300	-4.5865419	0.0000000
H	4.0932884	-4.1573205	0.0000000
H	3.1098646	-5.5883134	0.0000000
O	1.3654791	-0.3692495	0.0000000
H	-3.3885822	3.7954576	0.0000000
N	0.9607890	2.5397539	0.0000000
H	0.7782088	1.5441176	0.0000000
H	1.9105103	2.8748571	0.0000000
H	3.0708546	-2.0777149	0.0000000
Cl	-1.7863690	1.3702064	0.0000000
H	-3.2835191	-2.8247942	0.0000000

95 (BrC···G)

C	-0.1798147	-2.2905301	0.0000000
C	-0.1519378	-3.6835852	0.0000000
C	2.0451202	-3.8829030	0.0000000
C	1.0693231	-1.5884279	0.0000000
C	-2.2114486	-2.9098053	0.0000000
N	-1.4709158	-1.8188519	0.0000000
N	-1.4643001	-4.0588176	0.0000000
H	-1.8005702	-5.0107794	0.0000000
N	2.1397867	-2.5173111	0.0000000
N	0.9077360	-4.5275215	0.0000000
C	-0.5692218	5.7813670	0.0000000
C	-2.3248161	4.0905626	0.0000000
C	-1.3971362	3.1021474	0.0000000
C	-0.0135675	3.5074654	0.0000000
H	-2.5953202	6.1373478	0.0000000
N	-1.9226038	5.3831416	0.0000000
O	-0.3101022	6.9749448	0.0000000
N	0.3563425	4.7743452	0.0000000
N	3.2094502	-4.5798605	0.0000000
H	4.1082216	-4.1355550	0.0000000
H	3.1496704	-5.5828666	0.0000000
O	1.3198045	-0.3918635	0.0000000
H	-3.3902774	3.8966935	0.0000000
N	0.9474917	2.5713325	0.0000000
H	0.7586380	1.5772761	0.0000000
H	1.8992565	2.9008779	0.0000000
H	3.0516007	-2.0745184	0.0000000
Br	-1.8697609	1.2839198	0.0000000
H	-3.2906485	-2.9282244	0.0000000

96 (IC···G)

C	-0.1962330	-2.3480290	0.0000000
C	-0.1418494	-3.7401155	0.0000000
C	2.0591375	-3.8992918	0.0000000
C	1.0414480	-1.6222266	0.0000000
C	-2.2175242	-3.0103968	0.0000000
N	-1.4982230	-1.9048334	0.0000000
N	-1.4467173	-4.1424592	0.0000000
H	-1.7635900	-5.1013317	0.0000000
N	2.1285273	-2.5327375	0.0000000
N	0.9330412	-4.5638403	0.0000000
C	-0.5423411	5.8427991	0.0000000
C	-2.3225196	4.1795258	0.0000000
C	-1.4142268	3.1707337	0.0000000
C	-0.0247332	3.5599404	0.0000000
H	-2.5627451	6.2299976	0.0000000
N	-1.9015007	5.4653977	0.0000000
O	-0.2644128	7.0323420	0.0000000
N	0.3661346	4.8212965	0.0000000
I	-1.9628179	1.1813292	0.0000000

N	3.2356241	-4.5750702	0.0000000
H	4.1266228	-4.1149084	0.0000000
H	3.1940717	-5.5790060	0.0000000
O	1.2709689	-0.4223184	0.0000000
H	-3.3923867	4.0097334	0.0000000
N	0.9329883	2.6173798	0.0000000
H	0.7410122	1.6246348	0.0000000
H	1.8859944	2.9435947	0.0000000
H	3.0321841	-2.0734270	0.0000000
H	-3.2959341	-3.0487128	0.0000000

97 (C···FG)

C	-0.0045781	-1.8152648	0.0000000
C	-0.2649162	-3.1800512	0.0000000
C	1.8435657	-3.8239971	0.0000000
C	1.3552888	-1.3826806	0.0000000
C	-2.0895646	-1.9898299	0.0000000
N	-1.1730283	-1.0728608	0.0000000
N	-1.6361507	-3.2753690	0.0000000
H	-2.1894789	-4.1201599	0.0000000
N	2.2150478	-2.5077888	0.0000000
N	0.5955824	-4.2209364	0.0000000
C	-1.0444053	5.2976287	0.0000000
C	-2.1936866	3.1489140	0.0000000
C	-1.0127025	2.4866563	0.0000000
C	0.1645272	3.3075023	0.0000000
H	-3.0786211	5.0192212	0.0000000
N	-2.2105858	4.5032102	0.0000000
O	-1.1678612	6.5139493	0.0000000
N	0.1481200	4.6275012	0.0000000
N	2.8371688	-4.7459493	0.0000000
H	3.8085517	-4.4965603	0.0000000
H	2.5716361	-5.7151506	0.0000000
O	1.8356747	-0.2574879	0.0000000
H	-3.1537648	2.6469449	0.0000000
N	1.3658135	2.6987517	0.0000000
H	1.4543312	1.6921011	0.0000000
H	2.1825329	3.2870468	0.0000000
H	3.1980383	-2.2594639	0.0000000
F	-3.3868872	-1.7722467	0.0000000
H	-0.9696479	1.4063694	0.0000000

98 (C···ClG)

C	0.0753227	-1.8012556	0.0000000
C	-0.2161421	-3.1612688	0.0000000
C	1.8796748	-3.8485958	0.0000000
C	1.4466908	-1.3974281	0.0000000
C	-2.0301453	-1.9293621	0.0000000
N	-1.0690851	-1.0366588	0.0000000
N	-1.5837702	-3.2239086	0.0000000
H	-2.1510727	-4.0601054	0.0000000
N	2.2815531	-2.5399687	0.0000000
N	0.6241136	-4.2199225	0.0000000
C	-1.0514076	5.2934441	0.0000000
C	-2.2427050	3.1678231	0.0000000
C	-1.0750861	2.4821181	0.0000000
C	0.1182181	3.2796228	0.0000000
H	-3.0908662	5.0552364	0.0000000
N	-2.2331690	4.5222431	0.0000000
O	-1.1508127	6.5120318	0.0000000
N	0.1274441	4.5998909	0.0000000
N	2.8535070	-4.7909293	0.0000000
H	3.8300362	-4.5625710	0.0000000
H	2.5673099	-5.7543078	0.0000000
O	1.9474538	-0.2819047	0.0000000
H	-3.2127328	2.6851324	0.0000000
N	1.3083444	2.6494438	0.0000000
H	1.3873876	1.6417832	0.0000000
H	2.1345588	3.2245462	0.0000000
H	3.2701137	-2.3147091	0.0000000

Cl	-3.6935779	-1.5914064	0.0000000
H	-1.0511558	1.4009866	0.0000000

99 (C···BrG)

C	0.0955424	-1.7965344	0.0000000
C	-0.2034874	-3.1551675	0.0000000
C	1.8891244	-3.8528377	0.0000000
C	1.4694438	-1.3994433	0.0000000
C	-2.0122789	-1.9133013	0.0000000
N	-1.0441241	-1.0260184	0.0000000
N	-1.5713397	-3.2101639	0.0000000
H	-2.1418580	-4.0443670	0.0000000
N	2.2982507	-2.5461619	0.0000000
N	0.6317649	-4.2179875	0.0000000
C	-1.0561499	5.2877334	0.0000000
C	-2.2487987	3.1628786	0.0000000
C	-1.0815975	2.4764510	0.0000000
C	0.1122363	3.2731655	0.0000000
H	-3.0957307	5.0508984	0.0000000
N	-2.2384180	4.5172977	0.0000000
O	-1.1548749	6.5063787	0.0000000
N	0.1222846	4.5934473	0.0000000
N	2.8581208	-4.7999999	0.0000000
H	3.8358587	-4.5767709	0.0000000
H	2.5668603	-5.7618682	0.0000000
O	1.9749507	-0.2862334	0.0000000
H	-3.2191509	2.6807446	0.0000000
N	1.3021810	2.6425036	0.0000000
H	1.3821265	1.6349257	0.0000000
H	2.1285476	3.2174358	0.0000000
H	3.2880590	-2.3263730	0.0000000
Br	-3.8297238	-1.5259326	0.0000000
H	-1.0578193	1.3953010	0.0000000

100 (C···IG)

C	0.1354107	-1.7851446	0.0000000
C	-0.1797054	-3.1403098	0.0000000
C	1.9058155	-3.8604616	0.0000000
C	1.5139716	-1.4028583	0.0000000
C	-1.9787883	-1.8764651	0.0000000
N	-0.9935078	-1.0022907	0.0000000
N	-1.5473059	-3.1788789	0.0000000
H	-2.1241014	-4.0089668	0.0000000
N	2.3302038	-2.5582327	0.0000000
N	0.6446993	-4.2120731	0.0000000
C	-1.0709206	5.2760080	0.0000000
C	-2.2703585	3.1549826	0.0000000
C	-1.1053741	2.4648350	0.0000000
C	0.0911185	3.2576296	0.0000000
H	-3.1113187	5.0457198	0.0000000
N	-2.2557429	4.5093913	0.0000000
O	-1.1658657	6.4949822	0.0000000
N	0.1052638	4.5779760	0.0000000
I	-3.9681573	-1.4070333	0.0000000
N	2.8645260	-4.8181387	0.0000000
H	3.8446185	-4.6055267	0.0000000
H	2.5626786	-5.7767386	0.0000000
O	2.0295734	-0.2943464	0.0000000
H	-3.2422611	2.6757917	0.0000000
N	1.2793707	2.6237226	0.0000000
H	1.3599365	1.6161084	0.0000000
H	2.1070899	3.1968144	0.0000000
H	3.3225173	-2.3501184	0.0000000
H	-1.0833862	1.3836222	0.0000000

101 (FC···FG)

C	-0.0765051	-2.1234752	0.0000000
C	-0.1639424	-3.5112792	0.0000000

C	2.0077979	-3.8896077	0.0000000
C	1.2197721	-1.5269325	0.0000000
C	-2.1208264	-2.5556777	0.0000000
N	-1.3274486	-1.5321787	0.0000000
N	-1.5118134	-3.7768457	0.0000000
H	-1.9557127	-4.6836058	0.0000000
N	2.2126239	-2.5377873	0.0000000
N	0.8193080	-4.4381508	0.0000000
C	-0.7239794	5.6288198	0.0000000
C	-2.3413120	3.8003461	0.0000000
C	-1.3264818	2.9132951	0.0000000
C	0.0180324	3.4093516	0.0000000
H	-2.7680684	5.8246024	0.0000000
N	-2.0384186	5.1265216	0.0000000
O	-0.5555261	6.8388842	0.0000000
N	0.2857206	4.6985313	0.0000000
N	3.1092150	-4.6815219	0.0000000
H	4.0419101	-4.3134003	0.0000000
H	2.9657283	-5.6760189	0.0000000
O	1.5620247	-0.3518071	0.0000000
H	-3.3813459	3.5017806	0.0000000
N	1.0253013	2.5239909	0.0000000
H	0.8845988	1.5211577	0.0000000
H	1.9608416	2.8966882	0.0000000
H	3.1571510	-2.1695834	0.0000000
F	-1.5426371	1.5890243	0.0000000
F	-3.4360077	-2.5051218	0.0000000

102 (ClC···ClG)

C	-0.1281711	-2.2474504	0.0000000
C	-0.1298100	-3.6398194	0.0000000
C	2.0617928	-3.8834405	0.0000000
C	1.1328133	-1.5709001	0.0000000
C	-2.1601490	-2.8140164	0.0000000
N	-1.4085234	-1.7400227	0.0000000
N	-1.4534541	-3.9871632	0.0000000
H	-1.8324674	-4.9236621	0.0000000
N	2.1844227	-2.5208930	0.0000000
N	0.9097341	-4.5043362	0.0000000
C	-0.5810075	5.7399588	0.0000000
C	-2.3159170	4.0271928	0.0000000
C	-1.3749244	3.0525550	0.0000000
C	0.0030896	3.4741611	0.0000000
H	-2.6110180	6.0704214	0.0000000
N	-1.9289684	5.3248494	0.0000000
O	-0.3363443	6.9364982	0.0000000
N	0.3578147	4.7443209	0.0000000
N	3.2093069	-4.6057389	0.0000000
H	4.1178074	-4.1812863	0.0000000
H	3.1272259	-5.6072853	0.0000000
O	1.4035248	-0.3788660	0.0000000
H	-3.3776325	3.8144635	0.0000000
N	0.9681491	2.5432128	0.0000000
H	0.7826070	1.5486976	0.0000000
H	1.9189637	2.8752478	0.0000000
H	3.1051029	-2.0964683	0.0000000
Cl	-3.8576511	-2.8340923	0.0000000
Cl	-1.7863168	1.3838620	0.0000000

103 (BrC···BrG)

C	-0.1511499	-2.2913502	0.0000000
C	-0.1229155	-3.6839954	0.0000000
C	2.0732754	-3.8815932	0.0000000
C	1.0967721	-1.5876140	0.0000000
C	-2.1733242	-2.9053043	0.0000000
N	-1.4431873	-1.8143036	0.0000000
N	-1.4385176	-4.0606744	0.0000000
H	-1.7943622	-5.0064410	0.0000000
N	2.1671548	-2.5168864	0.0000000

N	0.9344513	-4.5265728	0.0000000
C	-0.5422775	5.7823778	0.0000000
C	-2.2978407	4.0917916	0.0000000
C	-1.3701842	3.1033368	0.0000000
C	0.0132925	3.5083549	0.0000000
H	-2.5685963	6.1381885	0.0000000
N	-1.8956761	5.3841242	0.0000000
O	-0.2828840	6.9757587	0.0000000
N	0.3831945	4.7750487	0.0000000
N	3.2355568	-4.5792723	0.0000000
H	4.1349169	-4.1356771	0.0000000
H	3.1746646	-5.5823325	0.0000000
O	1.3448931	-0.3912846	0.0000000
H	-3.3631836	3.8973660	0.0000000
N	0.9747642	2.5723455	0.0000000
H	0.7851914	1.5787983	0.0000000
H	1.9265577	2.9018876	0.0000000
H	3.0787282	-2.0732072	0.0000000
Br	-4.0305180	-2.9594638	0.0000000
Br	-1.8487964	1.2865940	0.0000000

104 (IC···IG)

C	-0.1607881	-2.3485388	0.0000000
C	-0.1062619	-3.7409653	0.0000000
C	2.0935773	-3.8977736	0.0000000
C	1.0755281	-1.6205154	0.0000000
C	-2.1780060	-3.0063265	0.0000000
N	-1.4621363	-1.9004564	0.0000000
N	-1.4134020	-4.1436813	0.0000000
H	-1.7450275	-5.0985066	0.0000000
N	2.1622063	-2.5315503	0.0000000
N	0.9667395	-4.5635935	0.0000000
C	-0.5089813	5.8438072	0.0000000
C	-2.2889653	4.1804804	0.0000000
C	-1.3807109	3.1715702	0.0000000
C	0.0087222	3.5608110	0.0000000
H	-2.5294728	6.2306665	0.0000000
N	-1.8680982	5.4661477	0.0000000
O	-0.2311499	7.0331685	0.0000000
N	0.3994236	4.8220640	0.0000000
I	-1.9374108	1.1840012	0.0000000
I	-4.2194038	-3.0829823	0.0000000
N	3.2682508	-4.5739855	0.0000000
H	4.1594376	-4.1141078	0.0000000
H	3.2261093	-5.5780459	0.0000000
O	1.3030129	-0.4206847	0.0000000
H	-3.3587332	4.0103515	0.0000000
N	0.9667149	2.6185097	0.0000000
H	0.7737939	1.6262824	0.0000000
H	1.9197674	2.9445777	0.0000000
H	3.0652641	-2.0707241	0.0000000

Cartesian coordinates of the selected PDB examples:

1IJW

N	38.4180000	19.0430000	17.2310000
C	38.6730000	20.1900000	16.5270000
N	38.6970000	20.0000000	15.2350000
C	38.4220000	18.6510000	15.0830000
C	38.3240000	17.8620000	13.9100000
O	38.4480000	18.2200000	12.7280000
N	38.0480000	16.5360000	14.2130000
C	37.8500000	16.0430000	15.4910000
N	37.5550000	14.7330000	15.6060000
N	37.9270000	16.7790000	16.5860000
C	38.2260000	18.0550000	16.3070000
Br	37.4730000	14.5430000	8.0350000
N	37.5890000	12.3350000	11.4990000
C	37.5130000	12.7070000	10.1930000
C	37.6810000	13.3020000	12.5120000
O	37.6980000	12.9240000	13.6860000
N	37.7360000	14.6190000	12.1750000
C	37.6760000	14.9700000	10.8840000
N	37.7290000	16.2780000	10.5820000
C	37.5570000	13.9890000	9.8410000
H	38.8536865	21.1485740	17.0339263
H	37.4804327	14.3973548	16.5649316
H	37.4257255	11.8982020	9.4523810
H	37.9462333	16.9866065	11.3338217
H	38.3218913	18.9278643	18.2402022
H	37.5402034	11.3577331	11.7939656
H	37.6238429	14.0427195	14.8301127
H	37.9500470	15.8603968	13.4173510
H	37.7314720	16.5720171	9.6055137

3BSU

N	-1.6510000	-21.7380000	26.2310000
C	-0.3390000	-21.8700000	25.8320000
N	-0.1380000	-21.5690000	24.5760000
C	-1.3990000	-21.2150000	24.1090000
C	-1.8510000	-20.7900000	22.8520000
N	-1.0410000	-20.6000000	21.8110000
N	-3.1740000	-20.5510000	22.7010000
C	-3.9820000	-20.7270000	23.7600000
N	-3.6670000	-21.1160000	25.0050000
C	-2.3440000	-21.3370000	25.1110000
N	-6.7410000	-19.4590000	19.4530000
C	-6.1620000	-19.8960000	20.6170000
N	-4.7910000	-19.8800000	20.6270000
C	-3.9720000	-19.4330000	19.6100000
C	-4.6440000	-18.9760000	18.4810000
C	-5.9770000	-19.0080000	18.4280000
O	-6.8040000	-20.2440000	21.5590000
O	-2.7660000	-19.4580000	19.7360000
I	-3.4400000	-18.1630000	16.7560000
H	0.4404315	-22.1967268	26.5348851
H	-0.0403149	-20.7507283	21.9176787
H	-5.0548947	-20.5397642	23.5596701
H	-6.5278027	-18.6574702	17.5414602
H	-7.7614884	-19.4613389	19.4152102
H	-2.0582504	-21.9311049	27.1464953
H	-1.4564071	-20.2117525	20.9463186
H	-4.2081448	-20.1945687	21.4808971

3JXR

N	13.6300000	14.5830000	10.3220000
C	12.2670000	14.6910000	10.1490000
N	11.6040000	13.5710000	10.2830000
C	12.6200000	12.6570000	10.5400000

C	12.5520000	11.2690000	10.7920000
O	11.5370000	10.5680000	10.8380000
N	13.8060000	10.7260000	11.0150000
C	15.0050000	11.3830000	10.9930000
N	16.1180000	10.6540000	11.2150000
N	15.0880000	12.7030000	10.7720000
C	13.8630000	13.2420000	10.5620000
N	14.7160000	5.8470000	11.9950000
C	14.4800000	7.2030000	11.6960000
O	15.4340000	7.9430000	11.3900000
N	13.2010000	7.6530000	11.7650000
C	12.2180000	6.8830000	12.1040000
N	11.0260000	7.4670000	12.1430000
C	12.4540000	5.5080000	12.4090000
F	11.3360000	5.1320000	12.9110000
C	13.6920000	5.0520000	12.3310000
H	11.8080292	15.6674103	9.9328537
H	16.0513513	9.6276372	11.2964880
H	10.1677386	6.9650453	12.3543837
H	13.9345942	4.0150663	12.6156580
H	14.3392411	15.3121928	10.2513551
H	15.6697141	5.4949477	11.8971077
H	11.0038127	8.4498648	11.8199171
H	17.0103109	11.1360025	11.1405889
H	13.7775369	9.7081593	11.2145998

3KDE

N	14.2640000	20.3810000	-20.9290000
C	15.5140000	19.7980000	-21.1660000
N	16.6570000	20.5030000	-20.9850000
C	16.6250000	21.7390000	-20.4890000
C	15.3120000	22.3670000	-20.2480000
C	14.1500000	21.6540000	-20.5170000
O	15.5560000	18.6150000	-21.5830000
O	17.6930000	22.3700000	-20.2890000
Br	15.2270000	24.0920000	-19.5960000
N	22.4370000	17.3850000	-22.3760000
C	23.2780000	18.4350000	-22.2060000
N	22.6870000	19.5240000	-21.7840000
C	21.3530000	19.1410000	-21.7010000
C	20.2040000	19.8320000	-21.3460000
N	20.2150000	21.1310000	-20.9810000
N	19.0310000	19.1540000	-21.3940000
C	19.0200000	17.8610000	-21.7430000
N	20.0520000	17.1030000	-22.0870000
C	21.1920000	17.8310000	-22.0530000
H	13.1453062	22.0757807	-20.3730677
H	24.3564076	18.3440984	-22.4000969
H	19.3361458	21.6252797	-20.7151993
H	18.0140810	17.4019865	-21.7529684
H	13.4692917	19.7781777	-21.1492610
H	22.6624931	16.4395014	-22.6880350
H	21.1103865	21.6164612	-20.9713793
H	17.6082185	19.9645466	-21.1490481

4HUG

N	3.3420000	-6.7630000	4.0010000
C	4.6980000	-6.7710000	4.1820000
N	5.3730000	-7.2090000	3.1500000
C	4.4040000	-7.5110000	2.2130000
C	4.4770000	-8.0210000	0.8990000
N	5.6230000	-8.3290000	0.2670000
N	3.3070000	-8.2030000	0.2470000
C	2.1600000	-7.8990000	0.8730000
N	1.9690000	-7.4130000	2.0950000
C	3.1450000	-7.2400000	2.7230000
N	1.6450000	-10.5400000	-3.6940000
C	1.8380000	-9.9660000	-2.4150000

O	0.8900000	-9.9670000	-1.5950000
N	3.0090000	-9.4100000	-2.0670000
C	4.0320000	-9.3940000	-2.9280000
O	5.1300000	-8.8780000	-2.6150000
C	3.8610000	-9.9980000	-4.2720000
C	2.6360000	-10.5670000	-4.5990000
Cl	5.1820000	-9.9780000	-5.3930000
H	5.1493687	-6.4350857	5.1264524
H	6.5066343	-8.1243447	0.7305586
H	1.2557500	-8.1215145	0.2759263
H	2.4460549	-11.0325914	-5.5767448
H	0.7168537	-10.9374640	-3.8448727
H	2.6105363	-6.4664556	4.6476374
H	5.5884830	-8.5427408	-0.7522521
H	3.1351569	-8.9076188	-1.0723590

4XSN

N	15.3390000	11.6340000	32.1990000
C	15.7460000	12.9640000	32.1170000
O	14.8750000	13.8420000	32.1100000
N	17.0590000	13.2110000	32.0560000
C	17.9890000	12.2410000	32.0620000
N	19.2730000	12.5960000	31.9910000
C	17.6080000	10.8670000	32.1330000
C	16.2680000	10.6200000	32.2070000
N	18.8790000	19.8500000	31.9410000
C	20.2260000	19.6400000	31.8330000
N	20.5480000	18.3500000	31.8900000
C	19.3410000	17.7210000	31.8130000
C	18.3150000	18.6180000	31.9580000
N	16.9910000	18.4000000	31.9920000
C	16.7270000	17.1080000	32.0260000
N	15.4240000	16.6640000	32.0770000
N	17.7110000	16.1140000	31.9150000
C	19.0890000	16.3190000	31.9550000
O	19.9120000	15.4050000	31.8260000
Br	21.5510000	20.9920000	31.8630000
H	20.0059173	11.8900486	31.9908159
H	18.3344691	10.0448473	32.1383236
H	15.8636918	9.5985201	32.2713209
H	15.1862723	15.6523117	32.0909900
H	18.3799536	20.7403050	31.9370603
H	14.3350741	11.4501056	32.2500427
H	19.5305632	13.6166409	31.9308423
H	14.7014041	17.3786562	32.1191296
H	17.4144030	15.1044869	31.9460516

5AY3

Br	4.0720000	30.3600000	15.4740000
N	7.1240000	31.2610000	12.8810000
C	6.2910000	30.6220000	13.7500000
C	6.7650000	32.5060000	12.3460000
O	7.5420000	33.0710000	11.5680000
N	5.5820000	33.0630000	12.6940000
C	4.7760000	32.4270000	13.5450000
N	3.6150000	33.0120000	13.8620000
C	5.1260000	31.1630000	14.1040000
N	4.4130000	39.6290000	11.2950000
C	3.0890000	39.7020000	11.6580000
N	2.6050000	38.5550000	12.0570000
C	3.6790000	37.6770000	11.9600000
C	3.7710000	36.2860000	12.2450000
O	2.8940000	35.5220000	12.6610000
N	5.0490000	35.7960000	11.9830000
C	6.0970000	36.5380000	11.5000000
N	7.2440000	35.8750000	11.2740000
N	6.0250000	37.8290000	11.2440000
C	4.7980000	38.3280000	11.4920000
H	6.6416089	29.6617842	14.1558008
H	2.9740862	32.5503365	14.5068105

H	2.5275027	40.6448994	11.5966535
H	7.3322592	34.8418149	11.3391943
H	8.0178090	30.8649992	12.5834820
H	8.0038763	36.4179466	10.8689773
H	5.2084138	34.7762756	12.1788748
H	3.3344782	33.9270928	13.4111607
H	5.0186882	40.3745617	10.9513052

7EDT

N	-3.0300000	16.8340000	16.2440000
C	-3.9200000	16.3320000	17.1610000
N	-3.4040000	15.4090000	17.9380000
C	-2.0900000	15.2860000	17.4990000
C	-1.0070000	14.4820000	17.9080000
N	-1.0700000	13.5860000	18.9020000
N	0.1600000	14.6320000	17.2410000
C	0.2390000	15.5160000	16.2430000
N	-0.7050000	16.3290000	15.7780000
C	-1.8530000	16.1600000	16.4530000
N	4.6390000	12.4920000	17.1240000
C	3.4650000	13.1840000	16.8100000
O	3.3620000	13.8590000	15.7780000
N	2.4140000	13.1310000	17.7210000
C	2.4760000	12.4010000	18.9370000
O	1.4840000	12.4070000	19.6550000
C	3.7110000	11.7230000	19.1590000
C	4.7620000	11.7480000	18.3000000
Br	3.8660000	10.7510000	20.7670000
H	-4.9555687	16.6953921	17.2162222
H	-1.9358304	13.5035416	19.4317393
H	1.2261253	15.5504804	15.7448515
H	5.7099060	11.2235786	18.4765704
H	-3.1847767	17.5485825	15.5321736
H	5.3888753	12.5540816	16.4332180
H	-0.2064285	13.1077861	19.2223021
H	1.5182133	13.7100598	17.5124729

1OMK

N	11.1290000	12.4670000	7.7980000
C	11.5320000	11.1470000	7.7550000
N	10.5290000	10.3010000	7.6870000
C	9.4230000	11.1060000	7.6840000
C	8.0540000	10.8070000	7.6290000
N	7.6010000	9.5430000	7.5550000
N	7.2020000	11.8270000	7.6440000
C	7.6440000	13.0980000	7.7190000
N	8.9310000	13.4970000	7.7820000
C	9.7560000	12.4420000	7.7530000
N	2.4780000	12.2010000	8.2610000
C	3.8690000	12.2910000	8.0970000
N	4.6200000	11.1840000	7.9880000
C	4.0260000	9.9870000	8.0780000
C	2.6350000	9.8810000	8.2340000
C	1.8910000	10.9870000	8.3160000
O	4.3540000	13.4010000	8.0390000
O	4.7920000	8.8990000	7.9840000
I	1.7510000	7.9860000	8.3380000
H	12.5952616	10.8713855	7.7781402
H	8.2952659	8.7982112	7.6094700
H	6.8504352	13.8637007	7.7473379
H	0.7968149	10.9646385	8.4338266
H	6.5871969	9.2968012	7.6641477
H	11.7099930	13.3040428	7.8557654
H	1.9805497	13.0904134	8.3182506
H	5.7103788	11.3990356	7.8200257
